# Integrated plasma metabolomics and lipidomics profiling highlights distinctive signature of hepatocellular carcinoma in HCV patients

**DOI:** 10.1186/s12967-023-04801-4

**Published:** 2023-12-18

**Authors:** Vicky Caponigro, Anna L. Tornesello, Fabrizio Merciai, Danila La Gioia, Emanuela Salviati, Manuela G. Basilicata, Simona Musella, Francesco Izzo, Angelo S. Megna, Luigi Buonaguro, Eduardo Sommella, Franco M. Buonaguro, Maria L. Tornesello, Pietro Campiglia

**Affiliations:** 1 Department of Pharmacy, University of Salerno, Via Giovanni Paolo II 132, Fisciano, SA Italy; 2 Innovative Immunological Models Unit, Istituto Nazionale Tumori IRCCS “Fondazione G. Pascale”, 80131 Naples, Italy; 3 Hepatobiliary Surgical Oncology Unit, Istituto Nazionale Tumori IRCCS “Fondazione G. Pascale”, Naples, Italy; 4 Infectious Disease Unit, A.O. San Pio, PO Rummo, 82100 Benevento, Italy; 5 Molecular Biology and Viral Oncology Unit, Istituto Nazionale Tumori IRCCS “Fondazione G. Pascale”, 80131 Naples, Italy; 6 PhD Program in Drug Discovery and Development, University of Salerno, Fisciano, SA Italy

**Keywords:** Hepatocellular carcinoma (HCC), Hepatitis C virus (HCV), Mass spectrometry, Metabolomics, Lipidomics

## Abstract

**Background:**

Early diagnosis of hepatocellular carcinoma (HCC) is essential towards the improvement of prognosis and patient survival. Circulating markers such as α-fetoprotein (AFP) and micro-RNAs represent useful tools but still have limitations. Identifying new markers can be fundamental to improve both diagnosis and prognosis. In this approach, we harness the potential of metabolomics and lipidomics to uncover potential signatures of HCC.

**Methods:**

A combined untargeted metabolomics and lipidomics plasma profiling of 102 HCV-positive patients was performed by HILIC and RP-UHPLC coupled to Mass Spectrometry. Biochemical parameters of liver function (AST, ALT, GGT) and liver cancer biomarkers (AFP, CA19.9 e CEA) were evaluated by standard assays.

**Results:**

HCC was characterized by an elevation of short and long-chain acylcarnitines, asymmetric dimethylarginine, methylguanine, isoleucylproline and a global reduction of lysophosphatidylcholines. A supervised PLS-DA model showed that the predictive accuracy for HCC class of metabolomics and lipidomics was superior to AFP for the test set (100.00% and 94.40% vs 55.00%). Additionally, the model was applied to HCC patients with AFP values < 20 ng/mL, and, by using only the top 20 variables selected by VIP scores achieved an Area Under Curve (AUC) performance of 0.94.

**Conclusion:**

These exploratory findings highlight how metabo-lipidomics enables the distinction of HCC from chronic HCV conditions. The identified biomarkers have high diagnostic potential and could represent a viable tool to support and assist in HCC diagnosis, including AFP-negative patients.

**Graphical abstract:**

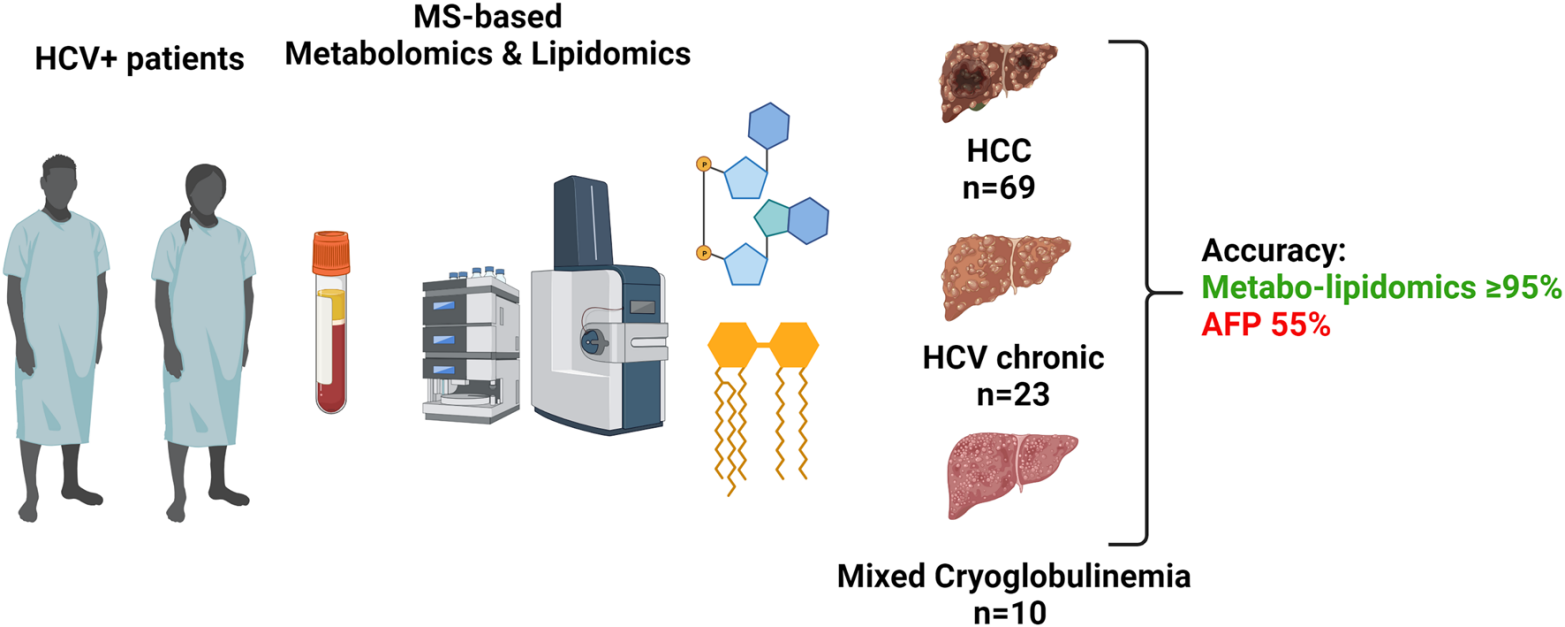

**Supplementary Information:**

The online version contains supplementary material available at 10.1186/s12967-023-04801-4.

## Background

Hepatocellular carcinoma (HCC) is the main common histological type of primary liver cancer constituting roughly 90% of liver malignancies [1, 2]. Besides hepatitis B and C virus (HCB, HCV), the main drivers of HCC are alcohol abuse, non-alcoholic steatohepatitis (NASH) and other factors such as tobacco and aflatoxin. In addition to HCC, HCV may trigger extra-hepatic manifestations such as mixed cryoglobulinemia disease (MC) that, less commonly, could complicate connective tissue diseases, lymphoproliferative disorders, and other chronic infections [3, 4]. Therapeutic strategies for HCC treatment depend on the stage, grade of liver dysfunction and tumor size [5]. Surgical approaches, including tumor resection, local ablation, liver transplantation and trans-arterial chemoembolization are effective in HCC patients at early or intermediate stages [6]. However, the majority of HCCs are diagnosed at late stages and available therapies are tyrosine kinase inhibitors based on the use of sorafenib and lenvatinib in the first line, and the use of cabozantinib and regorafenib in the second line. There is also high expectation for new systemic therapies for HCC such as immunotherapies, monoclonal antibodies, and their combinations which require the identification of biomarkers for patients’ stratification and response prediction [7, 8]. Many HCC cases are diagnosed late since HCC is clinically asymptomatic in its early stage, hence this aspect dramatically lowers the survival rates below 20% [9]. HCC usually develops in patients with chronic HBV/HCV cirrhosis, initiating with dysplastic nodules driving the progression to HCC. The early diagnosis still remains a challenge also for radiology or pathology experts, particularly in the acknowledgement of small lesions (≤ 1 cm), or even more complicated in some patients (e.g., obese) [2]. The largest body of diagnosis is performed through surveillance in individuals at risk, such as with ultrasonography and using established serum markers like α-fetoprotein (AFP) and glypican-3 (GPC3), or alternative strategies based on molecular markers such as liquid biopsy [10, 11]. Nevertheless, these approaches are often affected by low sensitivity and specificity, and the quest for new biomarkers is still open. Ideally, improvements should not only include the diagnosis of HCC vs healthy individuals but also discriminate between people with chronic liver damage at different levels. Additionally, novel biomarkers could also help in prognosis and drug-response monitoring related to the HCC high molecular heterogeneity. To explore more sensitive and specific markers for early and accurate diagnosis of hepatocellular carcinoma, there have been several previous investigations on gene expression [12], miRNA profiles [13], and protein expression [14] of hepatocellular carcinoma. Recently, among omics sciences, metabolomics has provided a new angle for biomarker discovery as a fast, sensitive, and valuable tool to describe the metabolic alteration connected to HCC. It has been used with multiple analytical approaches such as Nuclear Magnetic Resonance (NMR) [15] Gas Chromatography–Mass Spectrometry (GC–MS) [16] and Liquid Chromatography–Mass Spectrometry (LC–MS) [17]. Besides polar metabolome, lipid remodelling is a hallmark of cancer [18] and the concurrent analysis of metabolome and lipidome can enhance the overall potential to highlight molecular changes associated with HCC. In this study, we will show how the combined profiling of plasma metabolites and lipids can lead to identify a molecular signature able to discriminate HCC among patients with chronic HCV infection.

## Methods

### Participants’ characteristics and collection of clinical samples

This is a retrospective study including 102 HCV-positive subjects diagnosed with HCC (n = 69), chronic HCV infection (n = 23), and MC (n = 10). HCC and HCV subjects with chronic infection were enrolled at Istituto Nazionale Tumori “Fondazione G. Pascale”. All HCC patients were in BCLC stage A or stage B and treated by surgical liver resection according to Milan criteria. MC patients were enrolled at the Azienda Ospedaliera San Pio, Benevento. Chronic HCV infection was defined, in agreement with the Centre for Disease Controls and Prevention (CDC) guidelines, as a persistent viremia occurring for greater than 6 months after the initial exposure. The HCV infection was diagnosed by detection of anti-HCV antibodies with third-generation enzyme immunoassay (III generation EIA) against HCV-core and HCV-non-structural antigens and confirmed by detection of HCV RNA (Cobas Amplicor HCV assay, ROCHE). For each patient, the age at diagnosis, gender, HCV status, HCV viral load when available, and diagnosis were recorded. Liver function tests, including Alphafetoprotein (AFP), Carbohydrate antigen 19-9 (CA19-9), Carcinoembryonic antigen (CEA), Alanine aminotransferase (ALT), Aspartate Aminotransferase (AST) and Gamma-glutamyl transferase (GGT), have been also retrospectively collected. Serological testing for tumor biomarkers was carried out with regulatory agencies–approved and commercially available kits according to the manufacturers’ instructions. The upper limits of tumor biomarkers standard reference values were AFP ≤ 20 ng/L, CEA ≤ 3 ng/L, and CA19-9 ≤ 37 U/mL. A diagnosis of MC was based on the detection of cryoglobulins, performed according to guidelines of the “Associazione Italiana per la Lotta alle Crioglobulinemie”. Blood samples were obtained from all HCV-positive subjects before the initiation of any type of treatment including the use of direct acting antivirals. For each patient, 5 mL of whole blood was collected in ethylenediaminetetraacetic acid (EDTA) tubes and processed within 2 h after collection. The plasma samples were obtained by centrifugation at 1200×*g* for 15 min and then stored at − 80 °C. HCC patients were classified according to ChildPugh score into A (n = 40) and B (n = 8). Tumor size and the number of tumor nodules were determined by computed tomography or magnetic resonance imaging. HCC were classified in moderately differentiated (G2 n = 46) and poorly differentiated (G3 n = 2) tumors, according to the histological grade criteria defined by Edmondson and Steiner [19]. The study was approved by the Institutional Scientific Board and by the Ethical Committee of the Istituto Nazionale Tumori “Fondazione G. Pascale” (prot. 51-OSS/21), and it is in accordance with the principles of the Declaration of Helsinki.

### Chemicals

LC–MS-grade Water (H_2_O) acetonitrile (ACN), methanol (CH_3_OH), isopropanol (IPA), 1-butanol (BuOH), methyl tert-butyl ether (MTBE), and additives formic acid (HCOOH), ammonium hydroxide (NH_4_OH), ammonium formate (HCOONH_4_) and ammonium acetate (CH_3_COONH_4_), were all purchased from VWR (Milan, Italy). Deuterated and authentic lipid standards were purchased by Avanti Polar Lipids (Alabaster, AL, U.S.A). Unless stated otherwise other reagents were all purchased by Merck.

### Metabolome and lipidome extraction

Metabolites and lipids were extracted as follows: plasma samples (20 µL) were thawed on ice and 225 µL of ice-cold CH_3_OH, containing a mix of deuterated standards, were added and vortexed for 10 s. Subsequently, 750 µL of cold MTBE were transferred to the tube and the solution was continuously agitated in a thermomixer (Eppendorf, Milan, Italy) for 10 min, 300 rpm at 4 °C. Then, 188 µL of H_2_O were added and samples were shaken for 20 s and centrifuged at 14,680 rpm, for 10 min at 4 °C to induce phase separation. The upper MTBE layer (for lipids) and the lower MeOH/H_2_O (for metabolites) were separately collected and evaporated using a SpeedVac (Savant, Thermo Scientific, Milan, Italy). For the assessment of repeatability and instrument stability over time, a Quality Control (QC) strategy was applied. A QC sample was prepared by pooling the same aliquot (10 µL) from each sample. Samples were injected in randomized order and blank samples were injected regularly and used to assess carryover and exclude background signals. Dried samples were dissolved in 70 µL of ACN/H_2_O 3/1 (v/v %) and in 100 µL of BuOH/IPA/H_2_O 8/23/69 (v/v %), respectively, for metabolomics and lipidomics analysis.

### Instrumentation

Omics analyses were performed on a Thermo Ultimate UHPLC system (Thermo Scientific, Bremen, Germany) coupled online to a TimsTOF Pro Quadrupole Time of Flight (Q-TOF) (Bruker Daltonics, Bremen, Germany) equipped with an Apollo II electrospray ionization (ESI) probe. Detailed instrument parameters are reported in Additional file 1: Section S.1.

### Metabolome and lipidome analysis

Metabolomics analyses were carried out in HILIC mode, while lipidomics analysis by RP-UHPLC, both performed in data dependent acquisition-parallel accumulation serial fragmentation (DDA-PASEF) scan mode. Detailed conditions for LC–MS parameters are described in Additional file 1: Section S1.1. The dataset is available in Zenodo (https://zenodo.org/record/8296815).

### Metabolomics and lipidomics pre-processing

Data alignment, filtering and annotation were performed with MetaboScape 2021 (Bruker) employing a feature-finding algorithm (T-Rex 4D) that automatically extracts buckets from raw files. For both metabolomics and lipidomics analysis feature detection was set to 250 counts for positive and negative modes with a minimum number of data points in the 4D-TIMS space set to 100 and employing a recursive feature extraction tool set to 75 points. Molecular formulas were assigned using Smart Formula™ (SF). Compounds annotation was performed with the following parameters: mass accuracy: narrow 2 ppm, wide 10 ppm; mSigma: narrow 30, wide 250; MS/MS score: narrow 800, wide 150; Collision Cross-Section (CCS) %: narrow 2, wide 3.5. CCS values were compared with those predicted by CCSbase platform [20]. Polar metabolites were annotated using the following libraries of MS-DIAL [21]: MSMS-Public-Pos and MSMS-Public-Neg. Lipids annotation was carried out with both rule-based annotations and the LipidBlast spectral library of MS-DIAL [21]. Lipidomics raw data were deconvoluted in positive mode using [M+H]^+^, [M+Na]^+^, [M+K]^+^, [M+H–H_2_O]^+^ and [M+NH_4_]^+^ ions, while [M−H]^−^, [M+Cl]^−^, [M+HCOO]^−^ and [M−H_2_O]^−^ were in negative mode. Metabolomics spectra were processed in positive mode using [M+H]^+^ as the primary ion and [M+Na]^+^, [M+K]^+^, [M+H–H_2_O]^+^ as seed ions while, in negative mode, [M–H]^−^ was the primary ion and [M+Cl]^−^ and [M−H_2_O]^−^ were the seed ions. All spectra were manually curated and investigated. Subsequently, all metabolites missing in more than 75% of real samples and 50% of QCs samples were excluded. In addition, the polar and non-polar molecules with a Coefficient of Variation (CV) higher than 30% among QCs were discarded.

### Chemometrics and multivariate data analysis

The filtered data were processed and analysed using Matlab R2022b by MathWorks Inc. in Natick, MA, USA. The analysis involved both custom-developed routines and standard Matlab functions for multivariate data analysis. Each dataset was analysed separately, and low-level data fusion was employed as a component of the analysis process [22]. Further information regarding the low-level data fusion can be found in Additional file 1: Section S.2.1 Data Fusion.

#### Data pre-processing

The lipidomics and metabolomics datasets were independently pre-processed. Metabolomics datasets were normalized by the total ion sum in Matlab, while lipidomics data were normalized against class-specific internal standards. For missing values and zeros, one-fifth of the minimum value for the target molecule in the dataset was used for replacement. After that, base 10 logarithms were calculated for the values. Subsequently, the data were scaled using autoscaling, which involved centering each variable by subtracting its mean and then dividing by its standard deviation. These pre-processing steps were completed before further chemometric modelling were performed.

#### Chemometrics

Exploratory analysis was conducted independently on each data block using Principal Component Analysis (PCA) after column autoscaling. SUM-PCA was performed fusing the data blocks [22, 23]. PCA is an unsupervised algorithm used to analyse and reduce the dimensionality of high-dimensional datasets, revealing important features or principal components (PCs). To visualize initial discrimination effectiveness, Hotelling (T^2^) confidence ellipses were added to score plots for each class. These T^2^ confidence ellipses, calculated independently for each class, had a confidence level of 95%. To ensure the reliability and comparability of classification models across all omics modalities, the Kennard-Stone algorithm (KS algorithm) was applied to define common training and test sets for all omics modalities. This algorithm divides data into training and test sets based on sample distances, further information can be found in Additional file 1: Section S.2.2 Kennard-Stone algorithm. The KS algorithm was applied to the T^sup^ of each class, i.e., SUM-PCA was calculated independently for each class set to identify 70% of the data for each class as the training set, and the remaining 30% was identified as the test set [24].

Two independent classification models, Partial Least Square Discriminant Analysis (PLS-DA) [25] and Soft Independent Modelling of Class Analogy (SIMCA), were employed for each modality [26, 27]. The optimal number of latent variables (LV) and PCs was determined through cross-validation (leave one out) to minimize misclassification errors and maximize accuracy. Model evaluation involved confusion matrices, True Positive (TP), i.e., the number of correctly classified samples, True Negative (TN), the sum of misclassified samples for that specific class, are used to determine the correctness of the class predictions. On the other hand, False Positive (FP), (the sum of other class members classified in our class) and False Negative (FN) (the sum of samples not belonging to our class or not classified in our class) values to assess class predictions. Sensitivity, specificity, and accuracy were calculated for each class.

Additionally, loadings and Variable Importance in Projection (VIPs) scores were analysed to identify relevant molecules and compared to p-values obtained from the N-way ANalysis of VAriance (ANOVA). The correlation of these variables with age was computed reporting the significance of this value. For more details about the chemometrics approach, refer to Additional file 1: Section S.2.3 Classification algorithms.

## Results

### Clinical characteristics of the study population

The characteristics of the population are reported in Table 1. The majority of patients in the HCC and HCV groups were males (71% and 61%, respectively, p = 0.3354), with a mean age of 69 and 57 years, most of HCC patients were generally older than 65 years (p < 0.0001). The HCV infection was the main cause of HCC. 48 out of 21 HCC patients were diagnosed with single tumor nodules. In the same group, all patients presented a G2 tumor differentiation. The biochemical analysis showed that there was no statistically significant increase in the levels of AST and AFP, while ALT and GGT increased (p < 0.0196, p < 0.001). Also, there was no statistically significant correlation between the serum level of AFP and any of the patient or tumor characteristics (number of nodules and size of the lesion), as reported also by Carr et al. [28]. The modest correlation of low levels (> 20 ng/mL) of AFP with small HCC lesions, stimulated the search for other biomarkers in order to increase the sensitivity levels of early HCC detection [29, 30]. AFP was not determined in MC patients.

**Table 1:** Laboratory results and clinical data of HCC, HCV and MC patients (*p-values were calculated using one-way ANOVA), nd: not detected, N/A: not applicable

Patient characteristics	HCC (n = 69)	HCV (n = 23)	MC (n = 10)	p-value*
Age (years), n (%)				
≤ 65	17 (25%)	20 (87%)	4 (40%)	< 0.0001
> 65	52 (75%)	3 (13%)	6 (60%)	
Gender, n (%)				
Male	49 (71%)	14 (61%)	5 (50%)	0.3354
Female	20 (29%)	9 (39%)	5 (50%)	
AFP (ng/mL), n (%)				
≤ 20	34 (49%)	22 (95%)	10 (100%)	0.4973
> 20	35 (51%)	1 (5%)	0	
CA19-9 (U/mL), n (%)				
≤ 37	41 (59%)	nd	nd	
> 37	28 (41%)	nd	nd	
CEA (ng/mL), n (%)				
≤ 3	24 (35%)	nd	nd	
> 3	45 (65%)	nd	nd	
ALT (U/L), n (%)				
≤ 33	6 (9%)	nd	4 (40%)	0.0196
> 33	63 (91%)	nd	6 (60%)	
AST (U/L), n (%)				
≤ 32	6 (9%)	nd	2 (20%)	0.266
> 32	63 (91%)	nd	8 (80%)	
GGT (U/L), n (%)				
≤ 40	17 (25%)	nd	7 (70%)	0.0071
> 40	52 (75%)	nd	3 (30%)	
Tumor size (cm), n (%)				
≤ 4	46 (67%)	N/A	N/A	
> 4	23 (33%)	N/A	N/A	
Tumor nodules, n (%)				
Single	48 (69.6%)	N/A	N/A	
Multiple	21 (30.4%)	N/A	N/A	
Tumor differentiation, n (%)				
G2	69 (100%)	N/A	N/A	
G3	0	N/A	N/A	
Child Pugh, n (%)				
A	60 (87%)	N/A	N/A	
B	9 (13%)	N/A	N/A	

### Untargeted metabo-lipidomics profiling

To provide a plasma metabo-lipidomic profile two dedicated mass spectrometry strategies were employed. For metabolome analysis, we used HILIC-HRMS, as a suitable strategy to analyze polar metabolites not sufficiently retained in RP [31] while for lipidomics, a previously optimized RP-UHPLC-HRMS strategy was employed [32]. Metabolite and lipid annotations were performed following the metabolomics and lipidomics standard initiative guidelines [33, 34]. The initial workflow started from 15,757 and 3488 features, respectively, for metabolomics and lipidomics. Each feature was subjected to several filters: mass accuracy (∆ppm: max 5.0 ppm), collision cross section error values (∆CCS: max 3%), peak shape, most probable adduct form, MS/MS spectral similarity score, RT and CCS values linearity, carryover, and coefficient of variation (CV%) < 30% in QCs. Specifically, given the crucial aspects of lipid annotation [35], each lipid species was manually curated to evaluate: (a) lipid adducts in electrospray ionization; (b) regular retention behaviour, e.g., the equivalent carbon number (ECN) model used for RPLC; (c) MS2 spectrum quality, if MS2 spectra contained fragments related only to the lipid class we proposed lipids as short-hand form, otherwise, if in MS2 spectra were present fragment related to the fatty acyl chains we annotated lipids in the long-hand form. In this regard, Additional file 1: Section S3 reports some examples of the described workflow.

After manual curation, a total of 280 compounds were annotated with high confidence (Additional file 1: Tables S1 and S2), covering several classes and subclasses as illustrated in Additional file 1: Fig. S1. Remarkably, the median MS/MS score, ∆m/z [ppm] and ∆CCS [%] were respectively: 899.18 MS/MS score, − 0.21 Δppm, 1.35 ΔCCS. Moreover, 90.57% of polar metabolites showed CV% values < 20%. The principal component analysis score plot (Additional file 1: Fig. S2) shows that pooled QCs samples are correctly grouped, which indicates good stability of the system during the batch.

### Multi-omics data integration and explorative analysis

**Fig. 1:**
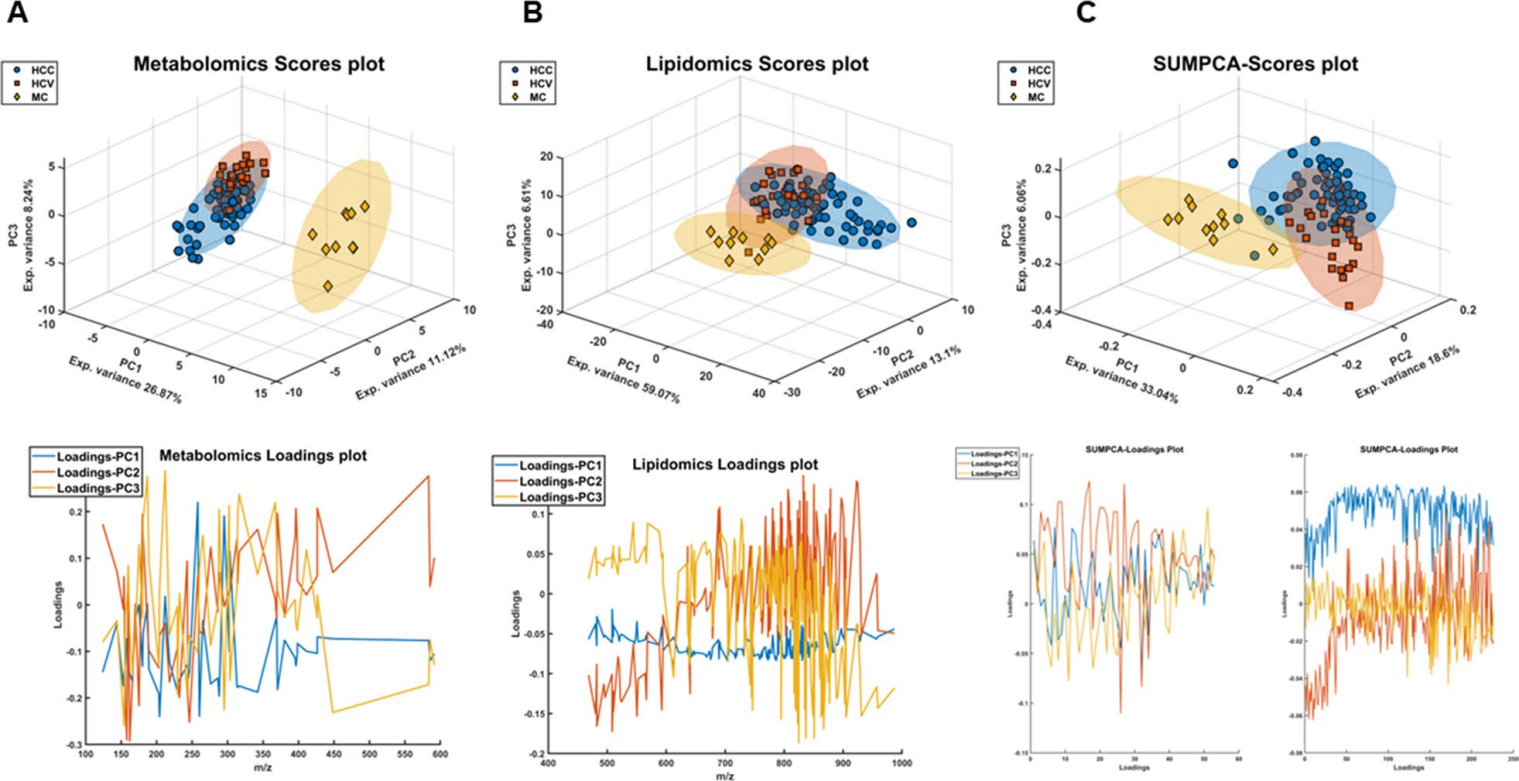
**A**–**C** PCA scores plot PC1, PC2 and PC3 of metabolomics (**A**) and lipidomics (**B**) datasets with confidence ellipses (95%) for each class. **C** SUMPCA of metabolomic and lipidomics datasets, the panel reports the super scores (Tsup) plot for PC1, PC2 and PC3

An exploratory analysis was performed by conducting a Principal Component Analysis (PCA) on the pre-processed and auto-scaled data sets. The score and loadings plots for metabolomics and lipidomics are depicted in Fig. 1A, B, and the bi-dimensional scores and loadings plots are provided in Additional file 1: Figs. S3–S5. There is a distinct separation between MC and HCC-HCV samples in both modalities, whereas HCC and HCV exhibit a similar trend in both cases, indicating a seemingly similar metabo-lipidomic profile. The integration of information from both omics techniques slightly improves the separation between HCC and HCV as well as with respect to the MC group (Fig. 1C). This can be explained by the combination of the information deriving from both metabolome and lipidome profiles that allows to enhance class separation.

### SIMCA and PLS-DA performances comparison to classify HCV-related disease

The analysis involved the independent application of Partial Least Square Discriminant Analysis (PLS-DA) and Soft Independent Modelling of Class Analogy (SIMCA) to each dataset. The purpose of comparing the two models was to emphasize distinct aspects of the problem. PLS-DA focuses on discrimination, as it can characterize and differentiate between three categories representing various grades of HCV-related diseases. On the other hand, SIMCA primarily addresses the challenge of distinguishing a specific category from all the other categories. Table 2 offers a comparative analysis of these two methods, spotlighting their performance across various datasets and classes. The table provides data on the sensitivity and specificity attained through cross-validation and testing for each method including the number of latent variables employed in PLS-DA and principal components in SIMCA. The outcomes indicate that the effectiveness of both methods varies depending on the dataset and class under analysis. As expected, based on the algorithm nature, PLS-DA generally displayed better performance in terms of specificity and overall accuracy in several cases, while SIMCA exhibited higher sensitivity in specific scenarios. The confusion matrices for the test set are reported in Additional file 1: Figs. S6 and S7 for PLS-DA and SIMCA (interpreted as discriminant approach) respectively.

**Table 2:** Values of SIMCA and PLS-DA performances

Method	Classes	SIMCA					PLS-DA				
		PCs	Sensitivity CV (%)	Specificity CV (%)	Sensitivity test (%)	Specificity test (%)	LVs	Sensitivity CV (%)	Specificity CV (%)	Sensitivity test (%)	Specificity test (%)
Lipidomics	HCC	4	91.84	70.92	100.00	66.67	3	93.88	100.00	85.00	88.89
	MC	1	57.14	100.00	100.00	100.00		100.00	100.00	100.00	100.00
	HCV	3	94.12	82.25	100.00	69.57		100.00	94.64	83.33	86.96
Metabolomics	HCC	5	83.67	50.00	95.00	33.33	2	93.88	95.83	100.00	100.00
	MC	1	28.57	100.00	100.00	100.00		100.00	100.00	100.00	100.00
	HCV	4	47.06	97.79	100.00	91.30		94.12	94.64	100.00	100.00

It reports the values of sensitivity and specificity for both cross-validation (leave one out) and test sets

PCs principal components, CV cross validation, LVs latent variables

### Differential metabolites and lipids

**Fig. 2:**
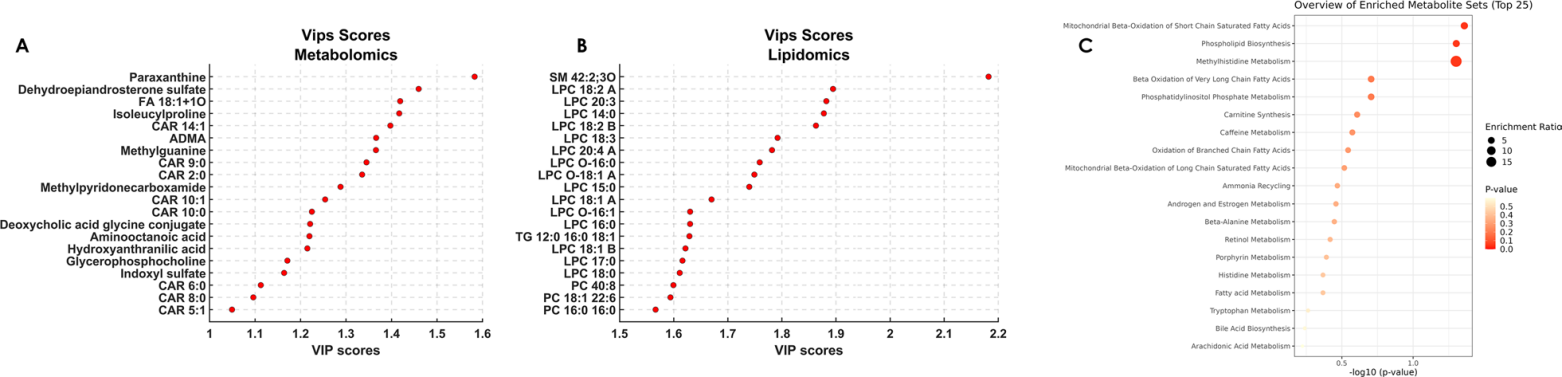
**A**–**C** Top 20 VIP metabolites (**A**) and lipids (**B**) from the PLS-DA model. **C** Pathway enrichment analysis, including the metabolites differentially expressed in patients with HCC, HCV and MC. The red dot colour intensity on the x-axis reflects the P value; the dot size axis represents the enrichment ratio weight

**Fig. 3:**
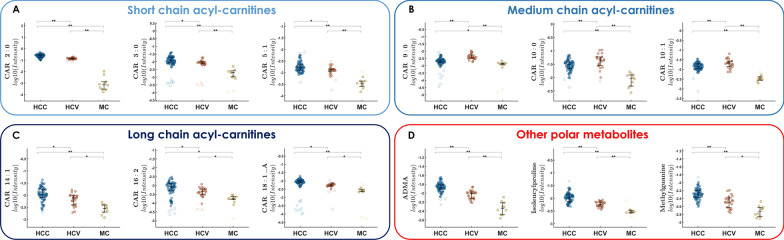
**A**–**D** Distribution in HCC, HCV and MC patients of short (**A**), medium (**B**) and long-chain (**C**) acylcarnitine (CAR) and **D** Ile-Pro, ADMA and MG, where *p-value < 0.05 and **p-value < 0.01

**Fig. 4:**
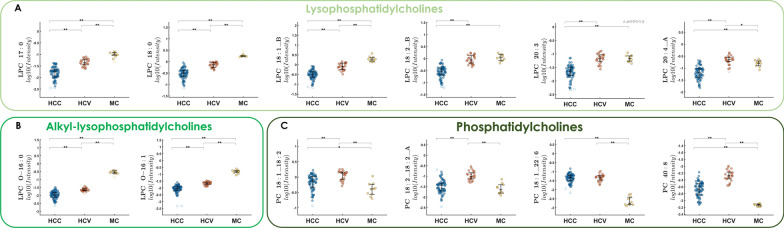
**A**–**C** Distribution in HCC, HCV and MC patients of **A** lysophosphatydilcholines (LPCs), **B** ether-linked lysophosphatydilcholine (LPC-O) and **C** phosphatydilcholine (PC), where *p-value < 0.05 and **p-value < 0.01

From the analysis of the VIP scores obtained from PLS-DA for both metabolomics and lipidomics, it was possible to extrapolate the most important molecules for classification; the top 20 compounds can be visualized in Fig. 2A, B. Among them, different classes of compounds could be observed mainly represented by aminoacids and derivatives, dipeptides, purine derivatives, acylcarnitines, lysophosphatidilcholines and alkyl-lysophosphatidilcholines. Besides the first 20 metabolites and lipids with the highest VIP scores, to further enrich the differential compounds responsible for the differences in the metabolic profile between HCC, HCV and MC classes, univariate analysis was performed, reporting the significant (*p-value* < 0.05) metabolites and lipids (Table 3) and their HMDB codes were employed to build a pathway enrichment analysis. The dot plot in Fig. 2C reports the overview of the enriched metabolite sets, where the size of the dots per metabolite set represents the Enrichment Ratio and the colour intensity represents the p-value, Additional file 1: Table S3 reports the metabolites belonging to the metabolite sets and their p-value. Two top significant pathways (*p-value* < 0.05) emerged, namely Mitochondrial Beta-Oxidation of Short Chain Saturated Fatty Acids (*p* = 0.0437) and Phospholipid Biosynthesis (*p* = 0.0498). Mitochondrial fatty acid oxidation (mtFAO) is a key metabolic pathway required for energy production in the liver, which is tightly connected with the carnitine shuttle. In this context, metabolome analysis pointed out a significant modulation of several acylcarnitines, with a different trend related to the acyl chain across the three conditions. In particular, short-chain (CAR 2:0, CAR 3:0, CAR 5:1) and long-chain acylcarnitines (CAR 14:1, CAR 16:2, CAR 18:1) levels were higher in HCC with respect to both HCV and MC class (Fig. 3A, B). On the contrary, medium-chain acylcarnitines (CAR 9:0, CAR 10:0, CAR 10:1) showed higher abundance in the HCV class with respect to HCC and MC (Fig. 3C). Other polar metabolites such as the dipeptide isoleucylproline (Ile-Pro), the metabolic by-product asymmetric dimethylarginine (ADMA), and the modified purine methylguanine (MG) were found to be significantly changed across the three groups. Their highest level in HCC patients was found, followed by HCV, being considerably lower in the MC group (Fig. 3D). Besides mtFAO, phospholipid biosynthesis emerged as second enriched pathway. Phospholipids synthesis in liver, and especially phosphatidylcholines (PCs), accounts for over 70% of the plasma very low density lipoprotein (VLDL), and PCs are metabolized in Lysophosphatidylcholines (LPCs). In this regard, concerning lipidome, interestingly the profile of LPCs, both saturated (LPC 17:0, LPC 18:0) and unsaturated (LPC 18:1, LPC 18:2, LPC 18:3, LPC 20:3, LPC 20:4, Fig. 4A) as well as alkyl-lysophospatidylcholines (LPC O-16:0, LPC O-16:1, Fig. 4B) was dramatically reduced in HCC patients respect to both HCV and MC. On the contrary, the levels of numerous phosphatidylcholines such as PC 18:1_22:6, PC 16:1:18:2_A, PC 18:1_18:2, PC 40:8 (Fig. 4C) were slightly higher in the HCV group, but both HCC and HCV showed higher values than the MC group. An exploration into the relationship between variables values and age has been undertaken. Specifically in Additional file 1: Tables S4 and S5 highlight a comprehensive overview regarding the most noteworthy variables with VIP scores (> 1) for metabolomics and lipidomics, respectively. These tables report the significance of these variables, showcasing their VIP scores alongside *p-values* obtained through ANOVA calculations as well as the computation of correlation coefficients. Notably, all values remain below 0.63 for metabolome and 0.47 for lipidome, indicating no significant correlation with age on the variables employed for the model.

**Table 3:** Significant metabolites and lipids annotated according to variable importance in projection (VIP) scores (from PLS-DA) and p-value (one-way ANOVA)

Compound	m/z	Primary adduct	Molecular formula	VIP value	p-value	HMDB CODE	KEGG
Metabolites							
Paraxanthine	181.07201	[M+H]^+^	C_7_H_8_N_4_O_2_	1.5920	2.19^E−05^	HMDB0001860	C13747
FA 18:1;1O	295.22836	[M−H]^−^	C_18_H_34_O_3_	1.5692	4.18^E−28^	HMDB0030981	
Dehydroepiandrosterone sulfate	367.15889	[M−H]^−^	C_19_H_28_O_5_S	1.5499	4.18^E−40^	HMDB0001032	C04555
Isoleucylproline	229.15457	[M+H]^+^	C_11_H_20_N_2_O_3_	1.5264	1.98^E−07^	HMDB0011174	
Asymmetric dimethylarginine	203.15007	[M+H]^+^	C_8_H_18_N_4_O_2_	1.4982	7.12^E−15^	HMDB0001539	C03626
Methylguanine	166.07248	[M+H]^+^	C_6_H_7_N_5_O	1.4922	1.09^E−09^	HMDB0003282	C04152
CAR 9:0	302.23251	[M+H]^+^	C_16_H_31_NO_4_	1.4194	1.88^E−08^	HMDB0013288	
Methylpyridonecarboxamide	153.06585	[M+H]^+^	C_7_H_8_N_2_O	1.3698	1.88^E−08^	HMDB0004194	
CAR 10:1	314.23275	[M+H]^+^	C_17_H_31_NO_4_	1.3474	5.90^E−08^	HMDB0250918	
CAR 2:0	204.12322	[M+H]^+^	C_9_H_17_NO_4_	1.3381	6.71^E−19^	HMDB0240773	C02571
CAR 10:0	316.24831	[M+H]^+^	C_17_H_33_NO_4_	1.3152	4.28^E−07^	HMDB0000651	C03299
Deoxycholic acid glycine conjugate	448.30692	[M−H]^−^	C_26_H_43_NO_5_	1.3128	8.75^E−04^	HMDB0000631	C05464
Hydroxyanthranilic acid	154.05006	[M+H]^+^	C_7_H_7_NO_3_	1.3118	2.28^E−07^	HMDB0001476	C00632
Indoxyl sulfate	212.00253	[M−H]^−^	C_8_H_7_NO_4_S	1.2672	2.14^E−04^	HMDB0000682	
CAR 6:0	260.18546	[M+H]^+^	C_13_H_26_NO_4_	1.2195	5.61^E−10^	HMDB0000705	
CAR 8:0	288.21724	[M+H]^+^	C_15_H_29_NO_4_	1.2038	1.87^E−06^	HMDB0000791	C02838
Glycerophosphocholine	258.11014	[M+H]^+^	C_8_H_20_NO_6_P	1.1740	3.90^E−61^	HMDB0000086	C00670
CAR 5:1	244.15388	[M+H]^+^	C_12_H_21_NO_4_	1.1487	1.23^E−04^	HMDB0241656	
Aminooctanoic acid	160.13322	[M+H]^+^	C_8_H_17_NO_2_	1.1487	1.18^E−04^	HMDB0247418	
Histidine	156.07681	[M+H]^+^	C_6_H_9_N_3_O_2_	1.1124	7.08^E−05^	HMDB0000177	C00135
Lipids							
SM 42:2;3O	829.67813	[M+H]^+^	C_47_H_93_N_2_O_7_P	2.1819	1.71^E−02^	HMDB0013469	C00550
LPC 18:2_A	520.34014	[M+H]^+^	C_26_H_50_NO_7_P	1.8945	1.68^E−22^	HMDB0010386	C04100
LPC 20:3	546.355	[M+H]^+^	C_28_H_52_NO_7_P	1.8821	2.85^E−21^	HMDB0010393	C03916
LPC 14:0	468.30896	[M+H]^+^	C_22_H_46_NO_7_P	1.8775	3.96^E−20^	HMDB0010379	C03916
LPC 18:2_B	564.33085	[M+HCOO]^−^	C_26_H_50_NO_7_P	1.8629	6.72^E−09^	HMDB0010386	C03916
LPC 18:3	518.32392	[M+H]^+^	C_26_H_48_NO_7_P	1.7920	5.64^E−17^	HMDB0010387	C03916
LPC 20:4_A	544.33999	[M+H]^+^	C_28_H_50_NO_7_P	1.7817	4.99^E−18^	HMDB0010396	C03916
LPC O-16:0	482.36072	[M+H]^+^	C_24_H_52_NO_6_P	1.7589	2.85^E−21^	HMDB0243890	C13903
LPC O-18:1_A	508.37644	[M+H]^+^	C_26_H_54_NO_6_P	1.7491	5.65^E−50^	HMDB0013122	C04317
LPC 15:0	482.32461	[M+H]^+^	C_23_H_48_NO_7_P	1.7397	2.28^E−20^	HMDB0010381	C04230
LPC 18:1_A	522.3558	[M+H]^+^	C_26_H_52_NO_7_P	1.6699	2.26^E−32^	HMDB0010385	C04230
LPC O-16:1	480.34516	[M+H]^+^	C_24_H_50_NO_6_P	1.6303	3.35^E−50^	HMDB0010407	C04317
LPC 16:0	496.33988	[M+H]^+^	C_24_H_50_NO_7_P	1.6302	1.14^E−35^	HMDB0010382	C04233
TG 12:0_16:0_18:1	794.72192	[M+NH_4_]^+^	C_49_H_92_O_6_	1.6290	1.20^E−03^		C00422
LPC 18:1_B	566.34631	[M+HCOO]^−^	C_26_H_52_NO_7_P	1.6217	4.87^E−30^	HMDB0010385	C04230
LPC 17:0	510.35577	[M+H]^+^	C_25_H_52_NO_7_P	1.6162	3.80^E−31^	HMDB0012108	C04230
LPC 18:0	524.37124	[M+H]^+^	C_26_H_54_NO7P	1.6111	1.58^E−40^	HMDB0010384	C04230
PC 40:8	830.56883	[M+H]^+^	C_48_H_80_NO_8_P	1.5994	1.98^E−18^	HMDB0008443	C00157
PC 18:1_22:6	832.58371	[M+H]^+^	C_48_H_82_NO_8_P	1.5939	1.04^E−07^	HMDB0008123	C1387
PC 16:0_16:0	734.56896	[M+H]^+^	C_40_H_80_NO_8_P	1.5665	6.52^E−01^	HMDB0000564	C00157

### AFP classification vs omics PLS-DA models

In the context of correctly classifying HCC, the accuracy values provided by the two omics approaches are compared with the accuracy of AFP prediction. To compare the performance of the omics model and the AFP prediction, the AFP dataset was divided into training and test set in the same way as the lipidomics and metabolomics datasets. HCC samples with AFP values < 20 ng/mL were considered misclassified. The confusion matrices for this dataset are reported in Additional file 1: Fig. S8. The PLS-DA model was built to distinguish AFP-negative HCC patients from those with HCV and MC. Table 4 presents the accuracy values obtained from the PLS-DA model; it is evident that both the metabolomics and lipidomics datasets, when combined with PLS-DA, outperform the AFP approach. In the case of AFP, the accuracy is approximately 50%, with 48.98% for the training set and 55.00% for the test set. On the contrary, the metabolomics dataset achieves 97.90% accuracy for training and a perfect 100.00% accuracy for the test set. Slightly lower values were obtained for lipidomics, with accuracy rates higher than 94% for both the training and test sets. Furthermore, to gain insights into the underlying mechanisms of the PLS-DA models, the most important variables were identified for each method. VIP scores were examined specifically for PLS-DA.

**Table 4:** PLS-DA and AFP accuracy for HCC sample classification

LVs	PLS-DA							
	Lipidomics			Metabolomics			AFP	
	Accuracy training (%)	Accuracy CV (%)	Accuracy test (%)	LVs	Accuracy training (%)	Accuracy test (%)	Accuracy training (%)	Accuracy test (%)
3	100.00		94.40	4	97.90	100.00	48.98	55.00

*CV* cross validation, *LVs* latent variables

### Harnessing omics PLS-DA models for AFP negative HCC classification

**Fig. 5:**
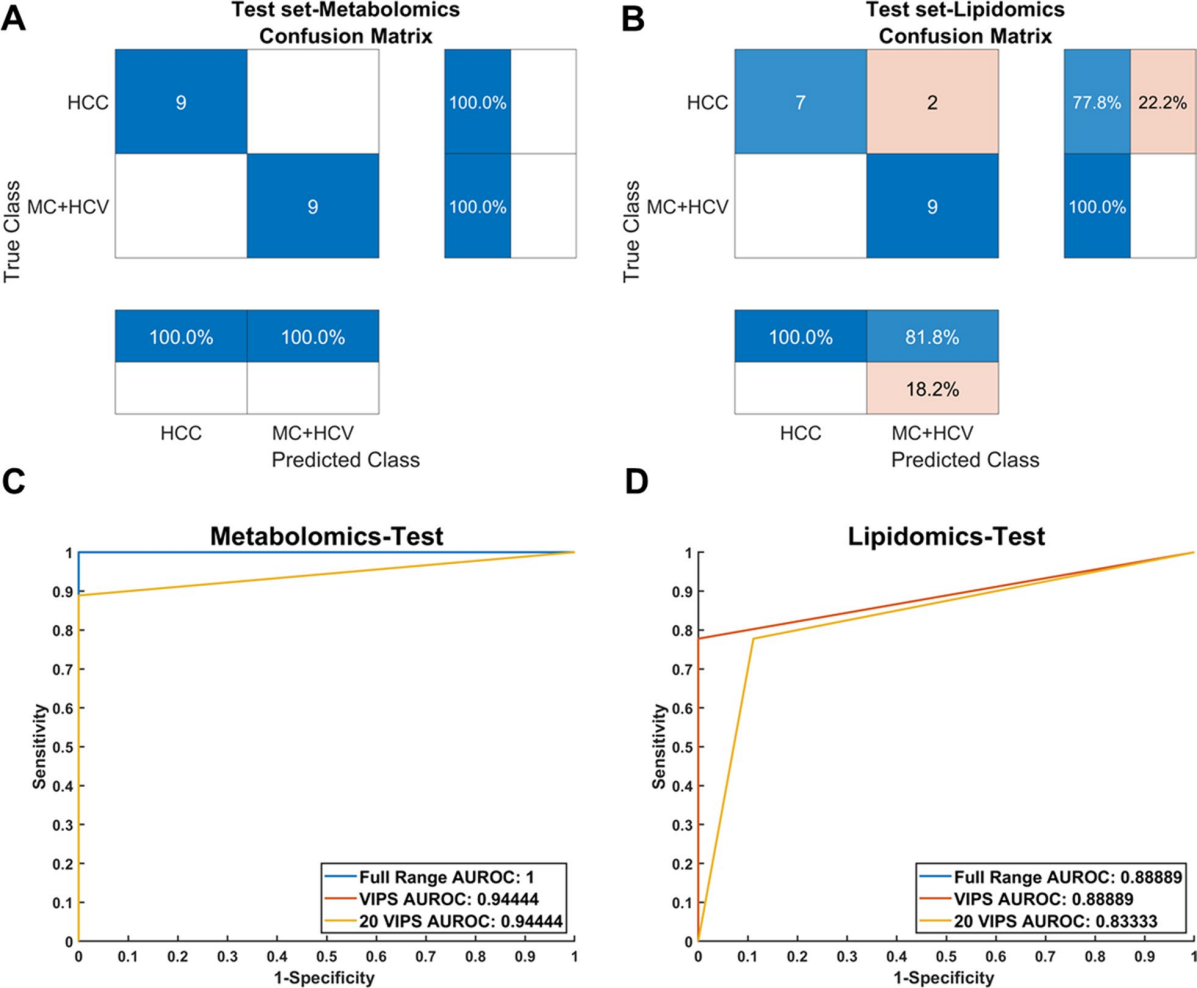
**A**–**D** Graphical representation of confusion matrices obtained from PLS-DA models (metabolomics **A**, lipidomics **B**) of both independent modalities for the test phase classifying AFP-negative HCC patients. The ROC curves (metabolomics **C**, lipidomics **D**) reported compares the models’ performances reducing the number of variables to the VIPs and the first 20 VIPs

The confusion matrices presented in Fig. 5A, B exhibited an overall accuracy of 100.00% for metabolomics, while slightly lower performance was observed for lipidomics with 88.89% accuracy. Comparing the diagnostic performance of the entire datasets with those using only the top 20 VIP metabolites and lipids, it becomes evident that they significantly outperformed AFP. The metabolomics AUROC values were 0.94 for both all the VIPs (> 1) and, remarkably, using only the first 20 VIPs achieved the same value. For lipidomics, the respective AUROC values were 0.89 and 0.83 (Fig. 5C, D). These results highlight the potential of this approach, even when reducing considerably the number of variables and focusing solely on the most significant ones.

## Discussion

The identification of systematically altered metabolic targets is an imperative step toward exploiting metabolism in basic, translational, and clinical cancer studies. While genomic and epigenomic alterations have been associated with liver cancer [7] several shreds of evidence highlight that the tumor onset and progression are strongly characterized by metabolic reprogramming, such as central carbon metabolism, glycolysis, de novo lipogenesis, phosphatidylcholine synthesis [36]. Targeting these mechanisms could represent both a therapeutic and diagnostic opportunity, and the combination of metabolomics and lipidomics holds a great potential for the development of non-invasive diagnostics and tailored therapy for HCC patients [37]. In this study, 102 HCV-positive patients were included, comprising 23 HCV, 69 HCC and 10 MC subjects. The combined metabolomic and lipidomic approach helped clarify the metabolic features clustering within the three different groups. It also allowed the recording of significant differences in the metabolic profile of HCC compared to the HCV and MC classes. Acylcarnitines play an important role in the transport of fatty acids into mitochondria during β-oxidation, and in cases of high energy demand, that in cancer occur. The metabolic reprogramming observed in cancer is recognized for its role in regulating acylcarnitine levels across different chain lengths. It serves as a crucial mediator in cancer metabolic plasticity, intricately connecting essential pathways, factors, and metabolites to fulfill the energetic requirements of cancer cells [38]. As the main organ responsible for endogenous carnitine synthesis and metabolism, the liver can experience notable changes in its acylcarnitine levels. Consequently, these variations may be closely linked to different stages of liver disease [39]**.** Accordingly, the alteration of acylcarnitines level has been associated with HCC. In particular, short-chain (CAR 2:0, CAR 3:0, CAR 5:1) and long-chain acylcarnitines (CAR 14:1, 16:2, 18:1) levels were found increased in HCC with respect to both HCV and MC classes. Increased levels of long-chain acyl carnitines C14:1 and C18:1 have been reported in patients with NAFLD driven liver fibrosis and, additionally, even higher levels in the progression to HCC were found [40]. On the contrary, a higher abundance of medium-chain acylcarnitines (CAR 9:0, CAR 10:0, CAR 10:1) was observed in HCV class with respect to HCC and MC, in good accordance with previous findings [40]. A similar trend has been observed in Chinese cohorts, also being able to discriminate across severe liver disease (CIR) and HCC [41, 42]. These results confirm the potential of acylcarnitines as potential biomarkers also for AFP false-negative HCC patients [40], clearly a correlation of plasma and tissue levels would be further necessary to extend these findings. Within polar metabolites, three additional metabolites emerged as significant across the three classes, and globally elevated in HCC: ADMA, MG and the dipeptide Ile-Pro. ADMA is a byproduct of proteolysis of post-translationally methylated proteins, which has been found to increase in the plasma of cancer patients [43]. Indeed, high plasma ADMA levels have been traced in patients with liver cirrhosis, alcoholic hepatitis and acute liver failure [44]. MG is a modified purine derivative and has been associated with rapid turnover of nucleic acids which increases in the plasma and urine of cancer patients [45, 46]. Dipeptides have been recently investigated as potential markers of different cancer, including hepatocellular carcinoma, showing a different profile in tumor and non-tumor tissues [45, 47]. Alteration in lipid homeostasis represents an important hallmark for different diseases, especially cancer. Different lipid subclasses have been found altered in HCC. Notably, investigations into the lipidomic profiles of HCC have frequently noted a reduction in glycerophospholipids (GPLs) that incorporate polyunsaturated fatty acids (PUFAs), including arachidonic acid (C20:4), within human HCC specimens [48]. Noteworthy, in our study LPCs containing PUFA were among the main lipid subclasses found reduced in the plasma of HCC patients. Recently the enzyme Lysophosphatidylcholine acyltransferase 1 (LPCAT1) has emerged as a novel diagnostic marker in HCC, being overexpressed in different cancers, including HCC [49, 50]. LPCAT1 catalyzes the conversion of lysophosphatidylcholines into phosphatidylcholines. Notably, the lipidomic analysis of this study highlights a marked reduction in the overall LPCs profile, especially in the HCC group. This reduction is followed to a lesser extent by HCV and differs from MC. Interestingly, this reduction is independent of fatty acyl composition, indicating an upregulation of the Land’s cycle, resulting in further incorporation of fatty acids into PC. Our results are consistent with previous work carried out with an international cohort, illustrating a significant reduction in LPC in the serum of patient [51]. On the contrary, the levels of different PCs have increased in both HCV and HCC groups with respect to MC. The latter confirms the potential alteration of the LPCAT activity, that induces the conversion of LPCs to PCs which is essential for tumorigenesis and promotes cancer cell growth and metastases [51, 52]. Certainly, further exploration is warranted given the extensive diversity of lipid species. While the prospect of identifying non-invasive circulating lipid biomarkers for HCC is exciting, the remodeling of tissue lipids in tumor and non-tumor liver tissues could reveal pathways in HCC pathogenesis, whether related to viral factors or other mechanisms. Establishing correlations between plasma and tissue lipid changes, where possible, is crucial for gaining mechanistic insights into the regulation of lipid metabolism and homeostasis during the development and progression of liver cancer. Such insights may pave the way for the potential development of therapies targeting lipid pathways [53]. Despite the growing interest in exploring lipidomic changes in liver cancer, attempts to target lipid metabolism in a therapeutic context have not yielded success. The dynamic nature of the lipidome, coupled with a lack of mechanistic insights into the specific role(s) of individual lipids in liver cancer development, poses a significant obstacle to the development of novel therapies. While our existing knowledge enables the utilization of the human lipidome as a non-invasive diagnostic and prognostic tool, it is imperative to delve. With respect to other studies that have been mainly focused on comparing tumor-free healthy controls vs HCC patients [15, 54], the present study demonstrates how the developed model, based on the combined metabolome and lipidome signatures, shows high classification accuracy for distinguishing HCC vs other HCV related disease. Furthermore, it was able to outperform AFP accuracy, especially for patients with values below 20 ng/mL [55], thus underlying its exploitability in the frame of diagnosis and prognosis of HCV-related landscape. Future studies with larger cohorts including different racial, ethnic, and geographical cohorts will be necessary for extending our current findings.

## Conclusions

Overall, our results contribute to shedding light on the metabolome and lipidome alterations in the plasma of HCC and HCV chronic patients and provide useful information towards the quest for new biomarkers in HCCs diagnosis. AFP measurement is one of the most used screening tests to diagnose HCC. However, it has limited sensitivity and specificity since early lesions may not release AFP and cirrhotic liver may produce high levels of AFP. The comprehension of molecular mechanisms in HCC could be exploited not only in diagnosis but also in evaluating the potential evolutionary trajectories that tumor will follow and hence select optimal therapies to maximize clinical benefit. The availability of new biomarkers meets an urgent medical need and is considered pivotal for improving the effectiveness of specific treatments and consequently the patient's survival rate. This study can be considered useful in investigating the potential use of new biomarkers by exploiting them for an early diagnosis. From a clinical standpoint, the panel of metabolites and lipids identified in this pilot study could be translated in a targeted assay, which could be implemented on widely diffused triple quadrupole MS-platforms in clinical setting, providing absolute quantitation. These values could serve as innovative risk-factors for HCC early detection, contributing to more timely and effective treatments. Currently we are developing a high-throughput targeted LC–MS method on the best performing metabolites/lipids identified through untargeted approach. This method will be used for a subsequent large-scale application and evaluate their diagnostic accuracy also in AFP-negative patients.

### Supplementary Information

**Additional file 1: **Supplementary material reports complete data for statistical treatment and metabolite and lipid annotation parameters.

## Data Availability

Raw data associated with MS method are available at https://zenodo.org/record/8296815.
