# Supplementary material for: Integrated plasma metabolomics and lipidomics profiling highlights distinctive signature of hepatocellular carcinoma in HCV patients
Source: J Transl Med. 2023 Dec 18;21:918. doi: 10.1186/s12967-023-04801-4 (PMC10729519; doi:10.1186/s12967-023-04801-4)
Supplement: Supplementary file 1 — Additional file 1: Supplementary material reports complete data for statistical treatment and metabolite and lipid annotation parameters. [file 12967_2023_4801_MOESM1_ESM.docx]

**ADDITIONAL FILE FOR THE MANUSCRIPT**

**Integrated plasma metabolomics and lipidomics profiling highlights distinctive signature of Hepatocellular carcinoma in HCV patients**

Vicky Caponigro^1#^, Anna L. Tornesello^2#^, Fabrizio Merciai^1^, Danila La Gioia^1^, Emanuela Salviati^1^, Manuela G. Basilicata^1^, Simona Musella^1^, Francesco Izzo^3^, Angelo S. Megna^4^, Luigi Buonaguro^5^, Eduardo Sommella^1*^, Franco M. Buonaguro^2^, Maria L. Tornesello^2^, Pietro Campiglia^1^

**S.1 Untargeted metabolomics and lipidomics profiling**

Omics analyses were performed on a Thermo Ultimate RS 3000, which comprised a RS 3000 autosampler, a column oven and a binary pump with a 35 µL mixer. All connections were Viper (Thermo Fisher Scientific) stainless steel capillaries (0.100 mm I.D.). The column outlet was connected to the MS source with a peek tubing (0.100 mm I.D.) of the shortest length possible. The UHPLC system was coupled online to a TimsTOF Pro Quadrupole Time of Flight (Q-TOF) (Bruker Daltonics, Bremen, Germany) equipped with an Apollo II electrospray ionization (ESI) probe. The instrument was calibrated for both mass and mobility using the ESI-L Low Concentration Tuning Mix with the following composition: [m/z, 1/K_0_: (322.0481, 0.7318 Vs cm ^−2^), (622.0290, 0.9848 Vs cm^−2^), (922.0098, 1.1895 Vs cm^−2^), (1221.9906, 1.3820 Vs cm ^−2^)] in positive mode and [m/z, 1/K_0_: (301.99814, 0.6678 Vs cm^−2^), (601.97897, 0.8781 Vs cm^−2^), (1033.98811, 1.2525 Vs cm^−2^), (1333.96894, 1.4015 Vs cm^−2^)] in negative mode. Before each LC-MS run a mixture (1:1 *v/v %*) of 10 mM sodium formate calibrant solution and ESI-L Low Concentration Tuning Mix was injected to recalibrate, respectively, the mass and mobility data.

In detail, m etabolome analyses were performed in HILIC mode, with an Acquity BEH Amide (150 × 2.1 mm; 1.7 μm) protected with a VanGuard amide precolumn (5 × 2.1 mm; 1.7 μm) (Waters, Milford, MA, U.S.A). The column temperature was set at 45 °C, and the flow rate was 0.350 mL/min. The mobile phase was (A): H_2_O/ACN 95/5 (*v/v %*) and (B): in H_2_O/ACN 5/ 95 (*v/v %*) both buffered with 10 mM CH_3_COONH_4_ plus 0.1% HCOOH (*v/v %*) for positive ionization while 10 mM CH_3_COONH_4_ plus 0.1% NH_4_OH (*v/v %*, pH=8) additives were used for negative mode. The following gradient was employed: 0–0.1 min, 99 % B; 0.1–8 min, 99–50 % B; 8.0–8.5 min, 50-30 % B; 8.5-9.5 min isocratic at 30 % B; returning to 99% in 0.1 min, and then 4 min to recondition the column. The TIMS-MS analyses were performed in Data-Dependent Parallel Accumulation Serial Fragmentation (DDA-PASEF) with both positive and negative ionization in separate runs. 3 μL and 5 μL were injected for ESI^+^ and ESI^-^ analysis, respectively. Source parameters: nebulizer gas (N_2_) pressure: 3.0 bar, dry gas (N_2_): 10 L/min, dry temperature: 220°C. Mass spectra were recorded in the range m/z 50–1000, with an accumulation and ramp time of 100 ms each. The ion mobility was scanned from 0.45 to 1.45 Vs/cm^2^. Precursors for data-dependent acquisition were isolated within ± 2 m/z and fragmented with a TIMS-STEPPING ion mobility-dependent collision energy mode: CE [eV] #1: 50 and CE [eV] #2: 20. The total acquisition cycle was of 0.53 s and comprised one full TIMS-MS scan and two PASEF ramps. Exclusion time was set to 0.1 min, and Ion Charge Control (ICC) was set to 7.5 Mio.

Lipidome analyses were performed with an Acquity UPLC CSH^TM^ C18 column (50 × 2.1 mm; 1.7 μm, 130 Å) protected with a VanGuard CSH^TM^ precolumn (5.0 × 2.1 mm; 1.7 μm, 130 Å) (Waters, Milford, MA, U.S.A). The column temperature was set at 65 °C, a flow rate of 0.55 mL/min was used, mobile phase consisted of (A): ACN/H_2_O 60:40 (*v/v %*) and (B): IPA/ACN 90:10 (*v/v %*) both buffered with 10 mM HCOONH_4_ and 0.1% HCOOH. The following gradient has been used: 0 min, 40% B; 0.4 min, 43% B; 0.425 min, 50% B; 0.9 min, 57% B; 2.0 min, 70% B; 2.950 min, 99% B; 3.3 min, 99% B; 3.301 min, 40% B and then 0.7 min for column re-equilibration. The TIMS-MS analyses were performed in DDA-PASEF with both positive and negative ionization, in separate runs. The injection volume was set at 2 µL and 4 µL for ESI^+^ and ESI^-^, respectively. Source parameters: Nebulizer gas (N_2_) pressure: 3.0 Bar, Dry gas (N_2_): 10 L/min, Dry temperature: 280°C. Mass spectra were recorded in the range m/z 100–1500, with an accumulation and ramp time of 100 ms each. The ion mobility was scanned from 0.55 to 1.70 Vs cm^-2^. Precursors for data-dependent acquisition were isolated within ± 2 m/z and fragmented with a TIMS-STEPPING ion mobility-dependent collision energy mode: CE [eV] #1: 20-40 and CE [eV] #2: 35-50. The total acquisition cycle was 0.53 s and comprised one full TIMS-MS scan and two PASEF.

**S.2 Data Analysis**

**S.2.1 Data Fusion**

Low-level data fusion is a chemometric tool that integrates multiple sources of information (multi-blocks) to extract the maximum information from different omics approaches. This optimization enhances the model performance in terms of accuracy, consistency, and robustness. In this experiment, the same biological sample was analyzed using different omics methods, and the original pre-processed data, which had the same sampling mode, were concatenated row by row (low level) [1].

**S.2.2 Kennard-Stone algorithm**

The Kennard-Stone algorithm is a widely used technique for selecting training data from a large multivariate dataset with a uniform distribution, covering the entire range of the dataset, including its boundaries. It provides a good balance between representativeness and efficiency. It is a repeatable approach because the starting point is constant using the inter-sample distance over the predictor space. Then, in each iteration, it selects the sample that is farthest from the already selected samples. This process is repeated until the desired number of samples is selected. This process is repeated until the desired number of samples is selected. It is possible to summarise the algorithm in four steps:

I. Compute the pairwise distances between all samples in the dataset.

II. The two samples that are farthest apart using Euclidean or Mahalanobis distance are added to the training set.

III. For each of the remaining samples, the minimum distance to each of the selected training samples is calculated.

IV. The candidate sample with the maximum minimum distance, as calculated in step 2, is added to the training set. Steps 2 and 3 are repeated until the desired number of training samples, as determined by the user, is reached. All the remaining samples form the test set.

Steps III. And IV. are repeated until the desired number of training samples (user-defined) is selected. All the remaining samples will form the test set [2].

**S.2.3 Classification Algorithms**

Partial Least Squares Discriminant Analysis (PLS-DA) and Soft Independent Modeling of Class Analogy (SIMCA) are two multivariate statistical methods used for classification and pattern recognition. PLS-DA belongs to the discriminant approach category, while SIMCA is a class-modelling technique. Discriminant approaches aim to distinguish between samples from different classes and divide the variable hyperspace into as many regions as there are categories while class-modelling techniques focus on the similarities within a class and define the category space of one class at a time, rather than the differences between classes. This results in discriminant analysis providing unique assignments of class membership, while class-modelling approaches may assign a sample to a class region or to an intersection of multiple regions, resulting in confusion. Furthermore, class-modelling approaches take into account the possibility that an object being investigated does not belong to any of the modelled classes, whereas discriminant approaches do not allow for this.

Data preprocessing is a crucial step for both techniques. The data is preprocessed to remove noise, scale the variables and center the data. This is done to ensure that each variable is given equal importance in the analysis.

PLS-DA is based on PLS regression applied to a binary-coded categorical variable Y for classification purposes. Given the matrix X whose rows contain the independent spectra, PLS-DA defines the best relationship between X and the categorical dummy response. The categorical information can be expressed as a binary matrix Y (coded as 0 and 1) describing class membership. Indeed, for the four classes, each spectrum belonging to the first class is coded as [1 0 0 0], the second class as [0 1 0 0] and so on... Finally, the PLS kernel is used to compute a regression model that relates the predictor matrix and Y. Classification is then based on linear discriminant analysis (LDA) applied to the predicted Y or to the PLS values. To identify the most important molecules, the variable importance in the projection (VIP) index is used to express the significance of a predictor in defining the F latent vectors model for predicting a specific response. The squared VIP scores average 1, and the "greater than one rule" criterion is commonly used to identify the most significant variables. Examining the regression coefficients of the PLS model can further improve result interpretation by indicating whether variable values for samples from a particular category are higher or lower than those of samples from all other categories.

The class modelling approach involves modelling each category independently of the others. SIMCA, for instance, describes each class based on a principal component model with a specific dimensionality. Once the principal component model is computed, the class space is defined using a statistically defined criterion for outlier detection. Two statistical variables, T^2^ and Q, are used to express a sample's degree of outlyingness with respect to the computed principal component model. T^2^ accounts for the distance of the sample within the model space, while Q represents its distance from the model space. The scores matrix TA and the residual matrix E are used to estimate the values of these two statistics for the analyzed samples. The "reduced distance" between each sample and the model of a category is then computed. Threshold values are selected based on percentiles of the distributions, usually 95% under the null hypothesis. Typically, if a sample's reduced distance exceeds √2, it is considered an outlier and rejected by the class model and, on the other hand, if the distance is lower than this value, the sample is accepted and recognized as belonging to that class.


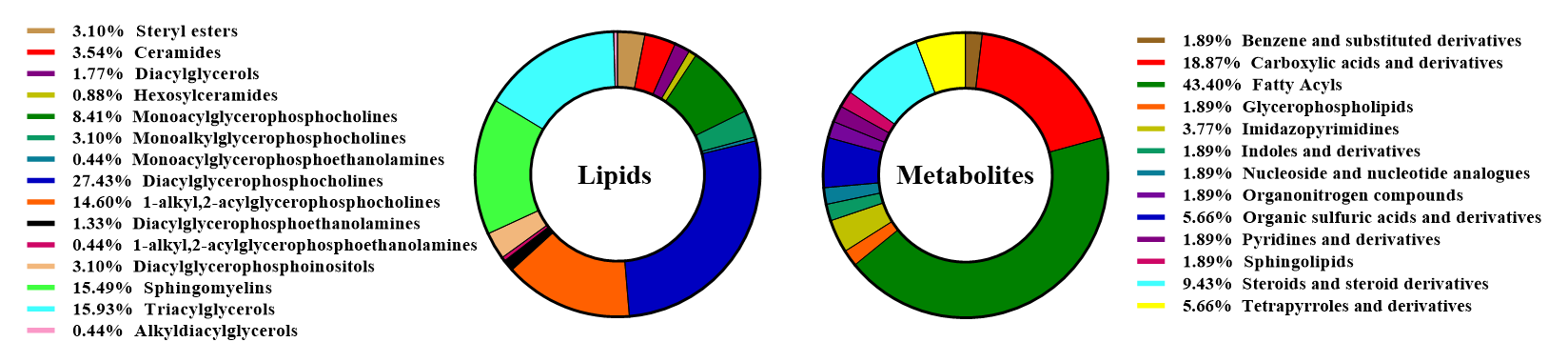


**Figure S1.** Graphical representation of distribution and class information for metabolites (HMDB) and lipids (LIPID MAPS) detected by UHPLC-TIMS-Q-TOF analysis in HCC, HCV and MC plasma samples.


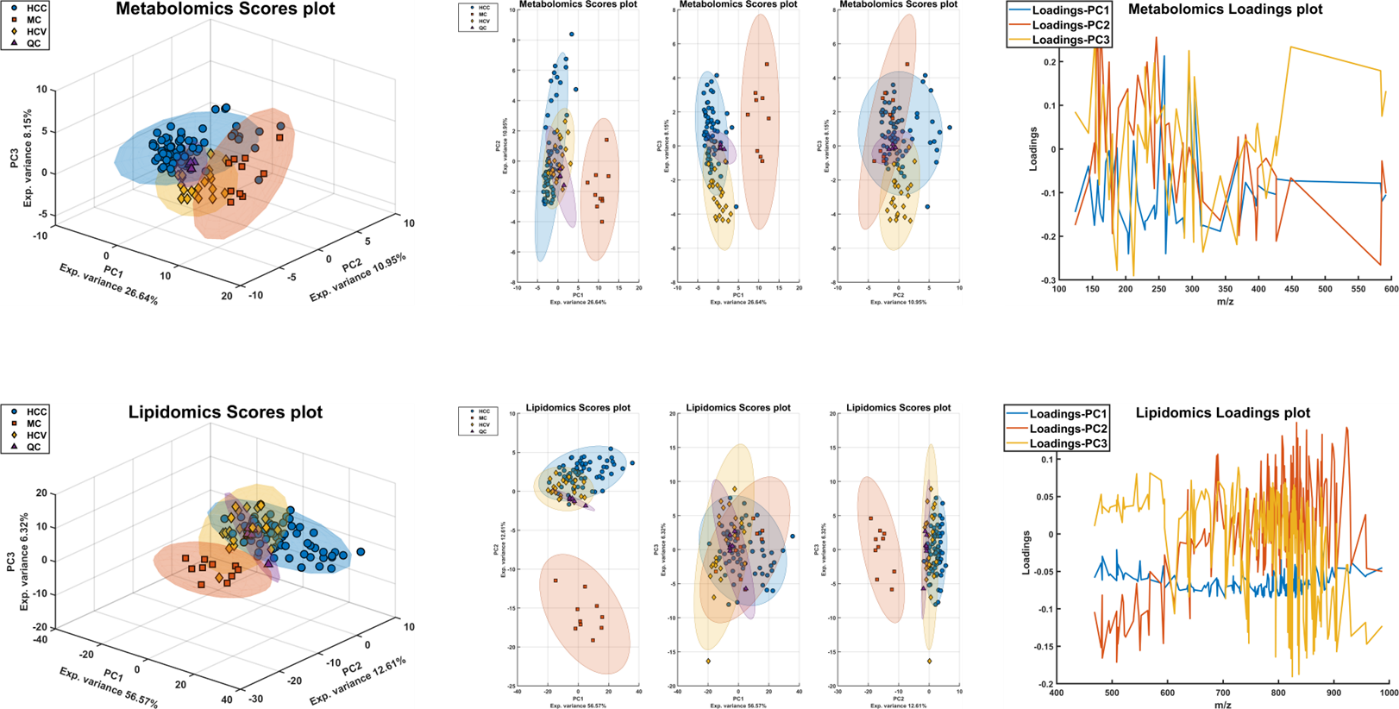


**Figure S2.** PCA scores and loadings plots (PC1, PC2 and PC3) of metabolomics and lipidomics datasets including QC with confidence ellipses (95%) for each class. The datasets were pre-processed by 1) normalization using internal standard 2) the missing values and zeros were replaced with one-fifth of the minimum value recorded in the data set for that molecule 3) logarithm values of the base of 10 and 4) autoscaling.


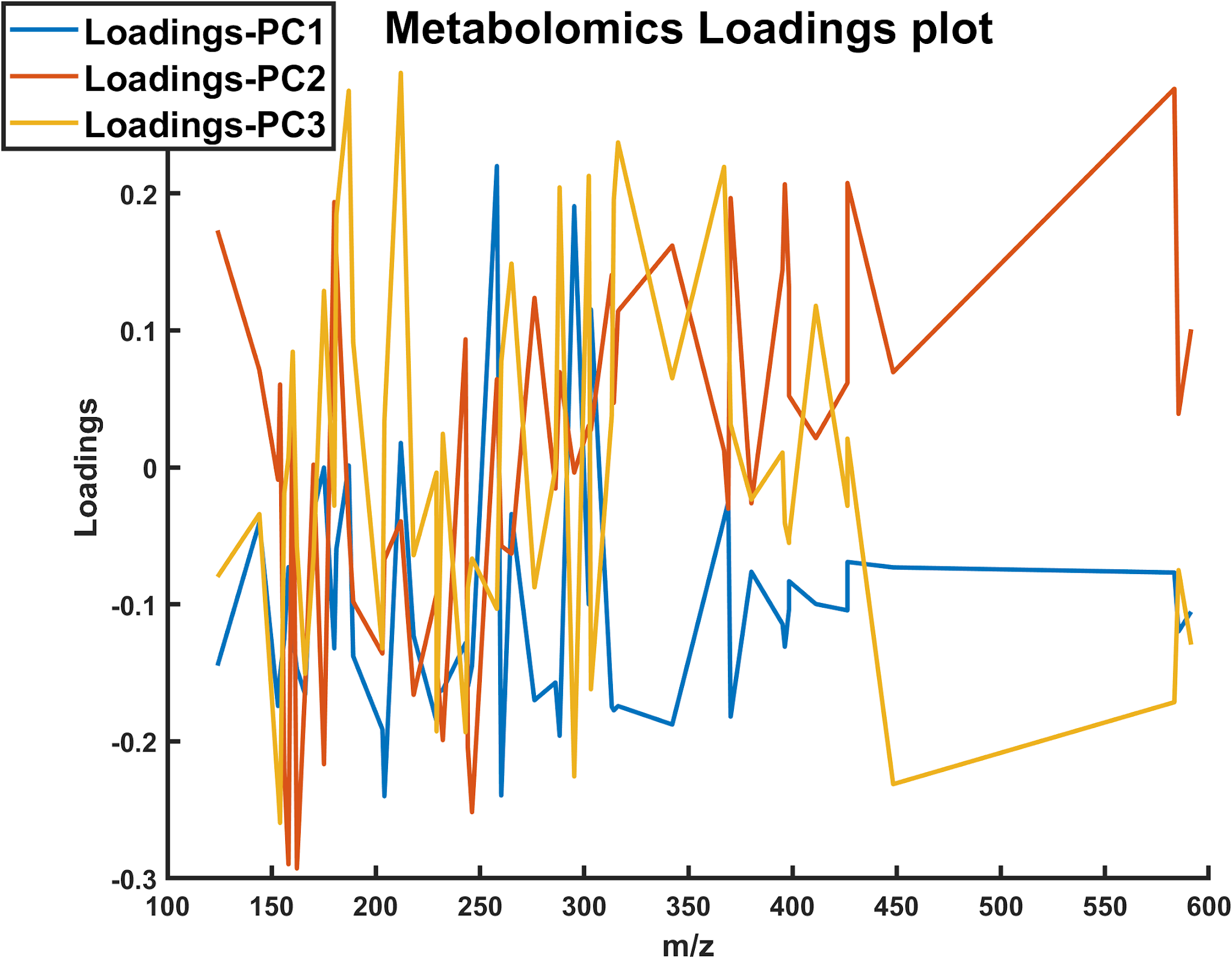

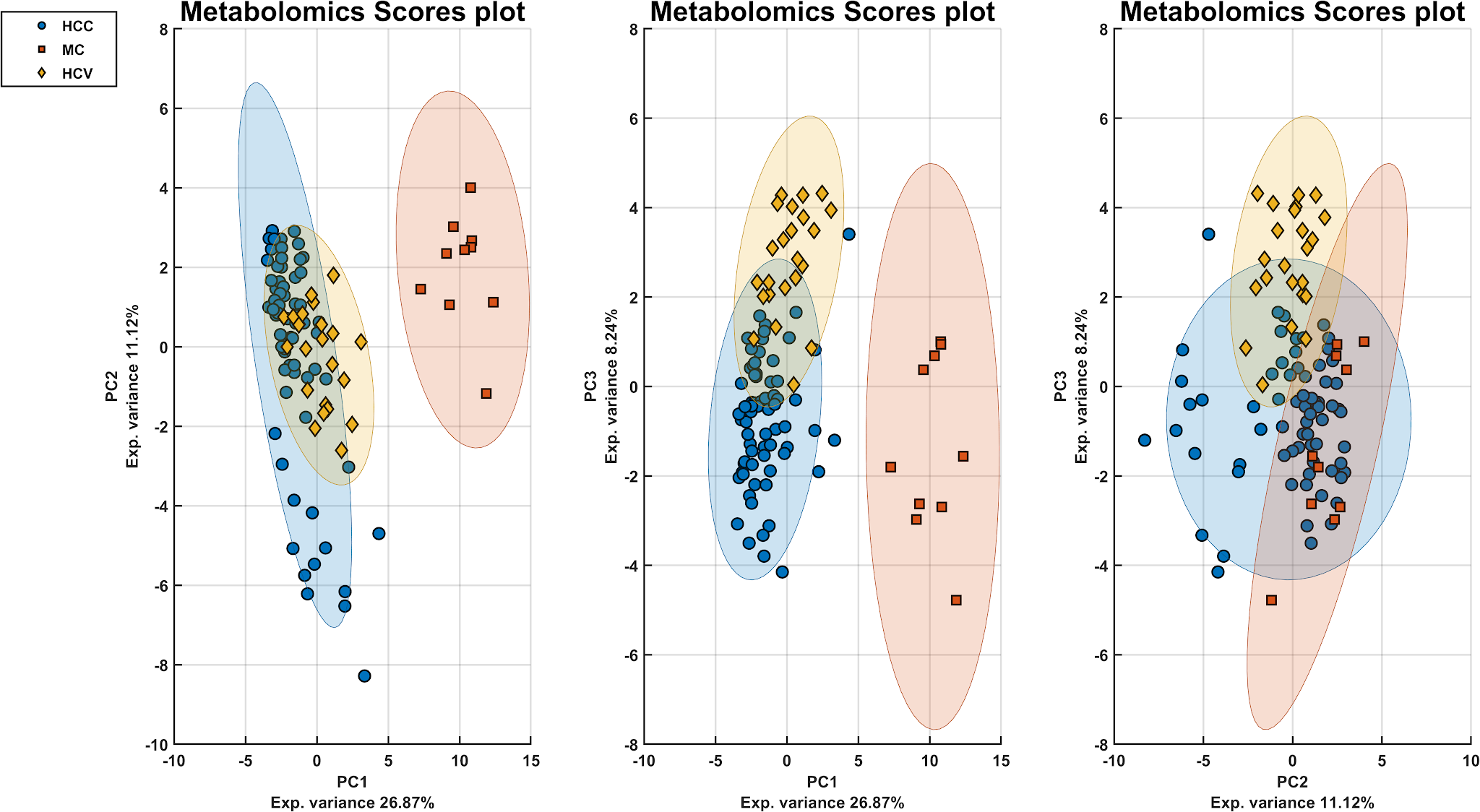


**Figure S3.** PCA bidimensional scores and loadings plots (PC1, PC2 and PC3) of metabolomics, datasets with confidence ellipses (95%) for each class. The datasets were pre-processed by 1) normalization by the total sum ion count 2) the missing values and zeros were replaced with one-fifth of the minimum value recorded in the data set for that molecule 3) logarithm values of the base of 10 and 4) autoscaling.


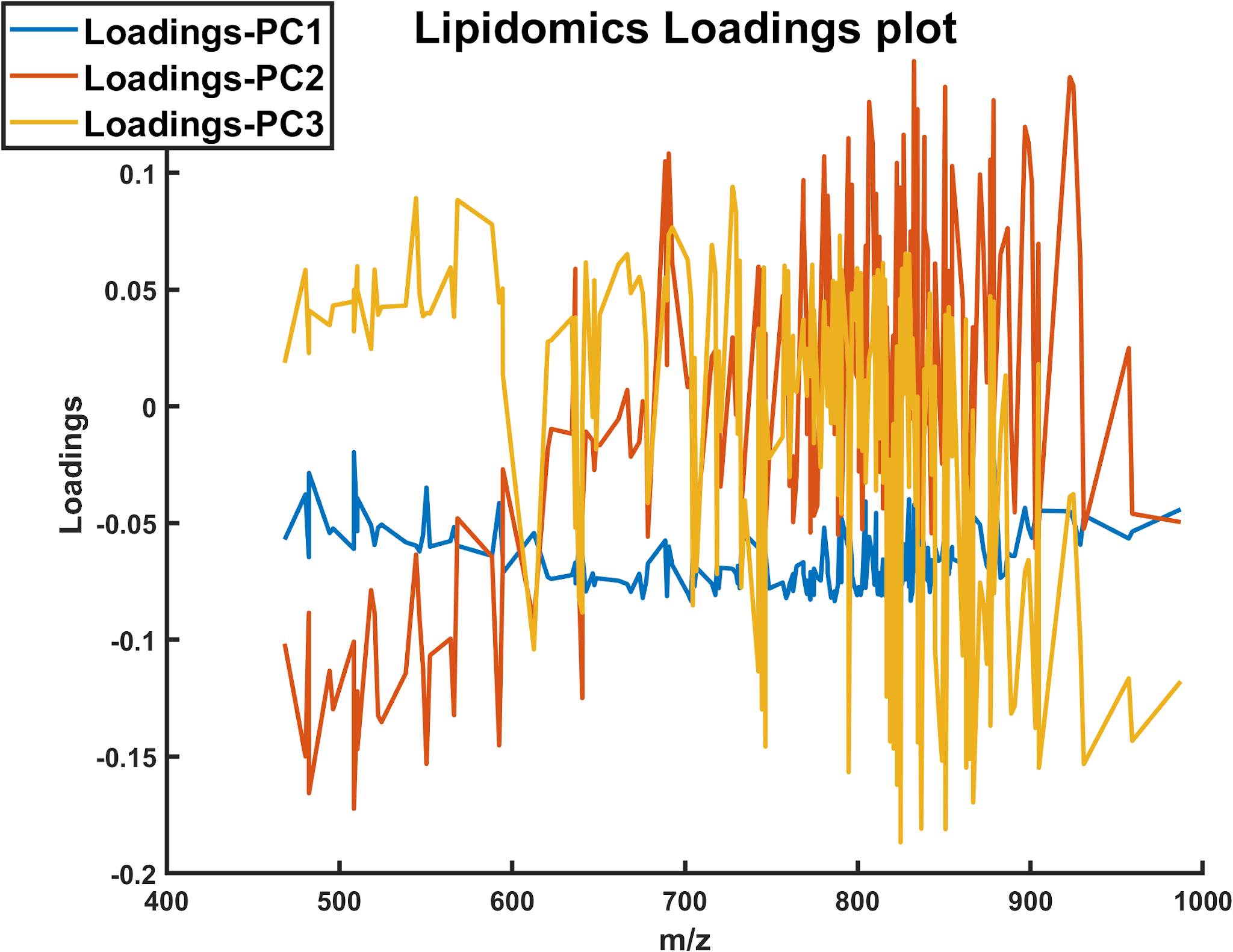

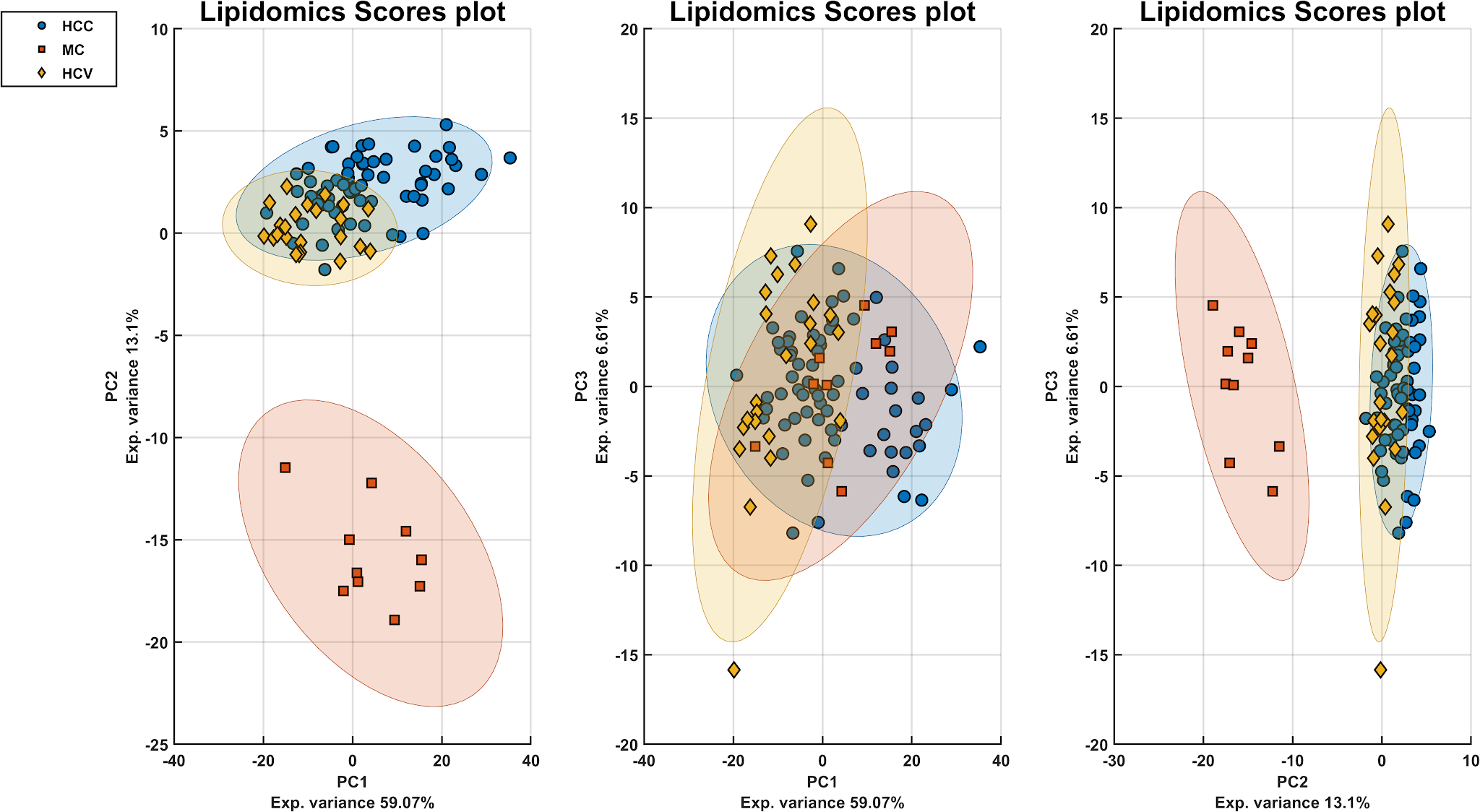


**Figure S4.** PCA bidimensional scores and loadings plots (PC1, PC2 and PC3) of lipidomics, datasets with confidence ellipses (95%) for each class. The datasets were pre-processed by 1) normalization using internal standard 2) the missing values and zeros were replaced with one-fifth of the minimum value recorded in the data set for that molecule 3) logarithm values of the base of 10 and 4) autoscaling.


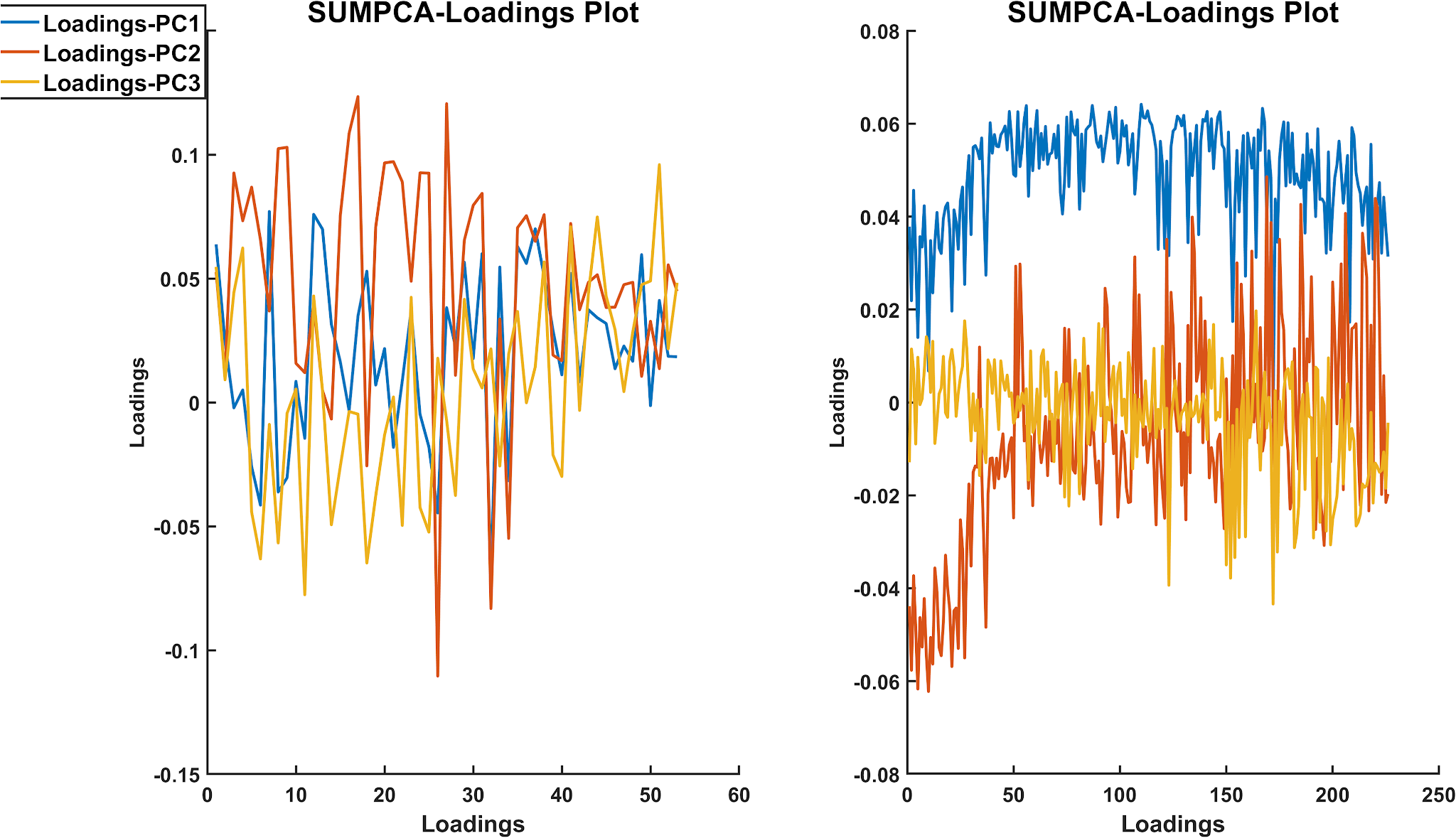

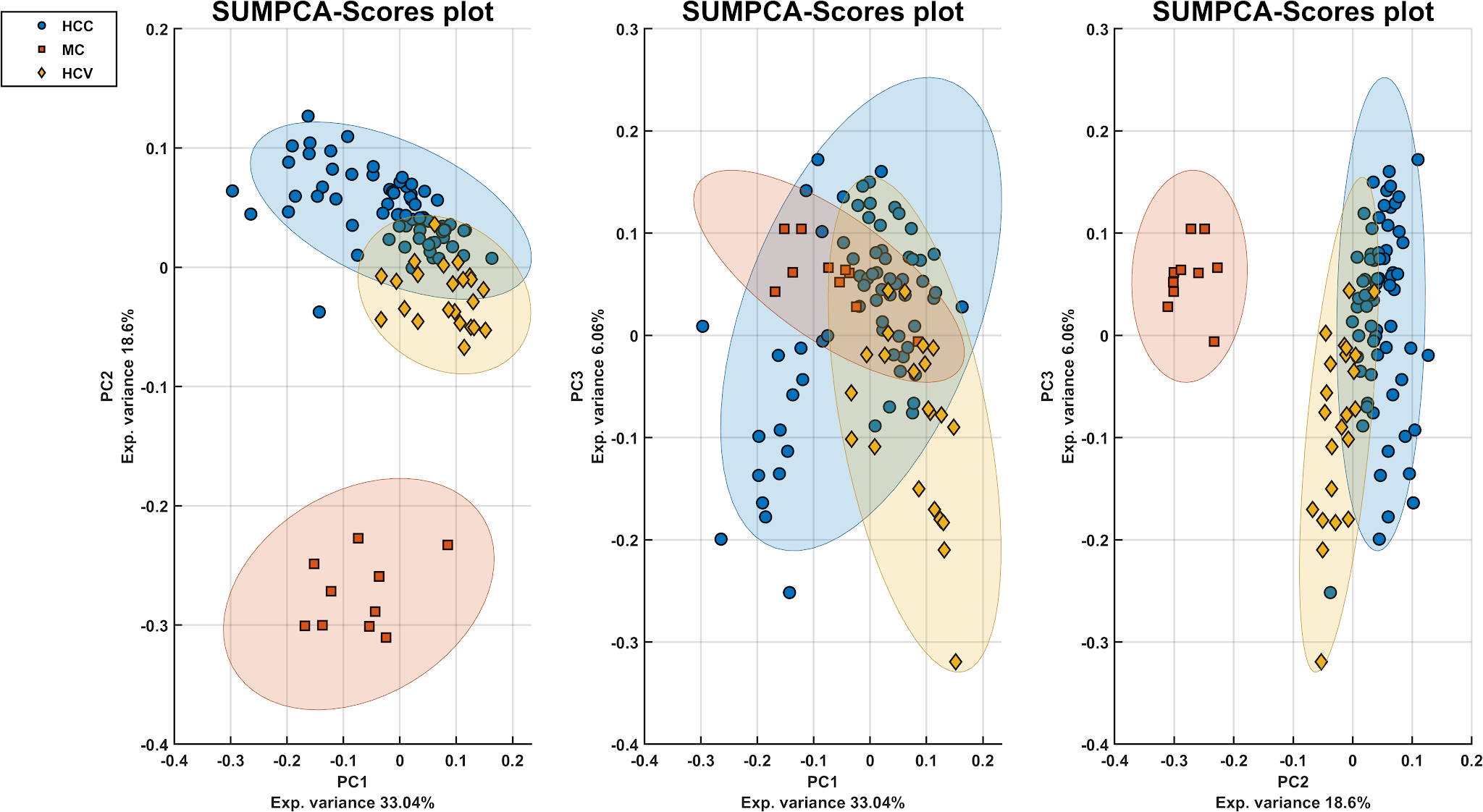


**Figure S5.** SUMPCA of lipidomics and metabolomics datasets, the panel reports the bi-dimensional super scores (T^sup^) plot for PC1, PC2 and PC3. The datasets were pre-processed independently for the two modalities: 1) normalized 2) the missing values and zeros were replaced with one-fifth of the minimum value recorded in the data set for that molecule 3) logarithm values of the base of 10 and 4) autoscaling.

**
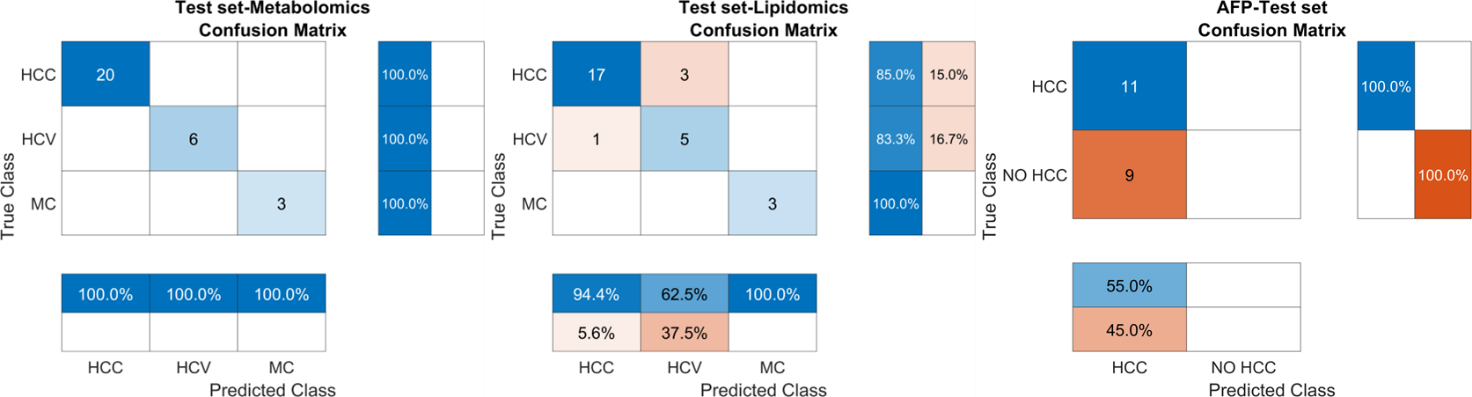
**

**Figure S6.** Graphical representation of confusion matrices obtained from PLS-DA models of both independent modality . The reported confusion matrices refer to PLS-DA performance for the test phase.


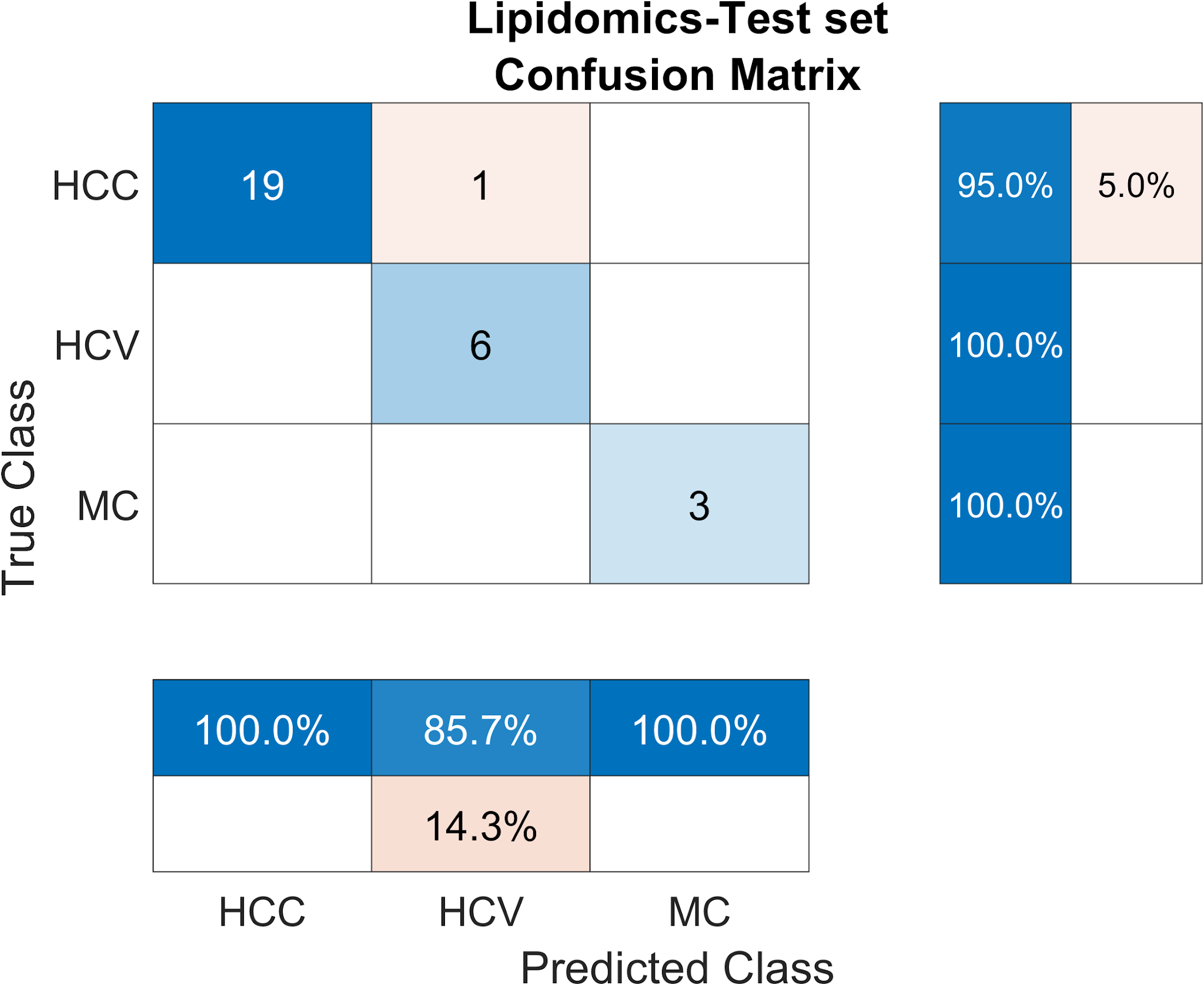

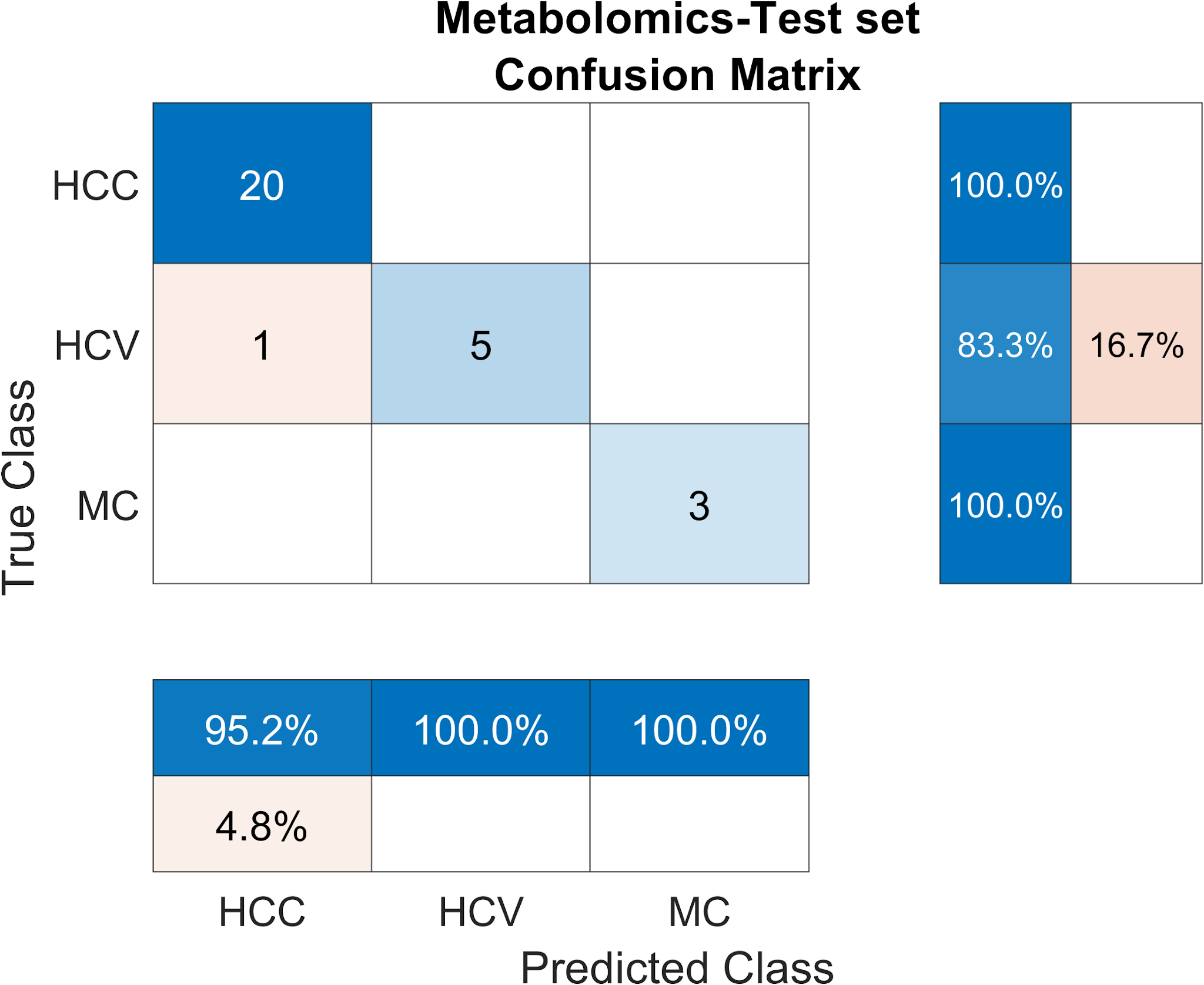


**Figure S7.** Graphical representation of confusion matrices obtained from SIMCA models of both independent modalities. The reported confusion matrices refer to SIMCA performance for the test phase.


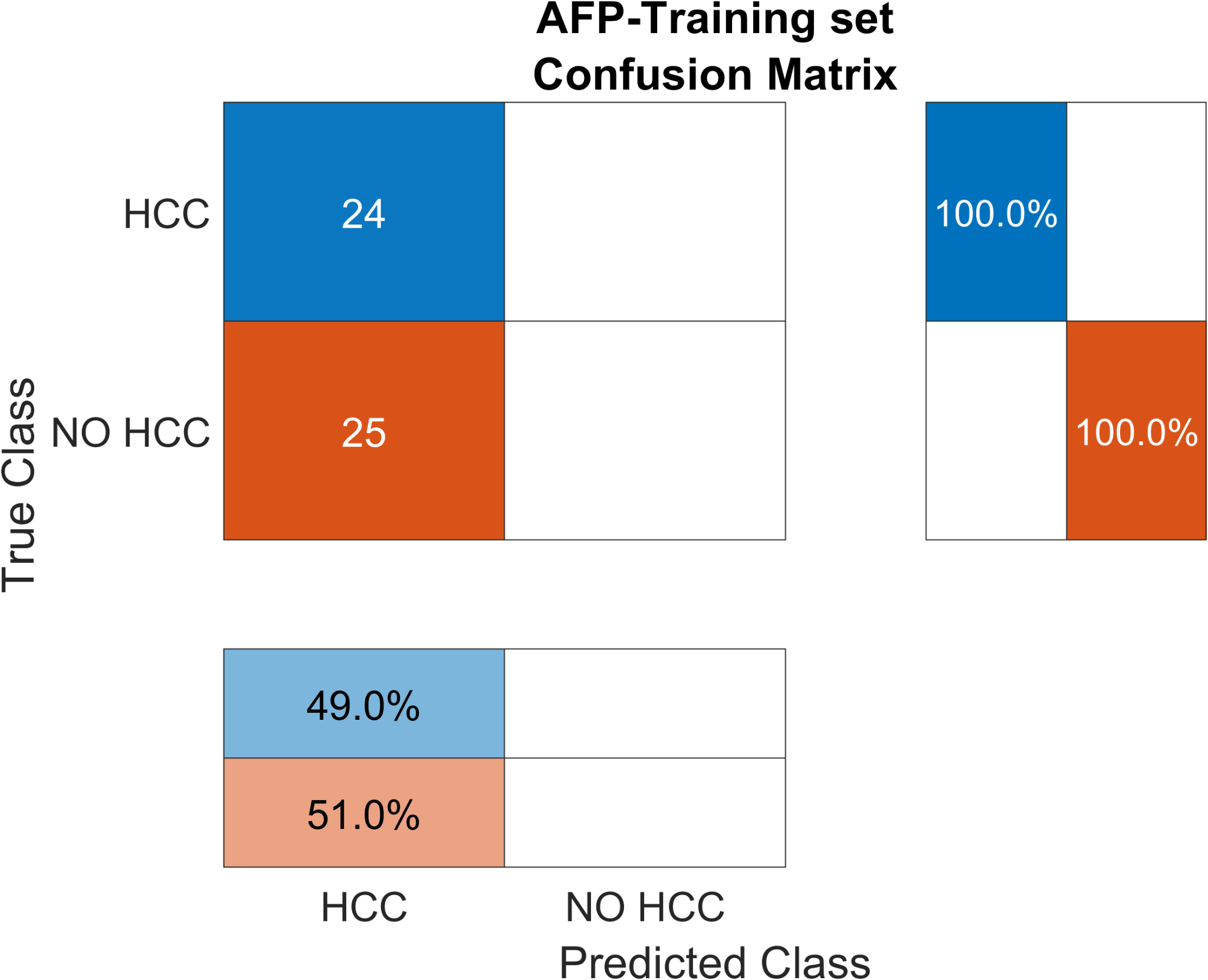

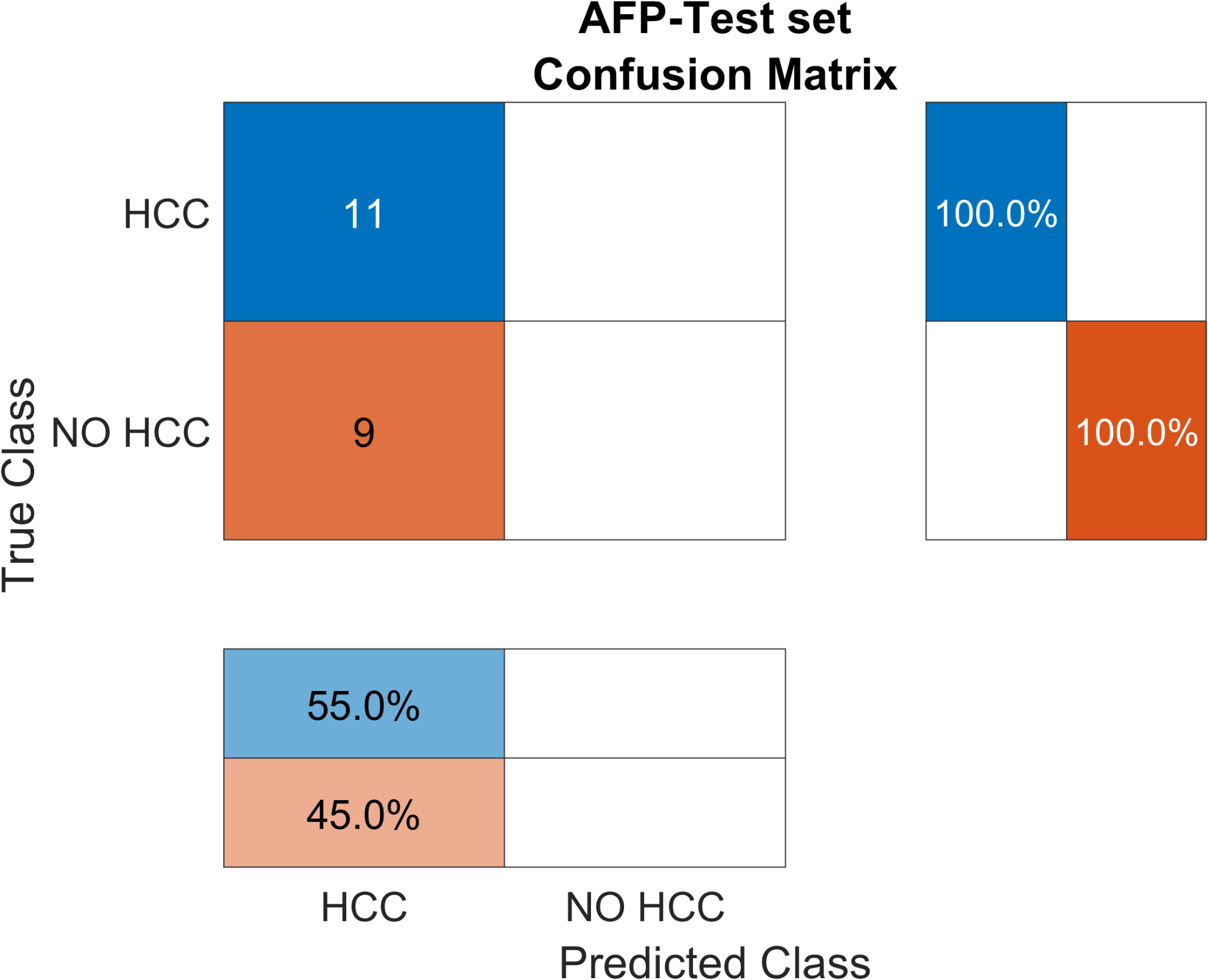


**Figure S8.** Graphical representation of confusion matrices obtained for AFP classification.

**Additional section S3: Lipid and metabolite annotation**

**Section S3.1: Lipid annotation**

In this section some examples of lipid annotation workflow are proposed:

Sphingomyelins: 35 SMs were annotated, all of them were detected in positive mode and in the protonated form ([M+H]^+^), as indicated by <https://lipidomicstandards.org/lipid-class-specific-fragments/>.

Few SMs (such as: SM 18:1;2O/24:1, SM 18:1;2O/22:0 etc) were detected also in [M+Na]^+^, [M+K]^+^ forms, but these were not the primary adducts or the adducts used to normalize them, as indicated in the caption of **Table S2**.

We annotated, at the end, 13 SMs (**figure S9**) with 1 Double Bond (DB) and we checked that all of them followed the equivalent carbon number theory and that CCS values increased linearly with measured m/z. This strategy is useful to easily identify outliers, indeed at the beginning our dataset included 14 SMs (upper panel) with 1 DB and we discarded the SM with a total of 42 carbons eluting at 2.48 min (red circle). In this way our R^2^ improved from 0.9925 (upper panel) to 0.999 (lower panel), indicating a strong linearity and the respect of ECN model.


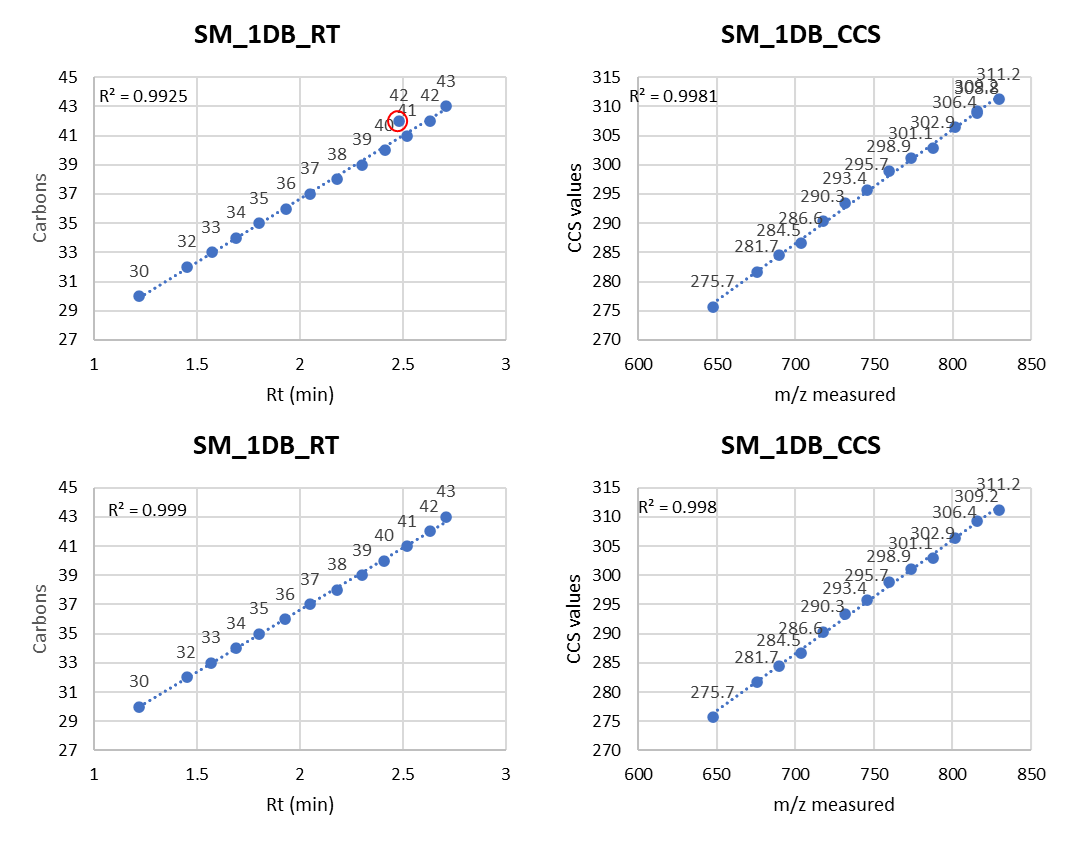


**Figure S9.** Sphingomyelins rt/carbon number and m/z measured/CCS linearity.

We here report some examples that we followed for short/long hand SMs’ annotations.

If the MS^2^ spectrum contained only the phosphocoline head group (m/z 184) we annotated the lipid in its short-hand form, as for the SM 36:2;2O **figure S10**.


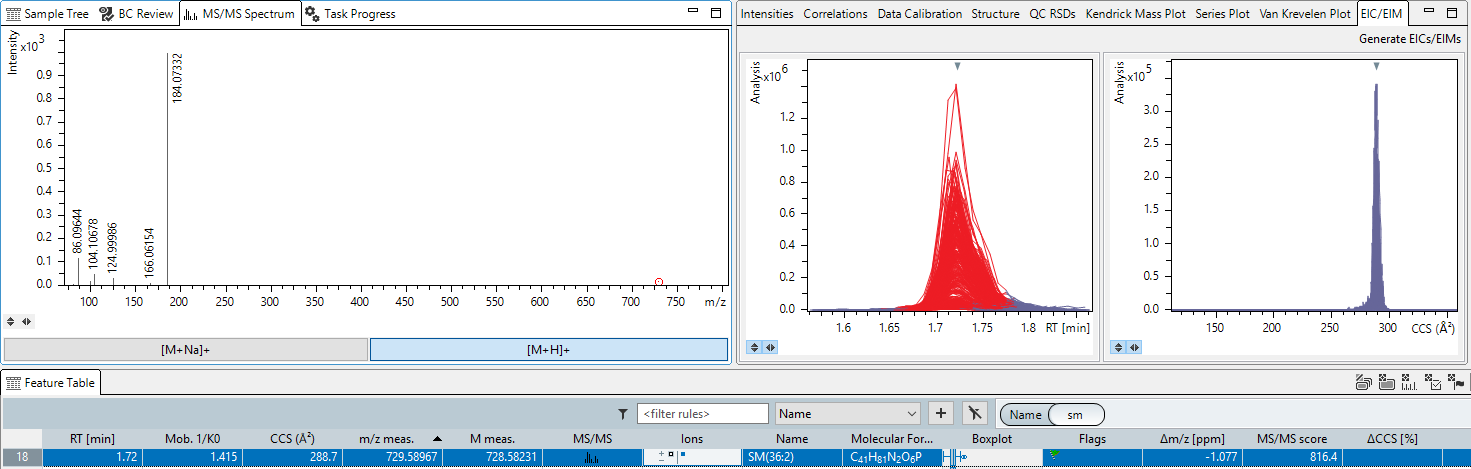


**Figure S10.** Short-hand annotation for SMs.

On the other hand, if our spectra contained additional fragments related to the lipid backbone we proposed it in the long-hand form, as for the SM 18:1;2O/22:0 where we observed a fragment at m/z 264.26932 due to LCB 18:1;2(-H_3_O_2_) **figure S11**.


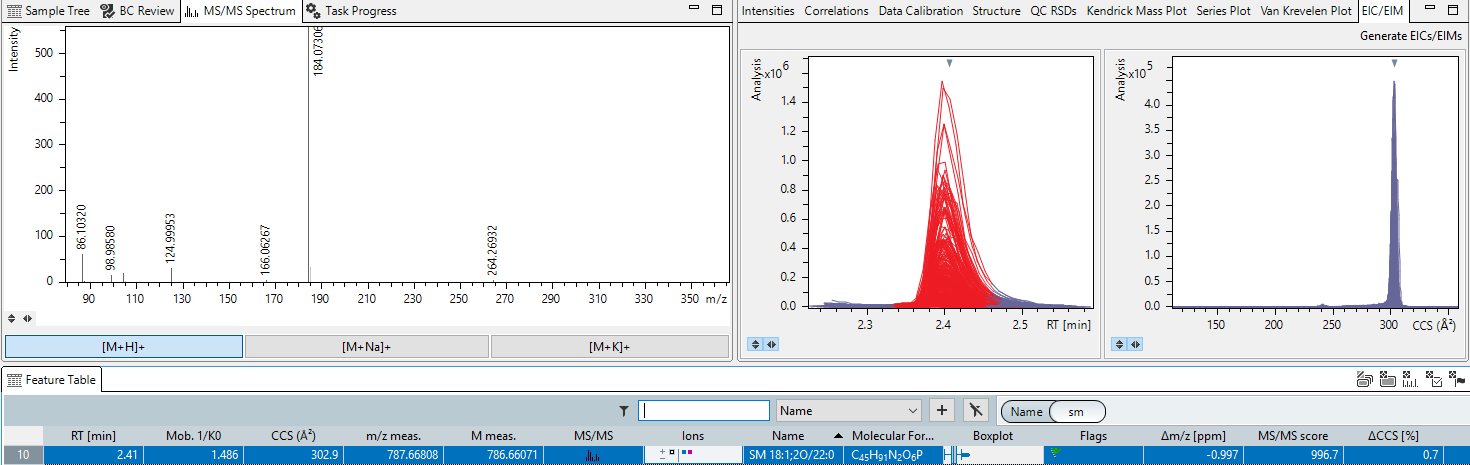


**Figure S11.** Long-hand annotation for SMs.

Below we report the order of elution and the CCS values of phosphocolines with 0, 1, 2 and 3 DBs in ESI^+^, plotting in this way 29 out of the 64 PCs (considering both polarities) reported at the end (**figure S12).**


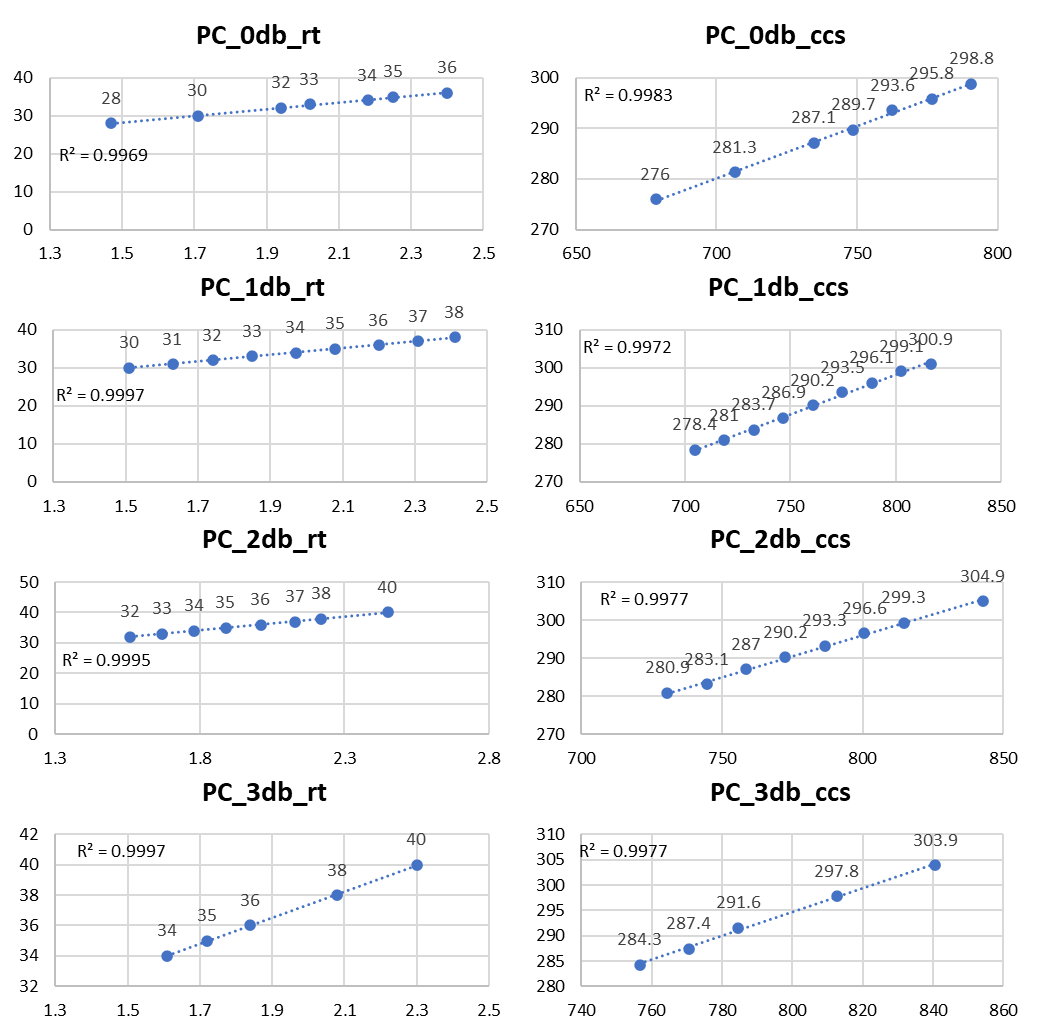


**Figure S12.** Phosphatidylcholines rt/carbon number and m/z measured /CCS linearity.

Also for PCs we inspected the presence of fragments related to the acyl chains composing the lipid. In the proposed case, PC 18:0_20:4, in addition to the m/z 184.07332 we have additional fragments at m/z 506.35904 and 524.36600 due to the NL of 20:4 chain and at m/z 544.33252 and 526.32088 conducible to the NL of 18:0 chain **figure S13**.


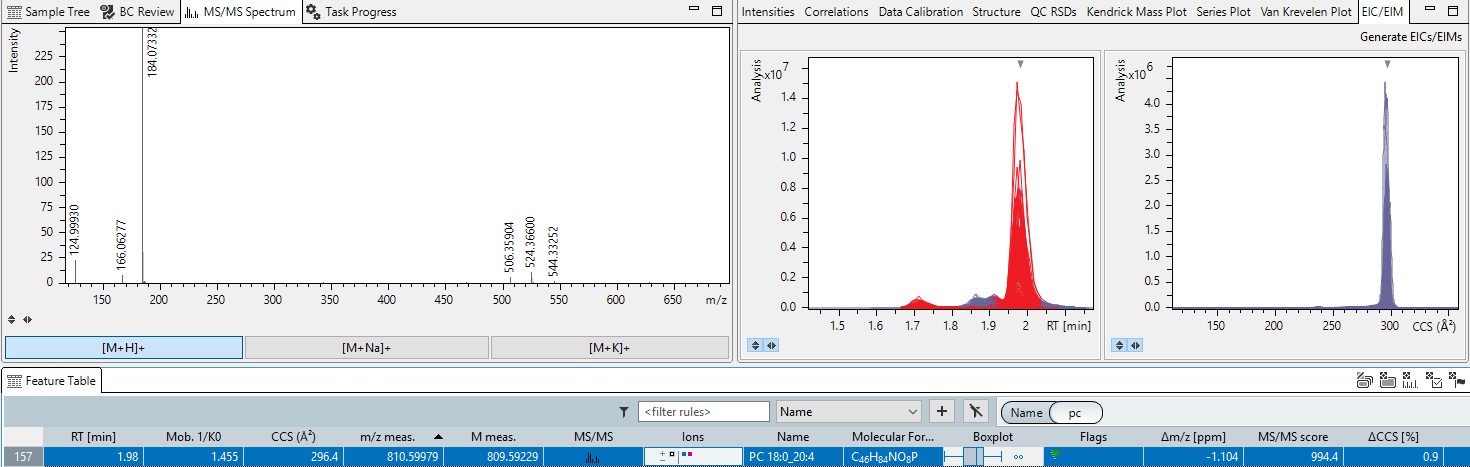


**Figure S13** **.** Long-hand annotation for PCs.

PCs have been listed in the short-hand form if the MS^2^ reported only the phosphocholine head group (m/z 184) in positive ionization mode.

Finally we report some MS/MS library matches for LPC O-16:0, LPC 18:1, CAR 4:0, and ADMA.

In the MS2 spectrum of the LPC 18:1 and LPC O-16:0 are well present fragments at m/z 86.099, 184.074 and 104.108 due respectively to: deydrocholine, phosphocoline and choline.

CAR is characterized by the fragment at 85 m/z (C_4_H_5_O_2_^+^) which is the most significant feature peak of acylcarnitine spectra. For ADMA it can be observed the characteristic fragmentation pattern of Arg, with ions at 70, 116 m/z, plus the ion at 158 m/z, which fits with reported fragmentation pattern and CCS (<https://pubchem.ncbi.nlm.nih.gov/compound/N_N-dimethylarginine#section=Other-MS>) even if the fragment at m/z 46 was not detected for the MS range employed (50-1500)


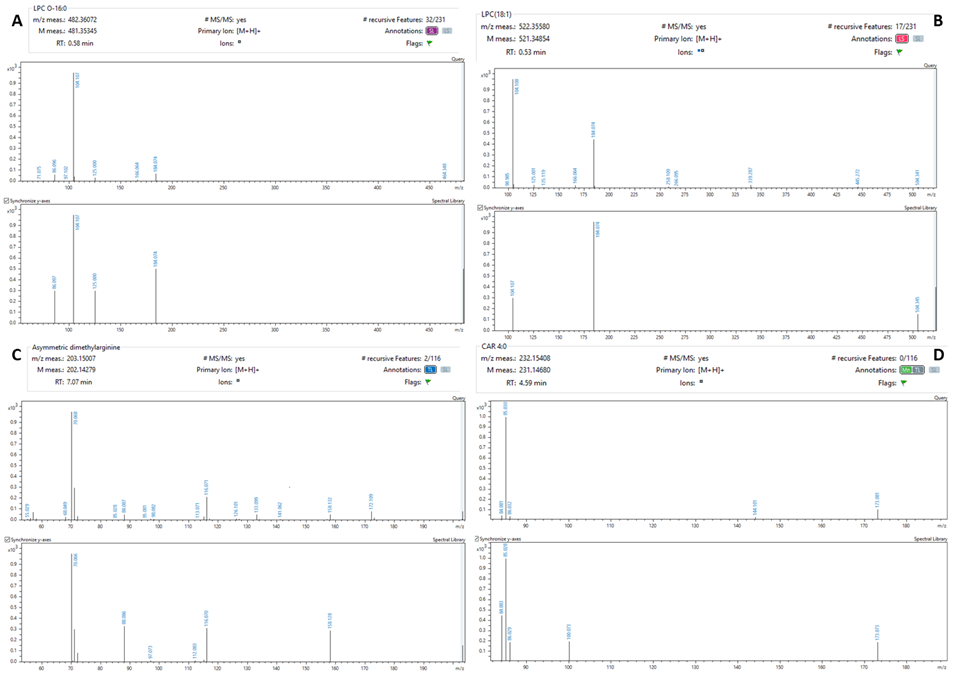


**Figure S14.** Comparison between the acquired (Query) and the library spectrum (Spectral library) of a statistically significant lipids and metabolites (A: LPC O-16:0 ; B: LPC 18:1; C: Asymmetric dimethylarginine; D: CAR 4:0 ).

**Table S1.** List of annotated metabolites by HILIC-UHPLC-TIMS-Q-TOF in HCC, HCV and MC plasma samples. Metabolites annotation was carried out with a spectral library approach following the metabolomics standard initiative guidelines^3^. Abbreviations: RT, retention time; m/z meas, mass to charge measured; Mass meas., measured mass; CCS, cross collisional section; Mob., mobility. Primary ion has been used for quantification and additional adducts, if detected, were reported in round brackets.

| **RT [min]** | **CCS (Å²)** | **m/z meas.** | **M meas.** | **Mob. 1/K0** | **Ions** | **Name** | **Molecular Formula** | **MS/MS score** | **Δm/z [ppm]** | **ΔCCS [%]** |
| --- | --- | --- | --- | --- | --- | --- | --- | --- | --- | --- |
| 5.7 | 133.7 | 160.13322 | 159.12594 | 0.616 | [M+H]^+^ | Aminooctanoic acid | C_8_H_17_NO_2_ | 560.3 | 0.057 | 3.0 |
| 1.18 | 195.8 | 369.17463 | 370.1819 | 0.942 | [M-H]^-^ | Androsterone sulfate | C_19_H_30_O_5_S | 899.1 | -0.041 | 2.9 |
| 1.27 | 183.5 | 303.23318 | 304.24046 | 0.877 | [M-H]^-^ | Arachidonic acid | C_20_H_32_O_2_ | 830.1 | 1.096 | 3.0 |
| 7.66 | 135.8 | 175.11864 | 174.11137 | 0.63 | [M+H]^+^ | Arginine | C_6_H_14_N_4_O_2_ | 986.7 | -1.437 | 1.7 |
| 7.07 | 143.6 | 203.15007 | 202.14279 | 0.672 | [M+H]^+^ | Asymmetric dimethylarginine | C_8_H_18_N_4_O_2_ | 872.1 | -0.91 | 2.7 |
| 1.13 | 240.4 | 585.27057 | 584.26329 | 1.173 | [M+H]^+^ | Bilirubin | C_33_H_36_N_4_O_6_ | 901.1 | -0.352 | 0.6 |
| 2.51 | 242.4 | 583.25559 | 582.24831 | 1.182 | [M+H]^+^ | Biliverdin | C_33_H_34_N_4_O_6_ | 598.2 | 0.795 | 1.0 |
| 3.76 | 190.5 | 316.24831 | 315.24103 | 0.912 | [M+H]^+^ | CAR 10:0 | C_17_H_33_NO_4_ | 800.3 | 0.102 | 3.0 |
| 3.8 | 184.7 | 314.23275 | 313.22547 | 0.884 | [M+H]^+^ | CAR 10:1 | C_17_H_31_NO_4_ | 960.3 | 0.439 | 1.5 |
| 3.63 | 204.2 | 370.29552 | 369.28964 | 0.983 | [M+H]^+^, ([M+Na]^+^) | CAR 14:1 | C_21_H_39_NO_4_ | 900.3 | 0.9 | 1.1 |
| 3.59 | 211.5 | 398.32699 | 397.31971 | 1.021 | [M+H]^+^ | CAR 16:1_A | C_23_H_43_NO_4_ | 970.5 | 0.846 | 0.1 |
| 3.47 | 211.2 | 398.32648 | 397.3192 | 1.02 | [M+H]^+^ | CAR 16:1_B | C_23_H_43_NO_4_ | 990.1 | -0.123 | 0.2 |
| 3.55 | 206 | 396.31189 | 395.30462 | 0.994 | [M+H]^+^ | CAR 16:2 | C_23_H_41_NO_4_ | 877.7 | 3.347 | 2.4 |
| 3.39 | 219 | 426.35783 | 425.35055 | 1.059 | [M+H]^+^ | CAR 18:1_A | C_25_H_47_NO_4_ | 899.1 | 0.208 | 1.1 |
| 3.54 | 219.1 | 426.35883 | 425.35156 | 1.06 | [M+H]^+^ | CAR 18:1_B | C_25_H_47_NO_4_ | 943.5 | 3.205 | 1.0 |
| 5.5 | 143.4 | 204.12322 | 203.11594 | 0.671 | [M+H]^+^ | CAR 2:0 | C_9_H_17_NO_4_ | 871.8 | 1.064 | 0.7 |
| 5.07 | 148.3 | 218.1381 | 217.13082 | 0.697 | [M+H]^+^ | CAR 3:0 | C_10_H_19_NO_4_ | 967.3 | -1.874 | 0.5 |
| 4.59 | 154.2 | 232.15408 | 231.1468 | 0.728 | [M+H]^+^ | CAR 4:0 | C_11_H_21_NO_4_ | 921.6 | -0.834 | 1.6 |
| 4.31 | 159.6 | 246.16982 | 245.16255 | 0.755 | [M+H]^+^ | CAR 5:0 | C_12_H_23_NO_4_ | 996.5 | -0.58 | 1.6 |
| 5.84 | 161 | 276.14418 | 275.1369 | 0.766 | [M+H]^+^ | CAR 5:1;O2 | C_12_H_21_NO_6_ | 820.7 | 0.046 | 0.1 |
| 4.48 | 159 | 244.15388 | 243.1466 | 0.752 | [M+H]^+^ | CAR 5:1 | C_12_H_21_NO_4_ | 910.5 | -0.086 | 2.3 |
| 4.1 | 166.9 | 260.18546 | 259.17819 | 0.792 | [M+H]^+^ | CAR 6:0 | C_13_H_25_NO_4_ | 655.9 | -0.425 | 2.2 |
| 3.89 | 179.1 | 288.21724 | 287.20996 | 0.854 | [M+H]^+^ | CAR 8:0 | C_15_H_29_NO_4_ | 765.3 | 1.015 | 3.0 |
| 3.97 | 175 | 286.20138 | 285.1941 | 0.834 | [M+H]^+^ | CAR 8:1 | C_15_H_27_NO_4_ | 987.6 | 0.608 | 1.9 |
| 3.8 | 181.3 | 302.23251 | 301.22524 | 0.866 | [M+H]^+^ | CAR 9:0 | C_16_H_31_NO_4_ | 897.8 | -0.444 | 2.6 |
| 5.9 | 132.4 | 162.11254 | 161.10526 | 0.61 | [M+H]^+^ | Carnitine | C_7_H_15_NO_3_ | 588.1 | 0.485 | 0.0 |
| 1 | 135.1 | 187.00719 | 188.01446 | 0.629 | [M-H]^-^ | Cresol sulfate | C_7_H_8_O_4_S | 943.9 | 0.997 | 1.7 |
| 1.15 | 196.2 | 367.15889 | 368.16616 | 0.944 | [M-H]^-^ | Dehydroepiandrosterone sulfate | C_19_H_28_O_5_S | 999.8 | 0.944 | 4.7 |
| 4.25 | 200.5 | 448.30692 | 449.3142 | 0.972 | [M-H]^-^ | Deoxycholic acid glycine conjugate | C_26_H_43_NO_5_ | 998.8 | -0.019 | 2.8 |
| 3.7 | 194.9 | 342.26504 | 341.25777 | 0.936 | [M+H]^+^ | Dodecenoylcarnitine | C_19_H_35_NO_4_ | 822.1 | 2.424 | 1.7 |
| 1.49 | 179.6 | 295.22836 | 296.23564 | 0.857 | [M-H]^-^ | FA 18:1+1O | C_18_H_32_O_3_ | 869.6 | 2.213 | 0.2 |
| 6.5 | 156.1 | 258.11014 | 257.10286 | 0.74 | [M+H]^+^ | Glycerophosphocholine | C_8_H_20_NO_6_P | 992.1 | 0.151 | 0.1 |
| 7.61 | 130.7 | 156.07681 | 155.06953 | 0.601 | [M+H]^+^ | Histidine | C_6_H_9_N_3_O_2_ | 899.2 | 0.359 | 0.8 |
| 7.53 | 140.1 | 189.13533 | 188.12805 | 0.653 | [M+H]^+^ | Homoarginine | C_7_H_16_N_4_O_2_ | 920.6 | 2.934 | 1.5 |
| 1.3 | 180.9 | 313.23727 | 312.22999 | 0.866 | [M+H]^+^ | Hydroperoxylinoleic acid | C_18_H_32_O_4_ | 606.7 | -0.114 | 1.5 |
| 2.46 | 124.4 | 154.05006 | 123.04278 | 0.571 | [M+H]^+^ | Hydroxyanthranilic acid | C_7_H_7_NO_3_ | 976.2 | 1.85 | 3.0 |
| 1.27 | 207.1 | 411.18539 | 412.19266 | 1.001 | [M-H]^-^ | Hydroxypregnenolone sulfate | C_21_H_32_O_6_S | 619.9 | 0.84 | 3.0 |
| 1.14 | 128.7 | 158.06121 | 159.06849 | 0.592 | [M-H]^-^ | Indole-acetaldehyde | C_10_H_9_NO | 609.8 | 0.524 | 4.2 |
| 1.18 | 142.2 | 212.00253 | 213.00981 | 0.668 | [M-H]^-^ | Indoxyl sulfate | C_8_H_7_NO_4_S | 752.0 | 1.094 | 0.2 |
| 5.93 | 149.3 | 229.15457 | 228.14729 | 0.704 | [M+H]^+^ | Isoleucylproline | C_11_H_20_N_2_O_3_ | 500.2 | -0.481 | 2.5 |
| 4.19 | 130.2 | 166.07248 | 165.06521 | 0.601 | [M+H]^+^ | Methylguanine | C_6_H_7_N_5_O | 622.1 | 0.912 | 1.8 |
| 7.59 | 133.2 | 170.09257 | 169.08529 | 0.616 | [M+H]^+^ | Methylhistidine | C_7_H_11_N_3_O_2_ | 849.0 | 0.81 | 1.7 |
| 2.29 | 130.3 | 153.06585 | 152.05858 | 0.598 | [M+H]^+^ | Methylpyridonecarboxamide | C_7_H_8_N_2_O_2_ | 899.1 | -0.107 | 1.2 |
| 1.77 | 133 | 181.07201 | 180.06473 | 0.618 | [M+H]^+^ | Paraxanthine | C_7_H_8_N_4_O_2_ | 918.5 | -0.371 | 1.1 |
| 4.42 | 159.2 | 265.11828 | 264.111 | 0.756 | [M+H]^+^ | Phenylacetylglutamine | C_13_H_16_N_2_O_4_ | 677.9 | -0.297 | 0.1 |
| 1.11 | 205.1 | 395.18855 | 396.19582 | 0.99 | [M-H]^-^ | Pregnenolone sulfate | C_21_H_32_O_5_S | 711.7 | -1.376 | 3.0 |
| 5.31 | 126.2 | 144.10192 | 143.09485 | 0.577 | [M+H]^+^, ([M+Na]^+^, [M+K]^+^) | Proline betaine | C_7_H_13_NO_2_ | 959.9 | 0.779 | 0.2 |
| 6.59 | 151.2 | 229.11857 | 228.11129 | 0.713 | [M+H]^+^ | Proline-hydroxyproline | C_10_H_16_N_2_O_4_ | 987.1 | 0.864 | 1.3 |
| 5.02 | 146.9 | 243.06252 | 244.0698 | 0.695 | [M-H]^-^ | Pseudouridine | C_9_H_12_N_2_O_6_ | 936.5 | 0.93 | 3.2 |
| 4.54 | 203.4 | 380.25458 | 379.2473 | 0.98 | [M+H]^+^ | Sphingosine 1-phosphate | C_18_H_38_NO_5_P | 980.3 | -2.402 | 0.9 |
| 5.67 | 115.9 | 124.00733 | 125.0146 | 0.523 | [M-H]^-^ | Taurine | C_2_H_7_NO_3_S | 998.1 | -0.481 | 3.0 |
| 5.68 | 142.3 | 180.06673 | 181.07401 | 0.661 | [M-H]^-^ | Tyrosine | C_9_H_11_NO_3_ | 908.2 | 0.621 | 2.5 |
| 4.22 | 230.9 | 591.31872 | 590.31144 | 1.127 | [M+H]^+^ | Urobilinogen | C_33_H_42_N_4_O_6_ | 721.2 | 1.084 | 3.0 |

**Table S2.** List of annotated lipids by RP-UHPLC-TIMS-Q-TOF in HCC, HCV and MC plasma samples. Lipids are sorted by class. Lipid annotation was carried out with a combined rule based and spectral library approach following the lipidomic standard initiative guidelines^4^. Abbreviations: RT, retention time; m/z meas, mass to charge measured; Mass meas., measured mass; CCS, cross collisional section; Mob., mobility. Primary ion has been used for normalization and additional adducts, if detected, were reported in round brackets. Lipid class abbreviations: cholesteryl esters (CEs), ceramides (Cers), diacylglycerols (DGs), dihexosylceramide (Hex2cer), hexosylceramides (Hexcers), lysophosphatidylcholines (LPCs), ether-linked lysophosphatidylcholines (LPC-Os), lysophosphatidylethanolamines (LPEs), phosphatidylcholines (PCs), ether-linked phosphatidylcholine (PC-Os) phosphatidylethanolamines (PEs), ether-linked phosphatidylethanolamine (PE-Os), phosphatidylinositols (PIs), sphingomyelins (SMs), triacylglycerols (TGs).

| **RT [min]** | **CCS (Å²)** | **m/z meas.** | **M meas.** | **Mob. 1/K0** | **Ions** | **Name** | **Molecular Formula** | **MS/MS score** | **Δm/z [ppm]** | **ΔCCS [%]** |
| --- | --- | --- | --- | --- | --- | --- | --- | --- | --- | --- |
| 3.32 | 284.2 | 642.6174 | 624.58385 | 1.389 | [M+NH_4_]^+^, ([M+Na]^+^, [M+K]^+^) | CE 16:0 | C_43_H_76_O_2_ | 872.1 | -1.436 | 1.8 |
| 3.24 | 283.5 | 640.60243 | 622.56861 | 1.386 | [M+NH_4_]^+^ | CE 16:1 | C_43_H_74_O_2_ | 803.1 | -0.43 | 2.5 |
| 3.31 | 289.2 | 668.63442 | 650.60014 | 1.415 | [M+NH_4_]^+^, ([M+H]^+^) | CE 18:1 | C_45_H_78_O_2_ | 820.7 | 0.616 | 2.1 |
| 3.25 | 289.5 | 666.61862 | 648.58479 | 1.416 | [M+NH_4_]^+^ | CE 18:2 | C_45_H_76_O_2_ | 760.7 | 0.392 | 2.6 |
| 3.26 | 294.4 | 692.63127 | 674.59744 | 1.441 | [M+NH_4_]^+^ | CE 20:3 | C_47_H_78_O_2_ | 931 | -3.881 | 3.1 |
| 3.21 | 292.9 | 690.61797 | 672.58319 | 1.434 | [M+NH_4_]^+^, ([M+H]^+^, [M+K]^+^, [M+Na]^+^) | CE 20:4 | C_47_H_76_O_2_ | 735.3 | -0.215 | 2.5 |
| 3.15 | 290.7 | 688.60212 | 670.56818 | 1.423 | [M+NH_4_]^+^, ([M+K]^+^, [M+Na]^+^ ) | CE 20:5 | C_47_H_74_O_2_ | 784.1 | -0.786 | 3.6 |
| 2.48 | 271.1 | 594.58198 | 593.5747 | 1.323 | [M+H]^+^ | Cer 16:1;2O/22:0 | C_38_H_75_NO_3_ | 928.6 | 0.011 | 2.1 |
| 2.7 | 277 | 622.61327 | 621.60617 | 1.353 | [M+H]^+^, ([M+K]^+^, [M+Na]^+^, [M+H-H_2_O]^+^) | Cer 18:1;2O/22:0 | C_40_H_79_NO_3_ | 932.7 | -0.222 | 1.4 |
| 2.77 | 281.1 | 636.62848 | 635.62096 | 1.374 | [M+H]^+^, ([M+H-H_2_O]^+^, [M+Na]^+^) | Cer 18:1;2O/23:0 | C_41_H_81_NO_3_ | 967.8 | -0.667 | 2.7 |
| 2.85 | 283 | 650.64423 | 649.63698 | 1.384 | [M+H]^+^, ([M+H-H_2_O]^+^) | Cer 18:1;2O/24:0 | C_42_H_83_NO_3_ | 969.6 | -0.527 | 2.3 |
| 2.69 | 279.8 | 648.62832 | 647.62105 | 1.368 | [M+H]^+^ | Cer 18:1;2O/24:1 | C_42_H_81_NO_3_ | 966.8 | -0.787 | 1.7 |
| 2.5 | 272.9 | 620.59698 | 619.5897 | 1.333 | [M+H]^+^ | Cer 18:2;2O/22:0 | C_40_H_77_NO_3_ | 974.1 | -1.036 | 0.7 |
| 2.62 | 276.2 | 634.61303 | 633.60575 | 1.35 | [M+H]^+^ | Cer 18:2;2O/23:0 | C_41_H_79_NO_3_ | 968.9 | -0.151 | 1 |
| 2.51 | 276.4 | 646.61191 | 645.60463 | 1.351 | [M+H]^+^ | Cer 18:2;2O/24:1 | C_42_H_79_NO_3_ | 918.1 | -2.111 | 1.4 |
| 2.45 | 265.8 | 612.55579 | 594.52223 | 1.298 | [M+NH_4_]^+^, ([M+H-H_2_O]^+^, [M+H]^+^, [M+Na]^+^, [M+K]^+^) | DG 16:0_18:1 | C_37_H_70_O_5_ | 939.4 | -0.612 | 1.3 |
| 2.67 | 271.4 | 640.58702 | 622.5532 | 1.327 | [M+NH_4_]^+^, ([M+Na]^+^, [M+H-H_2_O]^+^, [M+K]^+^) | DG 18:0_18:1 | C_39_H_74_O_5_ | 941.3 | -0.651 | 2.4 |
| 2.47 | 268.3 | 638.57128 | 620.53739 | 1.311 | [M+NH_4_]^+^, ([M+H]^+^, [M+Na]^+^, [M+H-H_2_O]^+^, [M+K]^+^) | DG 18:1_18:1 | C_39_H_72_O_5_ | 968.5 | -0.824 | 2.4 |
| 2.29 | 264.4 | 636.55552 | 618.52167 | 1.292 | [M+NH_4_]^+^, ([M+K]^+^, [M+Na]^+^, [M+H-H_2_O]^+^) | DG 18:1_18:2 | C_39_H_70_O_5_ | 934.8 | -0.997 | 1.8 |
| 1.68 | 306.3 | 862.62433 | 861.61705 | 1.505 | [M+H]^+^ | Hex2Cer 18:1;2O/16:0 | C_46_H_87_NO_13_ | 499.6 | -0.837 | 2.1 |
| 2.68 | 305.2 | 812.69395 | 811.68787 | 1.499 | [M+H]^+^, ([M+Na]^+^) | HexCer 18:1;2O/24:0 | C_48_H_93_NO_8_ | 408.2 | -4.13 | 1.3 |
| 0.4 | 225.4 | 468.30896 | 467.30169 | 1.093 | [M+H]^+^ | LPC 14:0 | C_22_H_46_NO_7_P | 584.5 | 1.053 | 0.4 |
| 0.45 | 228.6 | 482.32461 | 481.31733 | 1.11 | [M+H]^+^ | LPC 15:0 | C_23_H_48_NO_7_P | 715.8 | 1.016 | 0.5 |
| 0.51 | 232.7 | 496.33988 | 495.33261 | 1.131 | [M+H]^+^ | LPC 16:0 | C_24_H_50_NO_7_P | 717.5 | 0.237 | 1.9 |
| 0.42 | 227.7 | 494.32466 | 493.31721 | 1.106 | [M+H]^+^, ([M+Na]^+^) | LPC 16:1 | C_24_H_48_NO_7_P | 632.8 | 1.101 | 0.5 |
| 0.57 | 236.9 | 510.35577 | 509.34849 | 1.152 | [M+H]^+^ | LPC 17:0 | C_25_H_52_NO_7_P | 644.4 | 0.685 | 0.8 |
| 0.47 | 231.5 | 508.33989 | 507.33262 | 1.126 | [M+H]^+^ | LPC 17:1 | C_25_H_50_NO_7_P | 446.1 | 0.249 | 1.8 |
| 0.67 | 240.5 | 524.37124 | 523.36397 | 1.17 | [M+H]^+^ | LPC 18:0 | C_26_H_54_NO_7_P | 713.5 | 0.34 | 0.8 |
| 0.53 | 235.2 | 522.3558 | 521.34854 | 1.144 | [M+H]^+^, ([M+K]^+^, [M+Na]^+^) | LPC 18:1_A | C_26_H_52_NO_7_P | 643.2 | 0.747 | 0.1 |
| 0.53 | 241.8 | 566.34631 | 521.34811 | 1.179 | [M+HCOO]^-^ | LPC 18:1_B | C_26_H_52_NO_7_P | 944.5 | -0.047 | 0.6 |
| 0.45 | 229.8 | 520.34014 | 519.33287 | 1.118 | [M+H]^+^ | LPC 18:2_A | C_26_H_50_NO_7_P | 703.7 | 0.747 | 1.1 |
| 0.45 | 238.9 | 564.33085 | 519.33264 | 1.164 | [M+HCOO]^-^ | LPC 18:2_B | C_26_H_50_NO_7_P | 946.2 | 0.262 | 2.1 |
| 0.41 | 226.9 | 518.32392 | 517.31664 | 1.104 | [M+H]^+^ | LPC 18:3 | C_26_H_48_NO_7_P | 623.9 | -0.13 | 0.5 |
| 0.78 | 244.3 | 538.38687 | 537.37959 | 1.189 | [M+H]^+^ | LPC 19:0 | C_27_H_56_NO_7_P | 783.3 | 0.223 | 0.8 |
| 0.93 | 247.4 | 552.40238 | 551.3951 | 1.205 | [M+H]^+^ | LPC 20:0 | C_28_H_58_NO_7_P | 665.5 | 0.113 | 0.8 |
| 0.56 | 237.5 | 548.37036 | 547.36308 | 1.157 | [M+H]^+^ | LPC 20:2 | C_28_H_54_NO_7_P | 666.3 | -1.285 | 0.5 |
| 0.47 | 234.2 | 546.355 | 545.34773 | 1.14 | [M+H]^+^ | LPC 20:3 | C_28_H_52_NO_7_P | 669.3 | -0.783 | 0.1 |
| 0.44 | 232.9 | 544.33999 | 543.33258 | 1.134 | [M+H]^+^, ([M+Na]^+^) | LPC 20:4_A | C_28_H_50_NO_7_P | 617.9 | 0.404 | 0.5 |
| 0.44 | 242.9 | 588.33088 | 543.33268 | 1.185 | [M+HCOO]^-^ | LPC 20:4_B | C_28_H_50_NO_7_P | 933.8 | 0.387 | 1.5 |
| 0.42 | 235.5 | 568.33965 | 567.33237 | 1.148 | [M+H]^+^ | LPC 22:6 | C_30_H_50_NO_7_P | 690.6 | -0.184 | 0.5 |
| 0.58 | 234.1 | 482.36072 | 481.3535 | 1.137 | [M+H]^+^, ([M+Na]^+^) | LPC O-16:0 | C_24_H_52_NO_6_P | 880.7 | 0.468 | 1 |
| 0.56 | 228.7 | 480.34516 | 479.33789 | 1.11 | [M+H]^+^ | LPC O-16:1 | C_24_H_50_NO_6_P | 940.5 | 0.897 | 0.6 |
| 0.8 | 241.4 | 510.39204 | 509.38476 | 1.174 | [M+H]^+^ | LPC O-18:0 | C_26_H_56_NO_6_P | 884.6 | 0.5 | 0.4 |
| 0.6 | 236.7 | 508.37644 | 507.36811 | 1.151 | [M+H]^+^, ([M+Na]^+^) | LPC O-18:1_A | C_26_H_54_NO_6_P | 874.1 | 0.564 | 1 |
| 0.75 | 236.5 | 508.37625 | 507.36897 | 1.15 | [M+H]^+^ | LPC O-18:1_B | C_26_H_54_NO_6_P | 911.6 | 0.302 | 0.9 |
| 1.58 | 262.5 | 594.48541 | 593.47813 | 1.281 | [M+H]^+^ | LPC O-24:0 | C_32_H_68_NO_6_P | 871.7 | -0.141 | 1.7 |
| 1.32 | 257.4 | 592.47003 | 591.46275 | 1.256 | [M+H]^+^ | LPC O-24:1 | C_32_H_66_NO_6_P | 885.1 | -0.155 | 1.2 |
| 0.69 | 224.2 | 482.32452 | 481.31725 | 1.089 | [M+H]^+^ | LPE 18:0 | C_23_H_48_NO_7_P | 777.2 | 0.841 | 1.2 |
| 1.5 | 289.9 | 804.55269 | 803.54541 | 1.423 | [M+H]^+^ | PC 38:7 | C_46_H_78_NO_8_P | 958.0 | -1.385 | 0.5 |
| 1.72 | 287.4 | 770.56811 | 769.56083 | 1.41 | [M+H]^+^ | PC 35:3 | C_43_H_80_NO_8_P | 900.0 | -1.75 | 0.1 |
| 1.63 | 286.7 | 768.55289 | 767.54696 | 1.406 | [M+H]^+^, ([M+Na]^+^) | PC 35:4 | C_43_H_78_NO_8_P | 931.1 | -1.155 | 0.6 |
| 1.47 | 276 | 678.50635 | 677.49908 | 1.351 | [M+H]^+^ | PC 28:0 | C_36_H_72_NO_8_P | 924.0 | -0.712 | 1.1 |
| 1.51 | 278.4 | 704.52183 | 703.51456 | 1.363 | [M+H]^+^ | PC 14:0_16:1 | C_38_H_74_NO_8_P | 992.9 | -0.825 | 0.9 |
| 1.56 | 283.8 | 774.52874 | 729.53054 | 1.393 | [M+HCOO]^-^ | PC 14:0_18:2_A | C_40_H_76_NO_8_P | 784.3 | -0.388 | 0.4 |
| 2.02 | 289.7 | 748.58411 | 747.57684 | 1.42 | [M+H]^+^ | PC 33:0 | C_41_H_82_NO_8_P | 515.0 | -1.3 | 0.4 |
| 1.85 | 286.9 | 746.56892 | 745.56165 | 1.406 | [M+H]^+^ | PC 33:1 | C_41_H_80_NO_8_P | 854.0 | -0.713 | 0.3 |
| 1.89 | 290.2 | 772.58468 | 771.5774 | 1.424 | [M+H]^+^ | PC 35:2 | C_43_H_82_NO_8_P | 964.0 | -0.525 | 0.5 |
| 1.63 | 286.8 | 778.53522 | 777.52794 | 1.407 | [M+H]^+^ | PC 36:6 | C_44_H_76_NO_8_P | 310.0 | -3.74 | 0.8 |
| 1.71 | 281.3 | 706.53764 | 705.53039 | 1.378 | [M+H]^+^, ([M+Na]^+^) | PC 14 :0_16 :0 | C_38_H_76_NO_8_P | 991.9 | -0.696 | 1.1 |
| 1.67 | 288.5 | 788.54454 | 743.54634 | 1.416 | [M+HCOO]^-^ | PC 15:0_18:2 | C_41_H_78_NO_8_P | 747.4 | -0.218 | 0.8 |
| 1.63 | 281 | 718.53708 | 717.5298 | 1.377 | [M+H]^+^ | PC 16:0_15:1 | C_39_H_76_NO_8_P | 992.1 | -1.322 | 0.7 |
| 1.94 | 287.1 | 734.56896 | 733.56155 | 1.407 | [M+H]^+^, ([M+Na]^+^) | PC 16:0_16:0 | C_40_H_80_NO_8_P | 996.1 | -0.645 | 1.4 |
| 1.74 | 283.7 | 732.55327 | 731.54599 | 1.39 | [M+H]^+^ | PC 16:0_16:1 | C_40_H_78_NO_8_P | 994 | -0.698 | 0.7 |
| 1.85 | 287.8 | 790.56171 | 745.56351 | 1.412 | [M+HCOO]^-^ | PC 16:0_17:1 | C_41_H_80_NO_8_P | 863.1 | 1.673 | 0 |
| 1.97 | 290.2 | 760.58546 | 759.57818 | 1.423 | [M+H]^+^ | PC 16:0_18:1_A | C_42_H_82_NO_8_P | 993.8 | 0.507 | 0.5 |
| 1.96 | 291.8 | 804.57683 | 759.57863 | 1.433 | [M+HCOO]^-^ | PC 16:0_18:1_B | C_42_H_82_NO_8_P | 876.7 | 1.029 | 0.2 |
| 2.25 | 295.8 | 776.61471 | 775.60743 | 1.451 | [M+H]^+^ | PC 35:0 | C_43_H_86_NO_8_P | 720.0 | -2.367 | 0.7 |
| 2.08 | 293.4 | 818.59205 | 773.59384 | 1.441 | [M+HCOO]^-^ | PC 17:0_18:1_A | C_43_H_84_NO_8_P | 537.0 | 0.44 | 0.03 |
| 1.84 | 291.6 | 784.58443 | 783.57638 | 1.431 | [M+H]^+^, ([M+Na]^+^, [M+K]^+^, [M+H-H_2_O]^+^) | PC 18:1_18:2 | C_44_H_82_NO_8_P | 993.0 | -0.761 | 0.8 |
| 1.74 | 290.3 | 782.56973 | 781.56245 | 1.424 | [M+H]^+^ | PC 16:0_20:4_A | C_44_H_80_NO_8_P | 993.9 | 0.379 | 0.7 |
| 1.74 | 293.2 | 826.56065 | 781.56245 | 1.44 | [M+HCOO]^-^ | PC 16:0_20:4_B | C_44_H_80_NO_8_P | 816.4 | 0.424 | 0.4 |
| 1.6 | 287.7 | 780.55348 | 779.54579 | 1.412 | [M+H]^+^, ([M+K]^+^, [M+Na]^+^) | PC 16:0_20:5_A | C_44_H_78_NO_8_P | 993.5 | -0.218 | 1 |
| 1.59 | 291.2 | 824.54412 | 779.54592 | 1.43 | [M+HCOO]^-^ | PC 16:0_20:5_B | C_44_H_78_NO_8_P | 844.8 | -0.684 | 0.6 |
| 2.13 | 296.6 | 800.61637 | 799.60909 | 1.456 | [M+H]^+^ | PC 37:2 | C_45_H_86_NO_8_P | 856.0 | -0.067 | 0.5 |
| 2.41 | 300.9 | 816.64667 | 815.63944 | 1.478 | [M+H]^+^, ([M+Na]^+^) | PC 38:1 | C_46_H_90_NO_8_P | 854.0 | -1.253 | 0.6 |
| 1.69 | 292.8 | 806.5692 | 805.56156 | 1.437 | [M+H]^+^, ([M+Na]^+^, [M+K]^+^) | PC 16:0_22:6_A | C_46_H_80_NO_8_P | 994.1 | -0.254 | 0.7 |
| 1.69 | 296.3 | 850.56039 | 805.56218 | 1.456 | [M+HCOO]^-^ | PC 16:0_22:6_B | C_46_H_80_NO_8_P | 870.3 | 0.108 | 0.6 |
| 1.56 | 280.9 | 730.53751 | 729.53037 | 1.377 | [M+H]^+^, ([M+Na]^+^) | PC 14:0_18:2_B | C_40_H_76_NO_8_P | 993.0 | -0.606 | 0.8 |
| 1.61 | 284.3 | 756.55299 | 755.54572 | 1.394 | [M+H]^+^ | PC 16:1_18:2_A | C_42_H_78_NO_8_P | 993.7 | -1.017 | 0.4 |
| 1.61 | 287.8 | 800.54422 | 755.54602 | 1.413 | [M+HCOO]^-^ | PC 16:1_18:2_B | C_42_H_78_NO_8_P | 797.1 | -0.574 | 0.2 |
| 1.67 | 283.1 | 744.55333 | 743.54731 | 1.388 | [M+H]^+^, ([M+Na]^+^) | PC 15:0_18:1 | C_41_H_78_NO_8_P | 992.0 | -0.622 | 0.3 |
| 2.18 | 293.6 | 762.59761 | 761.59033 | 1.44 | [M+H]^+^ | PC 34:0 | C_42_H_84_NO_8_P | 949.0 | -4.099 | 1.1 |
| 2.08 | 293.5 | 774.60001 | 773.5936 | 1.44 | [M+H]^+^, ([M+Na]^+^) | PC 17:0_18:1_B | C_43_H_84_NO_8_P | 992.2 | -0.981 | 1 |
| 1.89 | 292.3 | 816.57603 | 771.57782 | 1.435 | [M+HCOO]^-^ | PC 17:0_18:2 | C_43_H_82_NO_8_P | 839.8 | 0.069 | 0.3 |
| 1.78 | 287 | 758.57004 | 757.56276 | 1.408 | [M+H]^+^ | PC 16:0_18:2 | C_42_H_80_NO_8_P | 994.0 | 0.917 | 0.58 |
| 2.4 | 298.8 | 790.62806 | 789.62079 | 1.466 | [M+H]^+^ | PC 36:0 | C_44_H_88_NO_8_P | 589.0 | -4.989 | 1.0 |
| 2.2 | 296.1 | 788.61642 | 787.60915 | 1.453 | [M+H]^+^ | PC 18:0_18:1 | C_44_H_86_NO_8_P | 994.2 | 0.053 | 0.6 |
| 2.01 | 295.7 | 830.59237 | 785.59416 | 1.453 | [M+HCOO]^-^ | PC 18:0_18:2_A | C_44_H_84_NO_8_P | 876.9 | 0.851 | 0.5 |
| 2.08 | 297.8 | 812.61603 | 811.60858 | 1.462 | [M+H]^+^, ([M+Na]^+^, [M+H-H_2_O]^+^, [M+K]^+^) | PC 18:0_20:3 | C_46_H_86_NO_8_P | 994.1 | -0.433 | 0.8 |
| 1.98 | 299.3 | 854.59164 | 809.59343 | 1.471 | [M+HCOO]^-^ | PC 18:0_20:4_A | C_46_H_84_NO_8_P | 827.5 | 0.072 | 0.8 |
| 2.3 | 303.9 | 840.64675 | 839.63947 | 1.493 | [M+H]^+^ | PC 18:0_22:3 | C_48_H_90_NO_8_P | 989.8 | -1.064 | 0.9 |
| 2.15 | 303.4 | 882.62002 | 837.62182 | 1.492 | [M+HCOO]^-^ | PC 18:0_22:4_A | C_48_H_88_NO_8_P | 869.6 | -3.437 | 0.3 |
| 1.92 | 301.7 | 878.59111 | 833.59291 | 1.483 | [M+HCOO]^-^ | PC 18:0_22:6 | C_48_H_84_NO_8_P | 898.4 | -0.597 | 0.8 |
| 2.01 | 293.3 | 786.60119 | 785.59391 | 1.439 | [M+H]^+^ | PC 18: 0_18:2_B | C_44_H_84_NO_8_P | 994.0 | 0.59 | 0.7 |
| 1.88 | 298.2 | 854.59017 | 809.59197 | 1.465 | [M+HCOO]^-^ | PC 18:1_20:3_A | C_46_H_84_NO_8_P | 730 | -1.547 | 0.5 |
| 1.71 | 296.7 | 832.58371 | 831.57728 | 1.457 | [M+H]^+^, [M+Na]^+^ | PC 18:1_22:6 | C_48_H_82_NO_8_P | 993.9 | -1.614 | 1 |
| 1.64 | 288 | 782.56824 | 781.56095 | 1.413 | [M+H]^+^, ([M+K]^+^, [M+Na]^+^) | PC 18:2_18:2_A | C_44_H_80_NO_8_P | 996.2 | -1.716 | 1.9 |
| 1.63 | 291.5 | 826.55949 | 781.56129 | 1.432 | [M+HCOO]^-^ | PC 18:2_18:2_B | C_44_H_80_NO_8_P | 782.2 | -1.143 | 0.3 |
| 2.13 | 297.8 | 844.60712 | 799.60892 | 1.463 | [M+HCOO]^-^ | PC 19:0_18:2 | C_45_H_86_NO_8_P | 840.8 | -0.128 | 0.3 |
| 1.85 | 293.6 | 796.58381 | 795.57653 | 1.441 | [M+H]^+^ | PC 37:4 | C_45_H_82_NO_8_P | 909.0 | -1.55 | 0.9 |
| 2.22 | 299.3 | 814.63052 | 813.6232 | 1.47 | [M+H]^+^, ([M+Na]^+^) | PC 38:2 | C_46_H_88_NO_8_P | 950.0 | -1.829 | 0.6 |
| 1.87 | 295.1 | 810.59867 | 809.59139 | 1.449 | [M+H]^+^ | PC 18:1_20:3_B | C_46_H_84_NO_8_P | 993.0 | -2.495 | 0.5 |
| 1.98 | 296.4 | 810.59979 | 809.59229 | 1.455 | [M+H]^+^, ([M+Na]^+^, [M+K]^+^) | PC 18:0_20:4_B | C_46_H_84_NO_8_P | 994.0 | -1.104 | 0.9 |
| 1.76 | 294 | 808.58314 | 807.57587 | 1.443 | [M+H]^+^ | PC 16:0_22:5 | C_46_H_82_NO_8_P | 994.0 | -2.326 | 0.7 |
| 2.45 | 304.9 | 842.66354 | 841.65626 | 1.498 | [M+H]^+^ | PC 40:2 | C_48_H_92_NO_8_P | 741.0 | 0.231 | 0.6 |
| 2.15 | 302.1 | 838.63053 | 837.62264 | 1.484 | [M+H]^+^, ([M+Na] ^+^) | PC 18:0_22:4_B | C_48_H_88_NO_8_P | 990.0 | -1.792 | 0.87 |
| 1.91 | 299.2 | 834.5998 | 833.59238 | 1.469 | [M+H]^+^, ([M+H-H_2_O]^+^, [M+K]^+^, [M+Na]^+^) | PC 40:6 | C_48_H_84_NO_8_P | 981.0 | -1.017 | 1.3 |
| 1.55 | 293.8 | 830.56883 | 829.56156 | 1.443 | [M+H]^+^ | PC 40:8 | C_48_H_80_NO_8_P | 936.0 | -0.743 | 0.8 |
| 2.37 | 307.9 | 866.662 | 865.65472 | 1.513 | [M+H]^+^ | PC 42:4 | C_50_H_92_NO_8_P | 905.0 | -1.317 | 1.7 |
| 2.31 | 299.1 | 802.63132 | 801.62404 | 1.468 | [M+H]^+^ | PC 37:1 | C_45_H_88_NO_8_P | 845.0 | -0.661 | 0.5 |
| 2.09 | 286.4 | 764.58024 | 719.58203 | 1.405 | [M+HCOO]^-^ | PC O-16:0_16:0 | C_40_H_82_NO_7_P | 929.3 | -1.109 | 0.6 |
| 1.89 | 292 | 812.58019 | 767.58199 | 1.433 | [M+HCOO]^-^ | PC O-16:0_20:4 | C_44_H_82_NO_7_P | 898.5 | -1.129 | 0.5 |
| 2.05 | 286.6 | 762.56517 | 717.56697 | 1.406 | [M+HCOO]^-^ | PC O-16:1_16:0 | C_40_H_80_NO_7_P | 903.6 | 0.013 | 1.5 |
| 2.08 | 290.2 | 788.58063 | 743.58243 | 1.424 | [M+HCOO]^-^ | PC O-16:1_18:1 | C_42_H_82_NO_7_P | 899.1 | -0.587 | 0.6 |
| 1.89 | 288.5 | 786.56519 | 741.56699 | 1.416 | [M+HCOO]^-^ | PC O-16:1_18:2 | C_42_H_80_NO_7_P | 921.6 | -0.355 | 0.6 |
| 2.13 | 297.1 | 840.61164 | 795.61344 | 1.459 | [M+HCOO]^-^ | PC O-18:0_20:4 | C_46_H_86_NO_7_P | 911.1 | -0.883 | 0.7 |
| 2.11 | 290 | 790.5969 | 745.59869 | 1.423 | [M+HCOO]^-^ | PC O-18:1_16:0 | C_42_H_84_NO_7_P | 920.9 | -0.028 | 0.2 |
| 2.15 | 294.1 | 816.61309 | 771.61489 | 1.444 | [M+HCOO]^-^ | PC O-18:1_18:1 | C_44_H_86_NO_7_P | 644.5 | 0.214 | 0.3 |
| 2.12 | 294.3 | 814.59648 | 769.59828 | 1.445 | [M+HCOO]^-^ | PC O-18:1_18:2 | C_44_H_84_NO_7_P | 830.4 | -0.324 | 0.8 |
| 1.9 | 296 | 838.59605 | 793.59785 | 1.454 | [M+HCOO]^-^ | PC O-18:1_20:4 | C_46_H_84_NO_7_P | 937.6 | -0.873 | 0.6 |
| 0.69 | 242.7 | 550.3865 | 549.37922 | 1.182 | [M+H]^+^ | PC O-20:1 | C_28_H_56_NO_7_P | 975.5 | -0.453 | 0.3 |
| 2.09 | 289.3 | 720.58895 | 719.58167 | 1.417 | [M+H]^+^ | PC O-32:0 | C_40_H_82_NO_7_P | 994.9 | -1.707 | 1.8 |
| 1.89 | 285.8 | 718.57381 | 717.56653 | 1.4 | [M+H]^+^ | PC O-32:1_A | C_40_H_80_NO_7_P | 999.8 | -0.951 | 1.3 |
| 2.05 | 285.9 | 718.57358 | 717.5663 | 1.401 | [M+H]^+^ | PC O-32:1_B | C_40_H_80_NO_7_P | 997.2 | -1.228 | 1.4 |
| 2.11 | 293 | 746.60479 | 745.59752 | 1.436 | [M+H]^+^ | PC O-34:1 | C_42_H_84_NO_7_P | 996.2 | -1.379 | 1.7 |
| 1.93 | 288.4 | 744.58827 | 743.58099 | 1.414 | [M+H]^+^ | PC O-34:2_A | C_42_H_82_NO_7_P | 993.3 | -2.515 | 0.8 |
| 2.08 | 289.3 | 744.58933 | 743.58205 | 1.418 | [M+H]^+^ | PC O-34:2_B | C_42_H_82_NO_7_P | 988.5 | -1.104 | 1.1 |
| 1.89 | 285.4 | 742.57371 | 741.56644 | 1.399 | [M+H]^+^, ([M+Na]^+^) | PC O-34:3 | C_42_H_80_NO_7_P | 999.5 | -1.04 | 0.3 |
| 2.35 | 298.2 | 774.63669 | 773.62941 | 1.463 | [M+H]^+^ | PC O-36:1 | C_44_H_88_NO_7_P | 990.8 | -0.545 | 1.8 |
| 2.15 | 295.4 | 772.6206 | 771.61332 | 1.449 | [M+H]^+^ | PC O-36:2_A | C_44_H_86_NO_7_P | 996.7 | -1.193 | 1.3 |
| 2.31 | 296 | 772.62094 | 771.61367 | 1.452 | [M+H]^+^ | PC O-36:2_B | C_44_H_86_NO_7_P | 995.7 | -0.613 | 1.5 |
| 1.96 | 293 | 770.6038 | 769.59653 | 1.437 | [M+H]^+^ | PC O-36:3_A | C_44_H_84_NO_7_P | 997.2 | -2.637 | 1.1 |
| 2.12 | 293.6 | 770.6047 | 769.59742 | 1.44 | [M+H]^+^ | PC O-36:3_B | C_44_H_84_NO_7_P | 996.4 | -1.408 | 1.4 |
| 1.89 | 291.5 | 768.5885 | 767.58111 | 1.43 | [M+H]^+^, ([M+Na]^+^) | PC O-36:4 | C_44_H_82_NO_7_P | 998 | -2.242 | 1.3 |
| 2.13 | 297.6 | 796.62038 | 795.61305 | 1.461 | [M+H]^+^, ([M+Na]^+^) | PC O-38:4 | C_46_H_86_NO_7_P | 994.3 | -1.473 | 1.7 |
| 1.91 | 295.2 | 794.60455 | 793.59727 | 1.449 | [M+H]^+^ | PC O-38:5 | C_46_H_84_NO_7_P | 995.6 | -1.59 | 1.2 |
| 2.36 | 303.4 | 824.65167 | 823.6444 | 1.49 | [M+H]^+^ | PC O-40:4 | C_48_H_90_NO_7_P | 999.2 | -1.326 | 2 |
| 2.13 | 300.9 | 822.63561 | 821.62833 | 1.478 | [M+H]^+^ | PC O-40:5 | C_48_H_88_NO_7_P | 991.2 | -1.829 | 1.4 |
| 2.06 | 300.5 | 820.61974 | 819.61246 | 1.476 | [M+H]^+^ | PC O-40:6 | C_48_H_86_NO_7_P | 999.3 | -2.15 | 1.5 |
| 2.58 | 309.7 | 852.68442 | 851.67714 | 1.522 | [M+H]^+^ | PC O-42:4 | C_50_H_94_NO_7_P | 994.6 | 0.364 | 1.5 |
| 2.35 | 306.9 | 850.66767 | 849.66039 | 1.508 | [M+H]^+^ | PC O-42:5 | C_50_H_92_NO_7_P | 998.4 | -0.786 | 0.9 |
| 2.57 | 312.5 | 878.69857 | 877.69129 | 1.536 | [M+H]^+^ | PC O-44:5 | C_52_H_96_NO_7_P | 997.1 | -1.266 | 0.9 |
| 2.38 | 310.8 | 876.68247 | 875.67519 | 1.528 | [M+H]^+^ | PC O-44:6 | C_52_H_94_NO_7_P | 998.1 | -1.964 | 0.9 |
| 2.26 | 284.3 | 746.56838 | 745.56111 | 1.394 | [M+H]^+^ | PE 18:0_18:1 | C_41_H_80_NO_8_P | 922.5 | -1.312 | 1.9 |
| 2.08 | 281.2 | 744.55307 | 743.54579 | 1.379 | [M+H]^+^ | PE 18:0_18:2 | C_41_H_78_NO_8_P | 942.3 | -1 | 1.7 |
| 2.08 | 272.3 | 742.53884 | 743.54611 | 1.335 | [M-H]^-^ | PE 18:2_18:0 | C_41_H_78_NO_8_P | 724.4 | -0.536 | 0.7 |
| 2.37 | 283 | 730.57309 | 729.56581 | 1.387 | [M+H]^+^ | PE P -18:0 _18:1 | C_41_H_80_NO_7_P | 895.3 | -1.389 | 2.1 |
| 1.69 | 289.8 | 835.53412 | 836.5414 | 1.424 | [M-H]^-^ | PI 16:0_18:1 | C_43_H_81_O_13_P | 726.4 | -0.151 | 2.6 |
| 1.52 | 286.8 | 833.51837 | 834.52564 | 1.409 | [M-H]^-^ | PI 16:0_18:2 | C_43_H_79_O_13_P | 601.6 | -0.23 | 2.1 |
| 1.9 | 294.7 | 863.56516 | 864.57243 | 1.449 | [M-H]^-^ | PI 18:0_18:1 | C_45_H_85_O_13_P | 538.7 | -0.389 | 2.5 |
| 1.74 | 294 | 861.54933 | 862.5566 | 1.445 | [M-H]^-^ | PI 18:0_18:2 | C_45_H_83_O_13_P | 585.1 | -0.628 | 2.9 |
| 1.71 | 297.1 | 885.54924 | 886.55651 | 1.461 | [M-H]^-^ | PI 18:0_20:4 | C_47_H_83_O_13_P | 696 | -0.647 | 2.8 |
| 1.5 | 300.1 | 876.55881 | 858.52498 | 1.475 | [M+NH_4_]^+^ | PI 36:4 | C_45_H_79_O_13_P | 806.4 | -0.964 | 3.1 |
| 1.72 | 305.4 | 904.5903 | 886.55593 | 1.502 | [M+NH_4_]^+^, ([M+Na]^+^, [M+H]^+^) | PI 38:4 | C_47_H_83_O_13_P | 888.9 | -0.66 | 3.5 |
| 2.62 | 312.7 | 841.71316 | 840.70588 | 1.536 | [M+H]^+^ | SM 44:2;2O | C_49_H_97_N_2_O_6_P | 711.0 | -3.266 | 1 |
| 1.54 | 287.1 | 727.57403 | 726.56675 | 1.407 | [M+H]^+^ | SM 36:3;2O | C_41_H_79_N_2_O_6_P | 873.0 | -1.127 | 0.4 |
| 1.33 | 278.5 | 661.52752 | 660.52024 | 1.362 | [M+H]^+^ | SM 31:1;2O | C_36_H_73_N_2_O_6_P | 862.0 | -0.571 | 2.4 |
| 2.09 | 301.3 | 797.65165 | 796.64437 | 1.479 | [M+H]^+^ | SM 41:3;2O | C_46_H_89_N_2_O_6_P | 828.0 | -1.839 | 0.3 |
| 1.22 | 275.7 | 647.51194 | 646.50466 | 1.348 | [M+H]^+^ | SM 30:1;2O | C_35_H_71_N_2_O_6_P | 939.0 | -0.538 | 2.8 |
| 1.45 | 281.7 | 675.54363 | 674.53594 | 1.378 | [M+H]^+^, ([M+Na]^+^) | SM 18:1;2O/14:0 | C_37_H_75_N_2_O_6_P | 996.0 | 0.729 | 1.5 |
| 2.18 | 298.9 | 759.63664 | 758.62923 | 1.466 | [M+H]^+^, ([M+Na]^+^, [M+K]^+^) | SM 16:1;2O/22:0 | C_43_H_87_N_2_O_6_P | 994.1 | -0.949 | 1.5 |
| 1.57 | 284.5 | 689.55891 | 688.55145 | 1.393 | [M+H]^+^, ([M+Na]^+^) | SM 33:1;2O | C_38_H_77_N_2_O_6_P | 840.0 | -0.422 | 1.4 |
| 2.31 | 303.8 | 799.6679 | 798.66017 | 1.491 | [M+H]^+^, ([M+Na]^+^) | SM 18:2;2O/23:0 | C_46_H_91_N_2_O_6_P | 996.0 | -0.996 | 1.1 |
| 1.69 | 286.6 | 703.57539 | 702.56811 | 1.403 | [M+H]^+^ | SM 18:1;2O/16:0 | C_39_H_79_N_2_O_6_P | 995.3 | 0.761 | 0.5 |
| 1.93 | 293.4 | 731.60547 | 730.59842 | 1.438 | [M+H]^+^, ([M+Na]^+^) | SM 18:1;2O/18:0 | C_41_H_83_N_2_O_6_P | 993.5 | -0.943 | 0.9 |
| 2.41 | 302.9 | 787.66808 | 786.66071 | 1.486 | [M+H]^+^, ([M+Na]^+^, [M+K]^+^) | SM 18:1;2O/22:0 | C_45_H_91_N_2_O_6_P | 996.7 | -0.997 | 0.7 |
| 2.52 | 306.4 | 801.68384 | 800.67633 | 1.504 | [M+H]^+^, ([M+Na]^+^) | SM 18:1;2O/23:0 | C_46_H_93_N_2_O_6_P | 996.7 | -0.632 | 0.9 |
| 2.63 | 309.2 | 815.69944 | 814.69164 | 1.518 | [M+H]^+^, ([M+Na]^+^) | SM 18:1;2O/24:0 | C_47_H_95_N_2_O_6_P | 996.9 | -0.56 | 0.9 |
| 2.4 | 306.6 | 813.68454 | 812.67661 | 1.505 | [M+H]^+^, ([M+Na]^+^, [M+K]^+^) | SM 18:1;2O/24:1 | C_47_H_93_N_2_O_6_P | 997.3 | 0.17 | 1.1 |
| 1.49 | 283.8 | 701.55948 | 700.55263 | 1.39 | [M+H]^+^, ([M+Na]^+^, [M+K]^+^) | SM 18:2;2O/16:0 | C_39_H_77_N_2_O_6_P | 996.5 | 0.608 | 0.9 |
| 2.19 | 301.2 | 785.6522 | 784.64519 | 1.478 | [M+H]^+^, ([M+Na]^+^, [M+K]^+^) | SM 18:2;2O/22:0 | C_45_H_89_N_2_O_6_P | 996.2 | -1.103 | 1.6 |
| 1.8 | 290.3 | 717.58978 | 716.5825 | 1.422 | [M+H]^+^ | SM 35:1;2O | C_40_H_81_N_2_O_6_P | 744.0 | -1.011 | 0.4 |
| 2.48 | 309.4 | 827.69882 | 826.69155 | 1.519 | [M+H]^+^ | SM 43:2;2O | C_48_H_95_N_2_O_6_P | 886.0 | -1.484 | 0.4 |
| 1.25 | 277.2 | 673.52752 | 672.52024 | 1.357 | [M+H]^+^ | SM 32:2;2O | C_37_H_73_N_2_O_6_P | 732.0 | -0.561 | 0.8 |
| 1.61 | 286.1 | 715.57424 | 714.56696 | 1.402 | [M+H]^+^ | SM 18 :2;2O/17 :0 | C_40_H_79_N_2_O_6_P | 998.0 | -0.531 | 0.17 |
| 1.72 | 288.7 | 729.58967 | 728.58231 | 1.415 | [M+H]^+^, ([M+Na]^+^) | SM 36:2;2O | C_41_H_81_N_2_O_6_P | 816.0 | -1.077 | 0.5 |
| 1.53 | 285.2 | 677.55861 | 676.55133 | 1.396 | [M+H]^+^ | SM 32:0;2O | C_37_H_77_N_2_O_6_P | 869.0 | -0.875 | 2.3 |
| 2.05 | 295.7 | 745.62077 | 744.61349 | 1.45 | [M+H]^+^ | SM 37:1;2O | C_42_H_85_N_2_O_6_P | 855.0 | -1.452 | 0.8 |
| 2 | 301.1 | 809.65192 | 808.64464 | 1.478 | [M+H]^+^ | SM 42:4;2O | C_47_H_89_N_2_O_6_P | 801.0 | -1.51 | 0.2 |
| 1.77 | 290.5 | 705.59008 | 704.58281 | 1.423 | [M+H]^+^ | SM 34:0;2O | C_39_H_81_N_2_O_6_P | 824.0 | -0.557 | 1.2 |
| 1.97 | 295.3 | 757.62082 | 756.61354 | 1.448 | [M+H]^+^ | SM 38:2;2O | C_43_H_85_N_2_O_6_P | 867.0 | -1.28 | 0.3 |
| 2.3 | 301.1 | 773.65203 | 772.64302 | 1.477 | [M+H]^+^, ([M+Na]^+^) | SM 39:1;2O | C_44_H_89_N_2_O_6_P | 862.0 | -1.314 | 0.1 |
| 2.2 | 303.9 | 811.66802 | 810.66074 | 1.492 | [M+H]^+^ | SM 42:3;2O | C_47_H_91_N_2_O_6_P | 885.0 | -0.876 | 0.8 |
| 2.49 | 305.5 | 789.68137 | 788.67409 | 1.499 | [M+H]^+^ | SM 40:0;2O | C_45_H_93_N_2_O_6_P | 803.0 | -3.717 | 0.7 |
| 2.31 | 306.9 | 825.68297 | 824.67569 | 1.507 | [M+H]^+^ | SM 43:3;2O | C_48_H_93_N_2_O_6_P | 745.0 | -1.749 | 0.3 |
| 2.71 | 311.2 | 829.71423 | 828.70695 | 1.529 | [M+H]^+^ | SM 43:1;2O | C_48_H_97_N_2_O_6_P | 885.0 | -1.874 | 0.8 |
| 1.61 | 294 | 719.569 | 718.56172 | 1.441 | [M+H]^+^ | SM 34:1;3O | C_39_H_79_N_2_O_7_P | 838.9 | -0.954 | 3.2 |
| 2.29 | 309.8 | 831.69295 | 830.68567 | 1.522 | [M+H]^+^ | SM 42:1;3O | C_47_H_95_N_2_O_7_P | 894.1 | -2.497 | 1 |
| 2.32 | 310.5 | 829.67813 | 828.67085 | 1.525 | [M+H]^+^ | SM 42:2;3O | C_47_H_93_N_2_O_7_P | 887.1 | -1.454 | 1.8 |
| 3.14 | 306.1 | 794.72192 | 776.68764 | 1.503 | [M+NH_4_]^+^, ([M+K]^+^, [M+Na]^+^) | TG 12:0_16:0_18:1 | C_49_H_92_O_6_ | 814.1 | -1.536 | 3 |
| 3.21 | 312.5 | 822.75334 | 804.71952 | 1.535 | [M+NH_4_]^+^ | TG 14:0_16:0_18:1 | C_51_H_96_O_6_ | 899.4 | -1.378 | 2.4 |
| 3.15 | 309.8 | 820.73802 | 802.70333 | 1.521 | [M+NH_4_]^+^, ([M+K]^+^, [M+Na]^+^) | TG 14:0_16:0_18:2 | C_51_H_94_O_6_ | 700.2 | -1.015 | 1.8 |
| 3.09 | 307.8 | 818.72207 | 800.68823 | 1.511 | [M+NH_4_]^+^, ([M+Na]^+^, [M+K]^+^) | TG 14:0_16:0_18:3 | C_51_H_92_O_6_ | 234.7 | -1.415 | 1.9 |
| 3.03 | 305.3 | 816.70584 | 798.67301 | 1.499 | [M+NH_4_]^+^, ([M+K]^+^, [M+Na]^+^) | TG 12:0_18:2_18:2 | C_51_H_90_O_6_ | 798.6 | -2.104 | 2 |
| 3.1 | 311.5 | 844.73744 | 826.70361 | 1.53 | [M+NH_4_]^+^ | TG 14:0_18:2_18:2 | C_53_H_94_O_6_ | 881.8 | -1.697 | 2.6 |
| 3.23 | 315.2 | 836.7682 | 818.73436 | 1.548 | [M+NH_4_]^+^, ([M+Na]^+^) | TG 15:0_16:0_18:1 | C_52_H_98_O_6_ | 870.2 | -2.254 | 2.4 |
| 3.18 | 311 | 834.75329 | 816.71856 | 1.528 | [M+NH_4_]^+^, ([M+Na]^+^, [M+K]^+^) | TG 15:0_16:0_18:2 | C_52_H_96_O_6_ | 822.8 | -1.462 | 1.7 |
| 3.18 | 316.9 | 860.7691 | 842.73504 | 1.557 | [M+NH_4_]^+^, ([M+Na]^+^) | TG 15:0_18:1_18:2 | C_54_H_98_O_6_ | 875.9 | -1.261 | 2.1 |
| 3.26 | 314.2 | 824.76757 | 806.73346 | 1.543 | [M+NH_4_]^+^, ([M+Na]^+^) | TG 16:0_16:0_16:0 | C_51_H_98_O_6_ | 945.8 | -3.022 | 0.8 |
| 3.27 | 318.2 | 850.78402 | 832.75074 | 1.563 | [M+NH_4_]^+^, ([M+K]^+^, [M+Na]^+^) | TG 16:0_16:0_18:1 | C_53_H_100_O_6_ | 951.1 | -2.093 | 2 |
| 3.21 | 316.1 | 848.76959 | 830.73453 | 1.553 | [M+NH_4_]^+^, ([M+Na]^+^, [M+K]^+^) | TG 16:0_16:1_18:1 | C_53_H_98_O_6_ | 890.4 | -0.683 | 2.3 |
| 3.15 | 313.6 | 846.75418 | 828.7194 | 1.541 | [M+NH_4_]^+^, ([M+Na]^+^, [M+K]^+^) | TG 16:0_16:1_18:2 | C_53_H_96_O_6_ | 834.1 | -0.342 | 2 |
| 3.33 | 322.6 | 866.81221 | 848.77838 | 1.586 | [M+NH_4_]^+^ | TG 16:0_17:0_18:0 | C_54_H_104_O_6_ | 911.2 | -5.186 | 1 |
| 3.29 | 321.3 | 864.79978 | 846.76641 | 1.579 | [M+NH_4_]^+^, ([M+Na]^+^) | TG 16:0_17:0_18:1 | C_54_H_102_O_6_ | 914.7 | -1.931 | 2 |
| 3.24 | 318.4 | 862.78413 | 844.75026 | 1.565 | [M+NH_4_]^+^, ([M+Na]^+^) | TG 16:0_17:1_18:1 | C_54_H_100_O_6_ | 803.4 | -1.957 | 2.2 |
| 3.31 | 324.5 | 878.81702 | 860.78288 | 1.595 | [M+NH_4_]^+^, ([M+Na]^+^, [M+K]^+^) | TG 16:0_18:0_18:1 | C_55_H_104_O_6_ | 939.3 | 0.216 | 2.2 |
| 3.27 | 321.8 | 876.8005 | 858.76614 | 1.582 | [M+NH_4_]^+^, ([M+Na]^+^, [M+K]^+^) | TG 16:0_18:1_18:1 | C_55_H_102_O_6_ | 950.2 | -1.105 | 2.5 |
| 3.22 | 319.8 | 874.78542 | 856.75073 | 1.572 | [M+NH_4_]^+^, ([M+Na]^+^, [M+K]^+^) | TG 16:0_18:1_18:2 | C_55_H_100_O_6_ | 954.5 | -0.385 | 2.5 |
| 3.17 | 317.6 | 872.76989 | 854.7354 | 1.561 | [M+NH_4_]^+^, ([M+Na]^+^, [M+K]^+^) | TG 16:0_18:2_18:2 | C_55_H_98_O_6_ | 925.8 | -0.298 | 2.9 |
| 3.11 | 315.7 | 870.75263 | 852.71858 | 1.552 | [M+NH_4_]^+^, ([M+Na]^+^, [M+K]^+^) | TG 16:0_18:2_18:3 | C_55_H_96_O_6_ | 882.5 | -1.996 | 2.6 |
| 3.29 | 325.2 | 890.81448 | 872.78158 | 1.599 | [M+NH_4_]^+^, ([M+Na]^+^) | TG 17:0_18:1_18:1 | C_56_H_104_O_6_ | 863.4 | -2.932 | 1.8 |
| 3.24 | 322.5 | 888.79993 | 870.76611 | 1.586 | [M+NH_4_]^+^ | TG 17:0_18:1_18:2 | C_56_H_102_O_6_ | 823.7 | -1.719 | 2.6 |
| 3.19 | 321.5 | 886.78449 | 868.75066 | 1.581 | [M+NH_4_]^+^ | TG 17:1_18:1_18:2 | C_56_H_100_O_6_ | 844.7 | -1.241 | 3 |
| 3.32 | 327.9 | 904.8322 | 886.79764 | 1.613 | [M+NH_4_]^+^, ([M+Na]^+^, [M+K]^+^) | TG 18:0_18:0_18:2 | C_57_H_106_O_6_ | 431.7 | -0.522 | 3.1 |
| 3.27 | 325.7 | 902.8153 | 884.78161 | 1.602 | [M+NH_4_]^+^, ([M+K]^+^, [M+Na]^+^) | TG 18:1_18:1_18:1 | C_57_H_104_O_6_ | 947.6 | -1.882 | 2.4 |
| 3.22 | 323.8 | 900.79965 | 882.76303 | 1.592 | [M+NH_4_]^+^, ([M+Na]^+^, [M+K]^+^) | TG 18:1_18:1_18:2 | C_57_H_102_O_6_ | 914 | -2.03 | 2.8 |
| 3.31 | 331 | 930.84521 | 912.81204 | 1.628 | [M+NH_4_]^+^, ([M+Na]^+^) | TG 18:1_18:1_20:1 | C_59_H_108_O_6_ | 909 | -3.324 | 2.6 |
| 3.19 | 327.9 | 924.79945 | 906.76419 | 1.613 | [M+NH_4_]^+^, ([M+Na]^+^) | TG 18:1_18:1_20:4 | C_59_H_102_O_6_ | 630.3 | -2.273 | 2.5 |
| 3.17 | 321.7 | 898.7841 | 880.74922 | 1.582 | [M+NH_4_]^+^, ([M+Na]^+^, [M+K]^+^) | TG 18:1_18:2_18:2 | C_57_H_100_O_6_ | 901.1 | -1.818 | 2.3 |
| 3.15 | 326 | 922.78493 | 904.75111 | 1.604 | [M+NH_4_]^+^ | TG 18:1_18:2_20:4 | C_59_H_100_O_6_ | 704.8 | -1.002 | 2.5 |
| 3.35 | 335.4 | 958.87774 | 940.84391 | 1.651 | [M+NH_4_]^+^ | TG 18:1_18:2_22:0 | C_61_H_112_O_6_ | 917.9 | -2.076 | 2.2 |
| 3.39 | 338 | 986.90901 | 968.87518 | 1.664 | [M+NH_4_]^+^ | TG 18:1_18:2_24:0 | C_63_H_116_O_6_ | 840.9 | -2.013 | 1.9 |
| 3.12 | 319.6 | 896.7692 | 878.73433 | 1.572 | [M+NH_4_]^+^, ([M+Na]^+^, [M+K]^+^) | TG 18:2_18:2_18:2 | C_57_H_98_O_6_ | 936.1 | -1.124 | 2.6 |
| 3.28 | 329.1 | 928.82878 | 910.79495 | 1.619 | [M+NH_4_]^+^ | TG 18:2_18:2_20:0 | C_59_H_106_O_6_ | 323.7 | -4.33 | 2.4 |
| 3.31 | 333.7 | 956.86203 | 938.82821 | 1.642 | [M+NH_4_]^+^ | TG 18:2_18:2_22:0 | C_61_H_110_O_6_ | 777.5 | -1.947 | 2.2 |
| 3.35 | 320.4 | 862.82002 | 844.7877 | 1.575 | [M+NH_4_]^+^, ([M+Na]^+^) | TG O-18:1_16:0_18:1 | C_55_H_104_O_5_ | 856.6 | -2.247 | 2.5 |

**Table S3.** Three top metabolite pathways according to enrichment.

| **Metabolite Set Name** | **Metabolites** | **P value** |
| --- | --- | --- |
| Mitochondrial Beta-Oxidation of Short Chain Saturated Fatty Acids | Adenosine monophosphate; **L-Carnitine**; Pyrophosphate; Caprylic acid; Caproic acid; Adenosine triphosphate; **L-Octanoylcarnitine**; NAD; Octanoyl-CoA; Butyryl-CoA; 3-Hydroxybutyryl-CoA; Acetyl-CoA; FAD; Hydrogen; Coenzyme A; Acetoacetyl-CoA; NADH; Crotonoyl-CoA; Water; Hexanoyl-CoA; (S)-Hydroxyoctanoyl-CoA; 3-Oxooctanoyl-CoA; (S)-Hydroxyhexanoyl-CoA; 3-Oxohexanoyl-CoA; trans-2-Hexenoyl-CoA; (2E)-Octenoyl-CoA; Hydrogen Ion | 0.0437 |
| Phospholipid Biosynthesis | Choline; Glycerylphosphorylethanolamine; Glycerol 3-phosphate; Ethanolamine; Calcium; Magnesium; **PC(16:0/16:0)**; PS(16:0/16:0); PA(16:0/16:0); Acetylcholine; NAD; FADH; FAD; Manganese; Palmityl-CoA; Citicoline; Dihydroxyacetone phosphate; NADH; Phosphorylcholine; CDP-DG(16:0/16:0); DG(16:0/16:0/0:0); LysoPA(16:0/0:0); PE(16:0/16:0); PI(16:0/16:0); **LysoPC(16:0)**; PG(16:0/16:0); LysoPE(16:0/0:0); PGP(16:0/16:0); CL(16:0/16:0/16:0/16:0) | 0.0498 |
| Methylhistidine Metabolism | **L-Histidine**; 3-Methylhistidine; S-Adenosylhomocysteine; S-Adenosylmethionine | 0.0499 |

**Table S4.** List of significant metabolites based on PLS-DA classification and VIPS scores (>1). The presented table includes the metabolite names, corresponding m/z measurements, VIPs scores pertaining to the PLS-DA model, ANOVA-derived P-values, correlation coefficients with age, and the associated R-p-values indicating the significance of these correlations.

| **Name** | **m/z** | **VIPs** | **p-value** | **R** | **R p-value** |
| --- | --- | --- | --- | --- | --- |
| Paraxanthine | 181.07 | 1.5920 | 2.19E-05 | -0.1588 | 1.11E-01 |
| FA 18:1+1O | 295.23 | 1.5693 | 4.18E-40 | 0.0360 | 7.20E-01 |
| Dehydroepiandrosterone sulfate | 367.16 | 1.5499 | 1.14E-09 | -0.6259 | 2.00E-12 |
| Isoleucylproline | 229.15 | 1.5265 | 1.98E-07 | 0.1155 | 2.48E-01 |
| Asymmetric dimethylarginine | 203.15 | 1.4983 | 7.12E-15 | 0.2774 | 4.76E-03 |
| Methylguanine | 166.07 | 1.4922 | 1.09E-09 | 0.2861 | 3.56E-03 |
| CAR 9:0 | 302.23 | 1.4195 | 1.88E-08 | -0.1428 | 1.52E-01 |
| Methylpyridonecarboxamide | 153.07 | 1.3699 | 2.18E-10 | 0.1902 | 5.55E-02 |
| CAR 10:1 | 314.23 | 1.3475 | 5.90E-08 | -0.1174 | 2.40E-01 |
| CAR 2:0 | 204.12 | 1.3381 | 6.71E-19 | 0.2369 | 1.65E-02 |
| CAR 10:0 | 316.25 | 1.3153 | 4.28E-07 | 0.0182 | 8.56E-01 |
| Deoxycholic acid glycine conjugate | 448.31 | 1.3128 | 8.75E-04 | 0.2815 | 4.16E-03 |
| Hydroxyanthranilic acid | 154.05 | 1.3119 | 2.28E-07 | 0.2397 | 1.53E-02 |
| Indoxyl sulfate | 212.00 | 1.2672 | 2.14E-04 | -0.0687 | 4.93E-01 |
| CAR 6:0 | 260.19 | 1.2195 | 5.61E-10 | 0.1596 | 1.09E-01 |
| CAR 8:0 | 288.22 | 1.2038 | 1.87E-06 | -0.0169 | 8.66E-01 |
| Glycerophosphocholine | 258.11 | 1.1741 | 3.90E-61 | 0.0433 | 6.66E-01 |
| CAR 5:1 | 244.15 | 1.1488 | 1.23E-04 | 0.0254 | 8.00E-01 |
| Aminooctanoic acid | 160.13 | 1.1419 | 1.18E-04 | -0.0397 | 6.92E-01 |
| Histidine | 156.08 | 1.1125 | 7.08E-05 | 0.0783 | 4.34E-01 |
| Carnitine | 162.11 | 1.0481 | 4.03E-07 | 0.0528 | 5.98E-01 |
| Urobilinogen | 591.32 | 1.0133 | 1.35E-02 | 0.0931 | 3.52E-01 |

**Table S5.** List of significant lipids based on PLS-DA classification and VIPS scores (>1). The presented table includes the metabolite names, corresponding m/z measurements, VIPs scores pertaining to the PLS-DA model, ANOVA-derived P-values, correlation coefficients with age, and the associated R-p-values indicating the significance of these correlations.

| **Name** | **m/z** | **VIPs** | **p-value** | **R** | **R p-value** |
| --- | --- | --- | --- | --- | --- |
| SM 42:2;3O | 829.68 | 2.1820 | 1.71E-02 | 0.2939 | 2.72E-03 |
| LPC 18:2_A | 520.34 | 1.8945 | 1.68E-22 | -0.4070 | 2.18E-05 |
| LPC 20:3 | 546.36 | 1.8821 | 2.85E-21 | -0.2964 | 2.49E-03 |
| LPC 14:0 | 468.31 | 1.8775 | 3.96E-20 | -0.2266 | 2.20E-02 |
| LPC 18:2_B | 564.33 | 1.8629 | 6.72E-23 | -0.3796 | 8.33E-05 |
| LPC 18:3 | 518.32 | 1.7920 | 5.64E-17 | -0.2733 | 5.45E-03 |
| LPC 20:4_A | 544.34 | 1.7817 | 4.99E-18 | -0.4623 | 1.00E-06 |
| LPC O-16:0 | 482.36 | 1.7589 | 2.32E-57 | 0.0325 | 7.46E-01 |
| LPC O-18:1_A | 508.38 | 1.7491 | 5.65E-50 | 0.0577 | 5.65E-01 |
| LPC 15:0 | 482.32 | 1.7397 | 2.28E-20 | -0.1890 | 5.72E-02 |
| LPC 18:1_A | 522.36 | 1.6699 | 2.26E-32 | -0.1663 | 9.48E-02 |
| LPC O-16:1 | 480.35 | 1.6303 | 3.35E-50 | -0.0143 | 8.87E-01 |
| LPC 16:0 | 496.34 | 1.6302 | 1.14E-35 | -0.1578 | 1.13E-01 |
| TG 12:0_16:0_18:1 | 794.72 | 1.6290 | 1.24E-03 | -0.1925 | 5.25E-02 |
| LPC 18:1_B | 566.35 | 1.6217 | 4.87E-30 | -0.1794 | 7.12E-02 |
| LPC 17:0 | 510.36 | 1.6162 | 3.80E-31 | -0.2020 | 4.17E-02 |
| LPC 18:0 | 524.37 | 1.6111 | 1.58E-40 | -0.1426 | 1.53E-01 |
| PC 40:8 | 830.57 | 1.5994 | 1.98E-18 | -0.3994 | 3.21E-05 |
| PC 18:1_22:6 | 832.58 | 1.5939 | 1.04E-07 | 0.0556 | 5.79E-01 |
| PC 16:0_16:0 | 734.57 | 1.5665 | 6.52E-01 | 0.0717 | 4.74E-01 |
| LPC O-18:0 | 510.39 | 1.5381 | 1.03E-52 | 0.0386 | 7.00E-01 |
| PC O-20:1 | 550.39 | 1.5349 | 9.15E-47 | -0.0086 | 9.32E-01 |
| LPC O-18:1_B | 508.38 | 1.4866 | 2.96E-51 | 0.0134 | 8.94E-01 |
| DG 18:0_18:1 | 640.59 | 1.4812 | 5.94E-17 | -0.1279 | 2.00E-01 |
| LPC O-24:1 | 592.47 | 1.4765 | 2.71E-41 | 0.0162 | 8.71E-01 |
| LPC 16:1 | 494.32 | 1.4569 | 2.55E-20 | -0.1271 | 2.03E-01 |
| PC 16:0_22:6_B | 850.56 | 1.4499 | 6.38E-08 | -0.0268 | 7.89E-01 |
| TG 15:0_16:0_18:2 | 834.75 | 1.3995 | 2.05E-02 | -0.1609 | 1.06E-01 |
| PC O-34:1 | 746.60 | 1.3778 | 7.35E-03 | 0.1925 | 5.26E-02 |
| TG 18:1_18:2_20:4 | 922.78 | 1.3776 | 3.85E-05 | -0.0902 | 3.67E-01 |
| PC 18:0_22:6 | 878.59 | 1.3767 | 2.51E-07 | -0.0151 | 8.81E-01 |
| PC 16:0_22:6_A | 806.57 | 1.3469 | 1.53E-07 | -0.0485 | 6.28E-01 |
| PC O-18:1_20:4 | 838.60 | 1.3376 | 1.23E-06 | -0.0165 | 8.69E-01 |
| TG 18:1_18:1_20:4 | 924.80 | 1.3216 | 1.08E-06 | -0.1488 | 1.35E-01 |
| PC O-38:5 | 794.60 | 1.3203 | 3.66E-06 | -0.0204 | 8.38E-01 |
| PC O-18:1_16:0 | 790.60 | 1.2987 | 1.21E-01 | 0.1728 | 8.24E-02 |
| PC 40:6 | 834.60 | 1.2871 | 3.17E-06 | -0.0232 | 8.17E-01 |
| PC 18:2_18:2_A | 782.57 | 1.2807 | 3.61E-11 | -0.3662 | 1.53E-04 |
| TG 14:0_16:0_18:3 | 818.72 | 1.2791 | 6.11E-04 | -0.2173 | 2.82E-02 |
| LPC 20:0 | 552.40 | 1.2706 | 1.15E-32 | -0.1322 | 1.85E-01 |
| LPC 20:2 | 548.37 | 1.2650 | 7.05E-35 | -0.1816 | 6.77E-02 |
| Cer 18:1;2O/22:0 | 622.61 | 1.2524 | 3.43E-10 | -0.2111 | 3.32E-02 |
| LPC 19:0 | 538.39 | 1.2287 | 8.42E-35 | -0.0380 | 7.04E-01 |
| SM 18:1;2O/22:0 | 787.67 | 1.2189 | 5.29E-12 | -0.2709 | 5.90E-03 |
| Cer 18:1;2O/24:0 | 650.64 | 1.2073 | 1.10E-11 | -0.2540 | 1.00E-02 |
| TG 18:2_18:2_18:2 | 896.77 | 1.2037 | 4.71E-03 | -0.0523 | 6.02E-01 |
| TG 12:0_18:2_18:2 | 816.71 | 1.1877 | 1.08E-03 | -0.2195 | 2.67E-02 |
| TG 14:0_16:0_18:2 | 820.74 | 1.1780 | 3.69E-04 | -0.1861 | 6.10E-02 |
| CE 20:5 | 688.60 | 1.1727 | 3.32E-07 | -0.1148 | 2.51E-01 |
| LPC 17:1 | 508.34 | 1.1722 | 3.75E-22 | -0.1814 | 6.80E-02 |
| CE 20:4 | 690.62 | 1.1550 | 1.80E-09 | -0.2331 | 1.84E-02 |
| LPC 20:4_B | 588.33 | 1.1548 | 3.26E-16 | -0.4659 | 8.02E-07 |
| PC 16:0_22:5 | 808.58 | 1.1527 | 8.81E-11 | -0.2233 | 2.41E-02 |
| LPC 22:6 | 568.34 | 1.1513 | 8.82E-07 | -0.2434 | 1.37E-02 |
| PC O-16:0_16:0 | 764.58 | 1.1370 | 4.05E-01 | 0.1333 | 1.82E-01 |
| PC 16:0_20:4_B | 826.56 | 1.1328 | 2.75E-10 | -0.2393 | 1.54E-02 |
| SM 16:1;2O/22:0 | 759.64 | 1.1179 | 6.32E-12 | -0.2676 | 6.55E-03 |
| PC 16:0_20:5_A | 780.55 | 1.1174 | 1.80E-05 | -0.0213 | 8.32E-01 |
| PC 13:0_22:4 | 768.55 | 1.1159 | 1.68E-10 | -0.3181 | 1.12E-03 |
| LPE 18:0 | 482.32 | 1.1044 | 3.82E-17 | -0.2024 | 4.14E-02 |
| TG 14:0_18:2_18:2 | 844.74 | 1.1022 | 1.25E-04 | -0.2389 | 1.56E-02 |
| PC O-40:5 | 822.64 | 1.0959 | 5.73E-08 | -0.1505 | 1.31E-01 |
| PC O-32:0 | 720.59 | 1.0891 | 1.44E-01 | 0.0789 | 4.31E-01 |
| PC 16:0_16:1 | 732.55 | 1.0752 | 5.43E-02 | 0.2058 | 3.80E-02 |
| DG 16:0_18:1 | 612.56 | 1.0731 | 1.19E-08 | 0.0130 | 8.97E-01 |
| SM 43 :1;2O | 829.71 | 1.0614 | 2.20E-11 | -0.2895 | 3.16E-03 |
| PI 18:0_18:2 | 861.55 | 1.0516 | 3.29E-04 | 0.0550 | 5.83E-01 |
| TG 18:1_18:2_18:2 | 898.78 | 1.0484 | 2.08E-04 | -0.1780 | 7.35E-02 |
| PC 18:2_18:2_B | 826.56 | 1.0478 | 8.83E-11 | -0.3113 | 1.45E-03 |
| PC O-16:1_18:1 | 788.58 | 1.0464 | 2.35E-01 | 0.1096 | 2.73E-01 |
| PC 14:0_18:2_B | 730.54 | 1.0458 | 1.43E-11 | -0.2634 | 7.49E-03 |
| PC 36:0 | 790.63 | 1.0434 | 2.62E-08 | -0.2483 | 1.18E-02 |
| PC O-44:6 | 876.68 | 1.0422 | 1.93E-06 | -0.2675 | 6.57E-03 |
| TG 16:0_18:2_18:3 | 870.75 | 1.0403 | 1.61E-04 | -0.2373 | 1.63E-02 |
| PC 18:0_20:4_A | 854.59 | 1.0380 | 1.93E-10 | -0.2809 | 4.24E-03 |
| SM 34:0;2O | 705.59 | 1.0335 | 5.63E-01 | 0.0824 | 4.10E-01 |
| TG 18:2_18:2_22:0 | 956.86 | 1.0238 | 2.21E-01 | -0.0137 | 8.91E-01 |
| SM 18:1;2O/24:0 | 815.70 | 1.0134 | 5.98E-11 | -0.2674 | 6.58E-03 |
| PC 36:6 | 778.54 | 1.0088 | 1.59E-10 | -0.2016 | 4.22E-02 |

**References**

1 Smilde AK, Van Mechelen I. A Framework for Low-Level Data Fusion. 2019: 27–50.

2 Kennard RW, Stone LA. Computer Aided Design of Experiments. *Technometrics* 1969; **11**: 137–48.
